# The Synthesis and Base-Induced Breakdown of Triaryl 1,4-Oxathiins—An Experimental and DFT Study

**DOI:** 10.3390/molecules28176180

**Published:** 2023-08-22

**Authors:** Eric A. Nicol, Matthew Sing, Lilly U. Luu, Erwin J. Remigio, Michelle B. Mills, Adrian L. Schwan

**Affiliations:** Department of Chemistry, University of Guelph, Guelph, ON N1E 2W1, Canada

**Keywords:** oxathiin, sulfone, cyclization, computational chemistry, DFT, α-sulfonyl anion, mechanism

## Abstract

1,4-Oxathiins are valued for a breadth of bioactivities and are known commercial fungicides. This article explores a novel preparation of 2,3,6-trisubstituted 1,4-oxathiin-S,S-dioxides via the reaction of benzyl 1-alkynyl sulfones and aryl aldehydes under basic conditions. A total of 20 examples possessing exclusively a trans arrangement of the 2,3-diaryl substituents are exhibited; the products demonstrate a variation of functional groups on the aryl ring attached to the heterocyclic ring system. The preparation is hindered by the base sensitivity of the products, and a ring-opened by-product typically contaminates the reaction mixture. A DFT assessment of the overall system includes a lithium counterion and offers possible pathways for the incorporation of the aldehyde, the cyclization step and the requisite proton transfers. In addition, the DFT work reveals options for the ring opening chemistry. It appears the trans 2,3-diaryl selectivity is set during the cyclization stage of the reaction sequence. The practical work uncovers a new reaction pathway to create a family of novel 1,4-oxathiin-S,S-dioxides whereas the computational work offers an understanding of the structures and possible mechanisms involved.

## 1. Introduction

**Figure 1:**
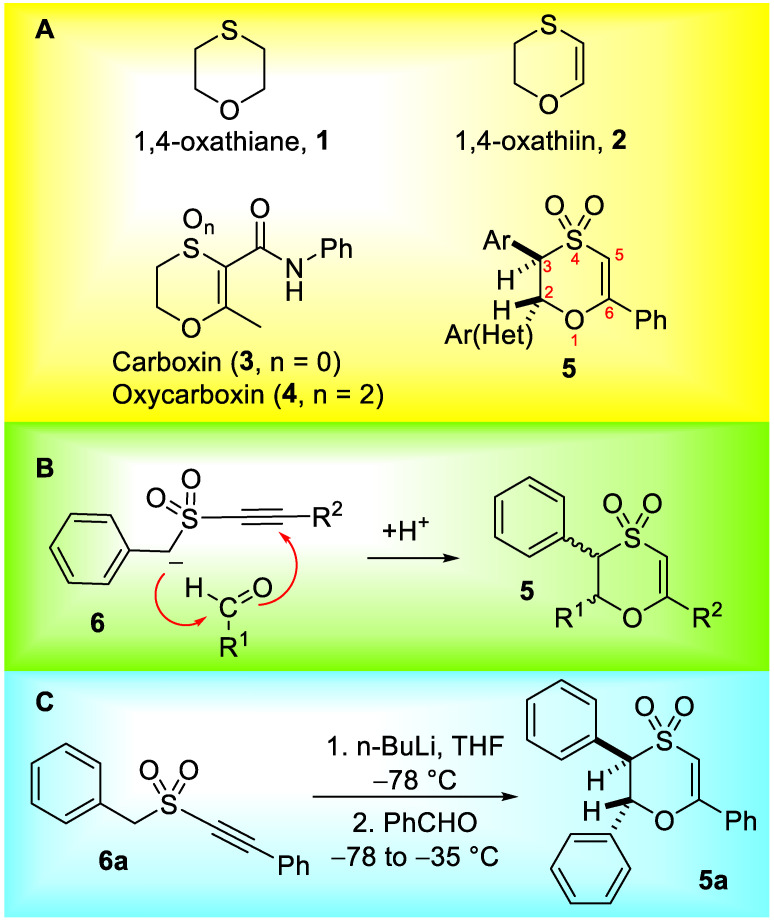
(**A**) Selected 1,4-oxathiane heterocycles. (**B**) Mechanistic conception for 1,4−oxathiin formation. (**C**) Example of *trans-*(±)-2,3-dihydro-2,3,6-triaryl-1,4-oxathiin, 4,4-dioxide formation.

6-Membered heterocycles possessing the sulfur and oxygen atoms in a 1,4 relationship (1,4-oxathiane (**1**) 1,4-oxathiiin (**2**)) [1] are valued for their biological applications and diverse chemical transformations as significant chemistry has been demonstrated for these compounds and their sulfur oxygenated analogs. The 1,4-oxathiin-4,4-dioxide skeleton is core to a family of fungicides [2,3]; compounds such as carboxin (**3**) and oxycarboxin (**4**, Figure 1A) are employed as systemic fungicides and seed treatments. This field has its origins in the authors’ city of Guelph [3,4] and remains an active area of invention [5,6,7,8,9]. The 1,4-sulfur/oxygen heterocyclic core is also part of bioactive molecules exhibiting activity against HIV-1(IIIB) [10], murine systemic candidosis and aspergillosis [11] and autoimmune diseases [12]. The 1,4-oxathiane core has been part of molecules synthesized to act as estrogen receptors [13] and for anti-viral activity [14].

The 1,4-oxathianes and -oxathiins have also been sought for access to acyclic targets [15,16] and as precursors to other heterocycles [17,18,19,20,21,22]. An oxathiin is an important component in a total synthesis of (+)-solamin [22]. The general heterocyclic ring system has been prepared through condensation-based [23] and other cyclization protocols [2,14,24,25,26,27,28], through the intermediacy of sulfenic acids [29], by cycloaddition chemistry [17,18,30,31,32,33], by multicomponent protocols [34] and through ring expansion of other heterocycles including sulfolene [35], 1,3-oxathiolanes [2,36,37,38] or oxetanes [39].

Of the protocols noted, the ring expansion chemistry of oxathiolanes, by a variety of approaches [30,36,37,38], appears to be preferred when pursuing substrates with fungicidal and related bioactivities. Those compounds generally hold a –CH_2_CH_2_– opposite the alkene of the 1,4-oxathiin. More recent preparations have focused on adding ring substitution to create carbon stereocenters and key contributions are noted here. In work from the Cossy group, a variety of *cis*-2,6-disubstituted-1,4-oxathiane 4,4-dioxides were prepared with >98:2 diastereoselectivity by the Fe (III) catalyzed cyclization of substituted bis(2-hydroxyethyl) sulfones [28]. Grainger and coworkers employed β-allyloxy and β-propargyloxy t-butyl sulfoxides as a thermal source of sulfenic acids, which were then captured by the pendant unsaturation. The chemistry formed 2,5-disubstituted-1,4-oxathiane 4-oxides with modest stereocontrol. The Capozzi/Menichetti group has shown α,α’-dioxothiones, generated in situ from thiophthalimide starting materials, can be captured in a hetero-Diels-Alder reaction with alkenes [18]. The result is a collection of 1,4-oxathiins with four substituents around the ring, and the 2,3-groups can be placed with high diastereoselectivity. Also interesting is the thermal chemistry of the sulfoxide form of the 2-alkoxy substituted oxathiins, which undergo a retro-Diels-Alder reaction when heated [17,18,32]. The electron-deficient oxosulfine so formed can be re-captured via a hetero-Diels-Alder reaction to generate alternative 1,4-oxathiins 4-oxides [18,30].

In an earlier communication [40], we introduced a fundamentally new method to assemble the 1,4-oxathiiin S,S-dioxide ring system bearing aryl groups at the 2, 3 and 6-positions of the ring and bearing the 2,3-aryl groups in trans stereochemistry (Figure 1B). Thus, the treatment of selected benzyl alkynyl sulfones (**6**) with base and an aldehyde led to the isolation of 1,4-oxathiin S,S-dioxides **5**, although in low-to-moderate yield. The experimentation in that preliminary communication served as a proof-of-principle for the heterocycle assembly. Herein, we report a more detailed development and improved yield for the preparation of a family of aryl-substituted 1,4-oxathiin S,S-dioxides. We also probe the breakdown of the ring system, as observed under the reaction conditions, and employ computational chemistry to provide an understanding of the important pathways and intermediates for both ring formation and destruction.

## 2. Results and Discussion

### 2.1. Preparation of Substituted 1,4-Oxathiins

**Scheme 1:**
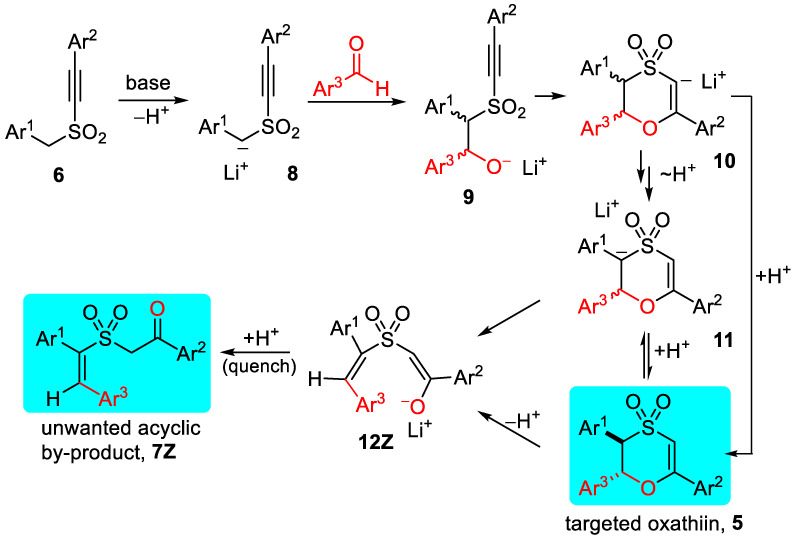
Probable mechanism for oxathiin formation and breakdown.

The previous communication introduced the cyclization reaction as represented with a specific example in Figure 1C. The products were exclusively trans (±)-2,3-dihydro-2,3,6-triaryl-1,4-oxathiin, 4,4-dioxides (**5**). The structure of the ring system was confirmed by X-ray crystal analysis of a 2,3-di-(2-iodophenyl) analog [40]. Other features indicative of the assigned structure includes a ^1^H-^1^H NMR coupling constant in the range of 11.3 to 11.7 Hz for the 2- and 3-hydrogen resonances, in keeping with a diaxial configuration of the hydrogens. Those 2- and 3-hydrogen resonances appear on the ^1^H NMR spectrum in the ranges of 5.81 to 6.48 ppm and 4.67 to 5.63 ppm, respectively. Also, consistent with the assigned structure is the lone vinylic resonance, appearing as a singlet at 5.85–6.51 ppm. Overall, the reaction requires a benzyl anion attack of the added aldehyde and oxyanion cyclization onto the triple bond followed by substrate protonation to form the product. A probable mechanism is displayed in Scheme 1. A mechanistic and computational assessment of the pathways in the scheme will be discussed later in the paper (*vide infra*).

Initial optimization protocols which led to our communicated results [40] were based primarily on the variation of the reaction temperature and on identity and equivalents of base, which eliminated NaH and K*t*OBu as viable options. The yield of **5a**, the triphenyl derivative reached a maximum yield of 54% [40]. As it turned out, the chemistry leading to **5a** proved anomalous within the domain of the initial evaluation conditions, and other examples afforded lower yields with very challenging chromatographic separation procedures, which usually incurred a loss of product. One problematic reaction component had already been identified as unreacted starting material. Another reaction constituent was characterized as an acyclic breakdown product of the 1,4-oxathiin, a recognized fate that is frequently [15,16,41], but not always [22], observed when 1,4-oxathiins are treated with base. In this case, the structure of the ring-opened product was eventually assigned to be keto sulfone **7Z** (*vide infra*).

Given the problematic reaction constituents, additional optimization experiments were undertaken to further consume starting material while minimizing the formation of the by-product (**7Z**). Reactions were performed at temperatures previously established [40] but for different reaction times. Those experiments suggested additional concerns, wherein extended reaction times led to increased formation of intractable material. A quick chromatography of those mixtures was performed to remove only the intractable reaction constituents. Mass recovery and ^1^H NMR spectrum peak integration provided ratios of the starting material, product **5a** and by-product **7Z** (Supplementary Materials, Table S1). From this analysis, six hours of reaction time offered the largest yield of the oxathiin, and that time was adopted for future reactions.

Recognizing that the base would ideally act in a catalytic manner (Scheme 1), additional experiments probed the efficiency of the heterocycle preparation using various equivalents of *n*BuLi. In those experiments, all with 2 eq. of added aldehyde, it was learned that 0.5 equiv. of base gave the best yield. Finally, a reduction of the equivalents of aldehyde improved the yield slightly more, while simplifying the constituency of the reaction mixtures (Table S2). With improved optimization and viable reaction conditions, there remained the issue of purification and isolation of the product. In TLC experiments and under subsequent flash chromatography conditions, the three reaction constituents created a difficult separation exercise. The product would often crystallize on the column thereby incurring greater losses and this outcome prompted alternative perspectives on the purification issue.

Although the oxathiins demonstrated a propensity to crystallize, a wide variety of recrystallization protocols failed to provide satisfactory results. Those recrystallization trials led to the realization that the crude reaction mixture could be triturated with warm ether to dissolve the unwanted keto-sulfone by-product (**7**) and leftover reactants while the oxathiin product resisted dissolution. The 1,4-oxathiin comprised the solid left behind and was found to be analytically pure in many cases. The ether solution was monitored for the product in each case and occasionally, small amounts of excess product were recovered through crystallization from the triturant. Another purification method was also applied in some instances. A flash column chromatography purification was initiated with a polarity that permitted crystallization of the product on the column while flushing unwanted materials. The oxathiin product was then eventually recovered in pure form by increasing the polarity of the elution solvent. The established reaction conditions with one or both purification protocols were applied to several examples, with the isolated yields of oxathiins **5** indicated in Table 1. The reactions were run on a 1–4 mmol scale. Entries #1–3 of Table 1 suggest the chemistry can be taken to a gram scale with no concerns. The examples demonstrate a variety of substituent patterns are possible on the benzyl portion of the starting benzyl alkynyl sulfone, as cyclized products are obtained in moderate to good yields (Table 1 #1–10). An assessment of substituent effects on reaction efficiency is hindered by the non-quantitative purification modes and the competing decomposition of the product. Substrates **5q-t** were contaminated by a minor (≤10%) rotational isomer, presumably due to the steric demands of the 2-iodophenyl group situated on C3 between the SO_2_ and (hetero)aryl group at the C2-position.

Variation of the aldehyde reactant created increased variability in the isolated yields (Table 1 #11–17). Heterocyclic furfural and thiophene-2-carboxaldehyde gave reduced yields (#17, 20); pyridine-2-carboxaldehyde did not yield any cyclized products. Aliphatic aldehydes with acidic α-hydrogens did not participate in the chemistry, presumably because of unwanted proton transfer and not aldehyde attack. Similarly, cinnamaldehyde and benzophenone also proved unsuitable. Use of 1-propynyl or 3,3-dimethyl-1-propynyl group as the starting alkyne component afforded product in only fair yields.

A reasonable mechanism for the product formation begins with full consumption of the sub-stoichiometric strong base (*n*BuLi or LDA) by deprotonation at the benzylic position of the starting material (Scheme 1). The consequent anion reacts with added aldehyde, to afford an alkoxide well positioned to undergo a 6-*endo-dig* conjugate addition onto the triple bond, creating a vinylic anion α to the sulfone. The cyclization may be reversible, but the cyclized form leads to the product through bimolecular proton transfer from another source, such as unreacted starting material or the carbon-3 of the product. This latter fate is presumed to lead to the undesired breakdown of the product.

As indicated, a ring-opened by-product typically contaminated the reaction mixture. The byproduct was assumed to originate from the deprotonation of the product, perhaps as a consequence of bimolecular proton transfers, followed by ring opening. During the early stages of the cyclization studies, the structure was assigned to be either **7E** or **7Z** since the availability of only one isomer did not permit a specific assignment. To pin down the structure, experiments were undertaken to probe the ring opening outcomes through deliberate α-sulfonyl deprotonation of oxathiin **5a**.

Table S3 lists several experiments in which oxathiin **5a** was treated with different bases and reaction conditions ranging from −35 °C to room temperature. The outcomes were consistently 7–25% of one double bond isomer and 75–93% of the other and the pair of isomers (**7E**/**Z**) could not be separated by preparative chromatography. The major product demonstrated ^1^H NMR resonances that matched those observed for the by-product of the **5a** preparation. However, the geometries of both isomers were still not established.

During the evaluation of various bases, it was observed that the isomeric ratio of the product demonstrated the most variability when DBU was employed. Recognizing that this tertiary base could prompt isomerization through reversible conjugate addition on the ring-opened sulfonyl ketone, the effects of this base were specifically pursued. Heating an isomeric mixture of ring-opened products with 3 equiv. of DBU in THF delivered a conversion to a single isomer after <72 h (Table 2). With a pure isomer in hand, and with a ~85% pure sample of the other isomer from the ring–opening trials, these compounds were differentiated through NOESY spectroscopy experiments (Supplementary Materials) [42,43]. The major product of ring opening possessed the Z-geometry, whereas the DBU experiments revealed the E-isomer as the thermodynamically more stable alkene. As indicated, the isomerization presumably occurs by reversible conjugate addition of DBU to Z-isomer, single bond rotation and base release. It must be mentioned that the isomerization process may occur on the enolate form of the substrate, in keeping with harsher conditions required for isomerization. The observations suggest the mode of byproduct formation during the oxathiin synthesis is a kinetically controlled ring opening and reaction conditions do not bring about any isomerization of the byproduct.

### 2.2. Computational Chemistry

Computational analyses of the synthetic and degradative pathways were performed with the Gaussian 16/Gaussview 6 software package [44] at 298 °C using the M06-2x density functional, employed in the past for the chemistry of α-sulfonyl anions [45]. Calculations were all performed on the Graham computer cluster located at the University of Waterloo as part of the Digital Research Alliance of Canada. The scratch file system where calculations were carried out is a 3.2PB parallel high-performance filesystem. The Graham cluster is 41,548 cores and 520 GPU devices. We adopted a split valence Pople basis set [6-311 + G(d,p)] with diffuse functions on non-hydrogenic atoms. The M06 functionals have been shown to provide more accurate energies and geometries than the common B3LYP functional with conformationally flexible anions [46]. Optimization and frequency calculations were carried out on reactants, pre-complexes, transition states and products for both the synthetic and degradative pathway using a CPCM solvation model with THF as the solvent. To minimize computational cost, explicit solvation was excluded from the calculations due to the computational intensity of the optimizations induced by the size of the molecular system. Transition state structures possessed a single imaginary frequency and were further validated by intrinsic reaction coordinate (IRC) calculations and/or analysis of the displacement vectors [47]. The vibrational mode for that imaginary frequency was fully consistent with the atomic motion anticipated for the reaction under analysis.

#### 2.2.1. Oxathiin Ring Formation

**Figure 2:**
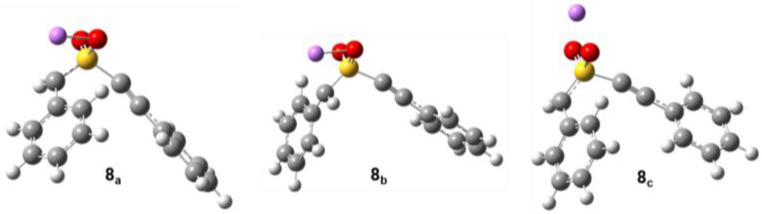
Optimized geometries of lithiated sulfone structures (**8_a–c_**). Colors: gray = C; off-while = H; red = O; yellow = S; mauve = Li

Past modelling studies of α-sulfonyl anion chemistry typically account for the metal counterion [48,49,50,51,52,53], although there are exceptions [45,54,55]. This assessment engages a lithium counterion with the α-sulfonyl anion, as part of the transition state for addition to the aldehyde and for the chemistry thereafter. It is noted that one report employing a constrained, cyclic α-sulfonyl anion found an energetically favorable transition state that did not include lithium stabilization of the oxyanion forming during attack of aldehyde [48]. In addition to employing a lithium counterion, for computational ease it was decided to model the α-sulfonyl carbanion chemistry as a monomeric entity, which has been observed among the known speciation forms in solution [56]. Three optimized structures of the lithiated sulfone (**8_a_**_–**c**_) were identified, differing in the position of the lithium counterion. The lowest energy structure (**8_c_**) had lithium as a bridging ion between the sulfonyl oxygens. Structures **8_a_** and **8_b_** were 0.2 and 0.8 kcal/mol higher in energy, respectively, and are rotational isomers about the anionic C-S bond (Figure 2). The narrow energy range is consistent with past contributions, which also report a small energetic gap among the various monomeric lithiated sulfone species [48,56].

It remained a challenge to determine a logical starting lithiated sulfone for the attack of benzaldehyde. Further complicating matters was the expectation of numerous precomplexes between the aldehyde oxygen and any of the α-lithiated sulfonyl species. To solve this issue, reasonable transition states with Li bridging the sulfonyl and carbonyl oxygens were located and intrinsic reaction coordinate analysis was used to pinpoint reactive aldehyde-lithiated sulfone precomplexes. Structures were then fully set with a final frequency/optimization; all precomplexes identified involved a form most closely related to either **8_a_** or **8_b_**.

**Figure 3:**
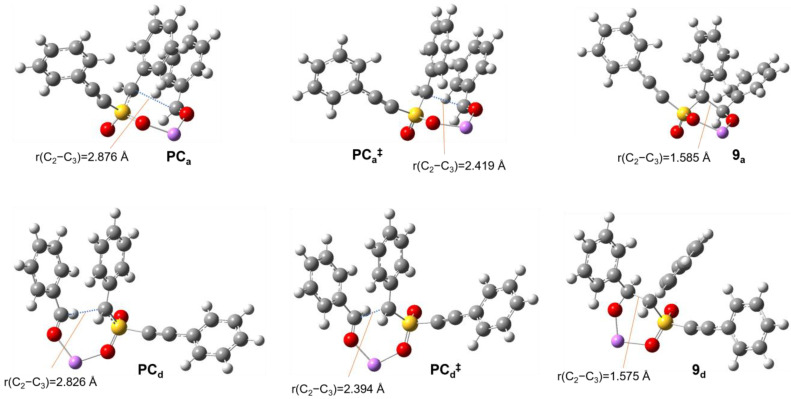
Computed structures for the addition of lithiated benzyl sulfones **8_a/b_** to benzaldehyde. ‘**a**’ series represents one of four cis addition modes. ‘**d**’ series represents one of four trans addition modes.

**Figure 4:**
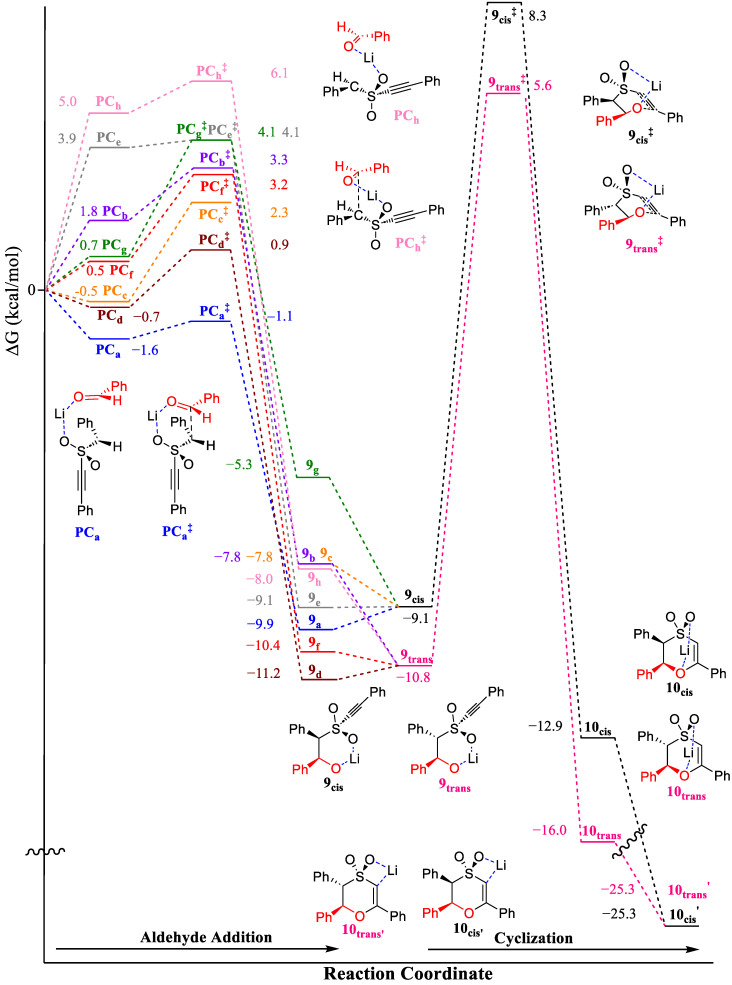
Energy profile for aldehyde addition and eventual cis and trans oxathiin synthesis.

A total of eight precomplexes (**PC_a_**-**PC_h_**) result from this approach, with two exemplified in Figure 3. With Li complexed to one sulfonyl oxygen of **8_a_**, an attack of aldehyde may occur on the si or re face. Two more structures result when the Li is complexed to the other sulfonyl oxygen of **8_a_**. The same alignments on **8_b_** create the other four isomers. Pre-complexes **PC_a,c,e,g_** eventually lead to the formation of the cis oxathiin (**5_cis_**) and similarly, pre-complexes **PC_b,d,f,h_** precede the formation of the trans oxathiin (**5_trans_**). Images of the precomplexes (**PC_a-h_**), the transition states for C_2_-C_3_-bond formation (**PC_a–h_^‡^**), and the products of the addition (**9_a–h_**) are depicted in Figures S1–S3. The relative free energies of these structures are compiled diagrammatically in a linear reaction style in Figure 4, whereas Figure S4 separates the same information into cis and trans PES manifolds. Figure 3 shows exemplary structures for the **PC_a_** to **9_a_** and **PC_d_** to **9_d_** pathways, the lowest energy options to **10_cis_** and **10_trans_** anions, respectively. At this point in the reaction, the Li counterion acts as a bridge between the oxygen of benzaldehyde and the sulfone creating a cyclic structure that permits the usage of cis and trans labels going forward along the reaction pathway. The cis/trans configurations are maintained through to the oxathiin products.

Bond lengths, atom-to-atom distances and dihedral angles for the precomplexes (**PC_a–h_**), transition states (**PC_a–h_^‡^**) and products of benzaldehyde addition can be found in Table S4 of the Supplementary Materials, alongside a discussion of key trends for these potential energy surfaces. Overall, the free energies of activation for the addition reaction are low (Figure 4 or Figure S4). Although other researchers did not probe precomplexes in their assessment of similar addition reactions [45,50], an intramolecular example of addition to a ketone displays an enthalpic barrier of 2.0 kcal/mol [45]. The addition products range from 5.3 to 11.2 kcal/mol lower in energy from the precomplexed starting materials. The two lowest energy addition products **9_d_** and **9_f_** have the phenyl groups in a trans relationship.

**Figure 5:**
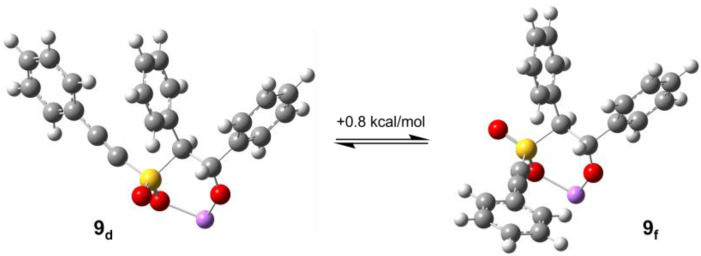
Interconversion of matched aldehyde addition products **9_d_** and **9_f_** through C3-S bond rotation and lithium repositioning.

Formation of **10** (anionic cis or trans oxathiin) requires a conjugate addition of the oxyanion to the triple bond of the alkyne. However, only half of the benzaldehyde addition products (**9_e_**_–**h**_) have the alkyne in suitable proximity. The remaining four products (**9_a_**_-**d**_) require a change in coordination of Li to the opposite sulfone oxygen, with rotation of the C_3_-S bond to place the alkyne in a pseudo-axial position. For example, the structures of trans addition products **9_d_** and **9_f_** display Ph-C_2_-C_3_-Ph dihedral angles only 1.2° in difference but differ structurally based on which sulfonyl oxygen is coordinated to the Li. However, in **9_d_**, migration of the Li the other sulfonyl oxygen and rotation around the C_3_-S is required to position the alkyne in an axial position, creating **9_f_**, a change which facilitates cyclization (Figure 5). The relative energies of these products only differ by 0.8 kcal/mol (Figure 4 and Figure 5). Similar relationships can be identified between products **9_a_**/**9_e_**, **9_b_**/**9_h_** and **9_c_**/**9_g_**. These pairs show similar dihedral angles and bond lengths, so migration of Li sulfonyl oxygen to the other, with concomitant C_3_-S bond rotation, would essentially achieve their interconversion. The difference in free energy between these pairs is 0.7, 0.2 and 2.5 kcal/mol, respectively (Figure 3 and Figure 4). To confirm the feasibility of the requisite interconversion, a transition state was sought for the C_3_-S bond rotation converting **9_d_** to **9_f_**, but optimizations never achieved convergence. However, as proof of principle, many of these transition state attempts are optimized to structures that position the alkyne in an axial position making it accessible to the oxyanion for the conjugate addition.

With the assumption these conformational changes occur as a prerequisite for cyclization, four products of benzaldehyde addition remain: **9_e_** and **9_g_** as cis adducts; **9_f_** and **9_h_** as trans adducts. While possessing identical stereogenic carbons defining them as cis adducts, **9_e_** and **9_g_** are diastereomers with sulfur as an additional stereogenic center due to Li coordination to one oxygen. By analogy with the discussion in the paragraph above, structure **9_g_** is expected to succumb to C_3_-S bond rotation, lithium repositioning and C_2_-C_3_ bond rotation to convert to **9_e_** for a free energy benefit of 3.8 kcal/mol. Trans adducts **9_f_** and **9_h_** have a similar relationship wherein structure **9_h_** would gain 2.4 kcal/mol of free energy becoming **9_f_** after C_3_-S bond rotation and lithium repositioning and a C_2_-C_3_ bond rotation that brings the C_2_-C_3_ phenyl groups from diaxial to diequatorial.

Compound **9_e_** was found to be the anionic precursor to *cis*-oxathiin formation and is renamed **9_cis_**. Entity **9_cis_** may also be accessible by direct access from other cis structures **9_a_**, **9_c_** and **9_g_**. Correspondingly, any of **9_b_**, **9_d_**, **9_f_** and **9_h_** could lead to **9_trans_**, the precursor to *trans*-oxathiin formation. The preceding discussion outlines reasonable structure changes culminating in **9_f_** which is nearly identical to **9_trans_** in terms of bond lengths and shows only minor variation in the dihedral angles based on the C_2_-C_3_ bond (Table S5). Hence, a very minor but energetically favorable structural change converts **9_f_** to **9_trans_**.

**Figure 6:**
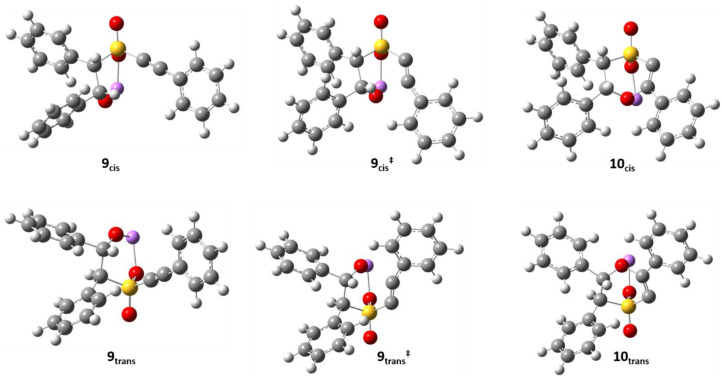
Optimized geometries of reactants, transition states and products of the cyclization process leading to cis and trans oxathiin formation.

Changes in bond lengths and dihedral angles for the cyclization processes forming **10_cis_** and **10_trans_** can be found in Table 3; optimized structures are depicted in Figure 6. Attack of the oxyanion on C_6_ of the alkyne results in a significant decrease in the distance between O_b_ and C_6_ with final bond lengths of 1.426 Å and 1.423 Å for the cis and trans products, respectively. This conjugate addition onto the alkyne is accompanied by a lengthening of the C_5_-C_6_ bond to a value of 1.344 Å, and a C_2_-O_b_ bond to 1.434 Å in both structures. Loss of charge on O_b_ results in decreased association with Li, wherein the Li-O_b_ distance increases to 2.152 Å in the trans product and 2.287 Å in the cis product. This leads to increased association with the oxygen of the sulfone with atom-to-atom distance dropping by approximately 0.7 Å. As a result of cyclization, a formal charge is passed to C_6_, contributing to a shift in the position of the Li counterion somewhat closer to that of the anionic carbon.

Relative free energy changes for cyclization are generally more significant than the prior benzaldehyde addition. The pre-cyclized compounds **9_cis_** and **9_trans_** are separated by 1.7 kcal/mol with the trans structure lower in energy. Activation barriers for the cis and trans pathways are 17.4 and 16.4 kcal/mol, respectively, with a ΔΔG^‡^ of 2.7 kcal/mol. The final products of these pathways, **10_cis_** and **10_trans_**, also show a significant energetic difference wherein the trans compound sits 3.1 kcal/mol lower than its cis counterpart. Hence, for the cyclization step leading to compound **10**, the trans pathway not only delivers a product of greater thermodynamic stability but does so with a lower free energy of activation.

**Figure 7:**
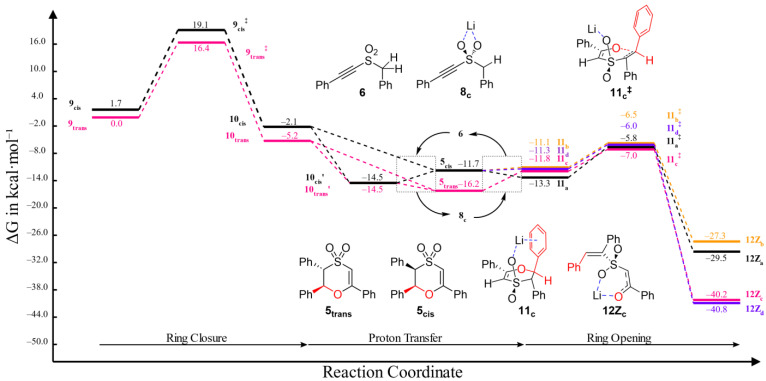
Potential Energy surfaces for proton transfer and ring opening pathways of cis- and trans-oxathiin anions. Energy entries for species **9** and **10** introduced in Figure 4 are repeated here for context.

Immediate cyclization products **10_cis_** and **10_trans_** possess the lithium still in close proximity to O_b_. It was expected that more stable forms would hold the Li nearer the sulfonyl oxygens and the vinylic carbon (C_6_). Such structures (**10_cis’_** and **10_trans’_**) were located as local minima and were found to possess identical free energies, substantially lower than **10_cis_** and **10_trans_**. Transition states for the conversion of species **10** to **10′** could not be located. A proton source can react with any of **10** or **10′** to provide neutral oxathiin, although a reaction with **10** may be expected to be faster based on its higher energy (Figure 7). Starting material **6** is logically suggested to be that proton source, as presented in Figure 7, but other options are also possible. A more complete discussion is presented after an outline of the computed ring-opening chemistry.

#### 2.2.2. Oxathiin Ring Opening

The deliberate ring openings of 2,3-dihydro-1,4-oxathiin-4,4-dioxides [41] and some aryl-substituted benzo derivatives [16] have been evaluated for their synthetic utility, as the reaction unveils a reactive vinylic sulfone. In contrast, a saturated form of the ring system survives the double deprotonation protocol necessary for a successful Ramburg-Backlund protocol delivering the 2,5-dihydrofuran core required for the successful synthesis of (+)-solamin [22]. In the work presented herein, we have observed the ring opening at an undesirable juncture during oxathiin preparation *and* when deliberately pursuing it (*vide supra*). Our practical findings grant partial understanding of the E vs Z product preferences in the ring opening. Hence, the possible ring opening mechanisms were explored computationally to help understand the formation of decomposition products **7**.

Given that deprotonated/lithiated structures of **5** would produce the ring-opened forms, a comprehensive search revealed eight optimized geometries (**11_a–h_**) for proton loss from the α-sulfonyl, benzylic position (C_3_) that gave valid PESs for ring opening (The **_a_**, **_b_**, **_c_** designation for intermediates **11** bears no correlation to earlier usage of these letters for other compounds.). See Figure S5 for all eight PES pathways; Figure S6 shows lithiated oxathiin structures and is accompanied by some comments. Table S6 offers bond lengths and angles of optimized geometries through the ring-opening process. Given that we only observed isomer **12Z** in our work, structures producing **12Z** are the principal focus of this discussion. Those pathways involve **11_a-d_** and are incorporated into Figure 7. Lithiated species **11_a,b,d_** were ostensibly derived from cis oxathiin, meaning the proton was replaced with Li^+^ before structure optimization. Structure **11c** originated from the trans oxathiin, but the structural evolution of all precursors for ring opening during optimization created anionic structures unrelated to initial lithiated starting materials.

Lithiated structures **11_a–d_** are energetically accessible via proton removal from **5_cis_** or **5_trans_** via an isodesmic pathway that engages lithiated benzyl anion **8_c_** and releases neutral starting material **6**. This is presented as an option in Figure 7 as one possible reaction for proton loss from isomers **5**. Compounds **11_a-d_** all ring open with free energy barriers of 4.8–6.8 kcal/mol, before reaching lithiated forms of **12Z** (**12Z_a–d_**) in a highly exergonic process. These energies are consistent with facile ring-opening energies of multiple structures and there is little to distinguish the four pathways. Ring-opening forms **12Z_c/d_** are lower in energy than **12Z_a/b_**, presumably due to the position of the Li atom, which bridges carbonyl and sulfonyl oxygens in the **12Z_c/d_**; all ring-opened lithiated species bring stable 1**2Z** upon reaction quenching.

The preceding discussion does not address the fate of anions **11_e–h_** since final product **12E** is not observed. However, it should be mentioned that the formation of **12E_e_** from **11_e_** may represent a valid PES. That pathway delivers a thermodynamically more stable product (**12E_e_** vs **12Z_c_**/**12Z_d_**, all with bridging Li), but has a much higher barrier (Figure S5); for energetic reasons, this is a non-competitive pathway for ring opening. Finally, consistent with the computational findings of the reactive intermediates and practical outcomes, alkene **7E** was computed to be 6.4 kcal/mol more stable than **7Z**.

#### 2.2.3. Proton Transfer Options and Overall Pathway

The collection of starting options as shown in Figure 4 suggests several similar and reversible pathways are available, culminating in the energetically preferred formation of **9_trans_** over **9_cis_** (1.7 kcal/mol). The cyclization pathway of these two entities also prefers the trans option (2.7 kcal/mol). These comparative PESs are consistent with the observed experimental selectivity of the formation of **5_trans_**. It is conceivable that additional ring opening options follow from **5_trans_** through **11_c_** to byproduct **12Z**. If any **5_cis_** is formed, it was not observed and would have to be broken down to ring opening product. It should be noted that deprotonation of **5_cis_** with a compound such as **8c** is an exergonic process. Reversibility within the all trans (or all cis) **10′**/**5**/**11** manifold is possible but there is no opportunity to interchange the *trans*-diaryl stereochemistry for the cis-diaryl configuration once the initial ring formation has occurred.

No effort was made to find transition states for proton delivery to anions **10/10′** or removal from isomers **5**. As stated, product **5_trans_** could be readily formed by protonation of vinylic anions **10_trans/_10_trans’_** by starting material **6** or another available acidic carbon among the entities in solution. As starting material depletes, a second option for a proton may be product **5**, creating one of the forms of **11**, some of which will ring open to **12Z**. Table S7 demonstrates the free energy change for most of the available proton transfer options. The most conspicuous data in Table S7 suggest that the reaction of **10_cis_** with the cis or trans forms of **5** are the most endergonic reaction depicted. The predicted free energies of reaction of 9.0–12.6 kcal/mol suggest **5_cis_** may be more prone to loss of proton. Other entries in the table are consistent with Figure 7 in that anionic **10_trans/_10_cis_** represent the most reactive deprotonating agents. If **10_trans_** reverts to **10_trans’_**, formation of product **5_trans_** is still slightly endergonic if proton transfer occurs with **5_cis_** (1.1–3.3 kcal/mol). Finally, proton transfers between products **5** and various forms of benzyl anions **8** are reasonable, based on free energies of reaction near 0 kcal/mol.

## 3. Experimental

### 3.1. General Reaction Procedures

All anhydrous reactions were carried out in glassware that was under flame-dried vacuum (or dried overnight in a 110 °C oven). Thin layer chromatography (TLC) was achieved on glass-backed plates with Silica Gel 60 (250 μm) containing a fluorescent indicator. Compounds were visualized through UV and I_2_ indicators. Flash column chromatography was carried out using silica gel supplied by Silicycle (particle size 30–63 (mesh 230–400)). ^1^H NMR and ^13^C NMR spectra were recorded on Bruker Avance 300, Avance 400 or Avance 600 spectrometer. Chemical shift values (δ) are reported in ppm relative to CDCl_3_ (δ 7.26 ppm) unless otherwise noted. The proton spectra are reported as follows: δ (multiplicity, coupling constant J, number of protons). Multiplicities are indicated by s (singlet), d (doublet), t (triplet), q (quartet) and m (multiplet). High-resolution mass spectrometry was carried out at Queen’s University, ON, Canada. Starting sulfones **6** were prepared as described previously [40,57]; characterization data for new compounds **6** are found in the Supplementary Materials. Aldehydes were purified prior to use if required.

### 3.2. Preparation of Trisubstituted 1,4-Oxathiins

#### General Method for Oxathiin-S,S-Dioxides

Under a dry, inert atmosphere, starting sulfone (1 to 4 mmol, 1.0 eq) was dissolved in THF (0.05 M) and cooled to −78 °C. *n*BuLi (1.6 M, 0.5 eq in hexanes) or LDA (0.88 M, 0.5 eq in THF) was added dropwise and stirred at −78 °C for 15 min. Aldehyde (1.5 eq) was added to the solution and the reaction mixture was warmed slowly to −35 °C. After 6 h of stirring, the reaction was quenched with NH_4_Cl (aq) and warmed to rt. The aqueous layer was extracted with EtOAc (3 × 15 mL). The combined organic layers were washed with brine, dried over MgSO_4_ and the solvent was evaporated under reduced pressure. Purification Method A: The crude residue was triturated in warm anhydrous ethyl ether several time. The decanted mixture contained impurities and the pure product remained undissolved. TLC inspection of the collected ether washes revealed if either a flash chromatography column or a 2nd trituration of the concentrated triturant was merited to obtain more product. Purification Method B: The crude residue was subjected to flash chromatography using 1:149:150 MeOH:DCM:hexanes (*v*/*v*/*v*) solvent system. After recovery of unwanted compounds, a flush with 10→30% MeOH in DCM afforded pure oxathiin.

***2,3,6-Triphenyl-2,3-dihydro-1,4-oxathiin S,S-dioxide* (5a)** was obtained as a white solid from benzyl 2-phenylethynyl sulfone and benzaldehyde using nBuLi on a 1 mmol scale, 74% yield using Method A; mp = 187–189 °C (dec.). ^1^H NMR (600 MHz, CDCl_3_), δ: 7.47–7.25 (m, 15H), 6.46 (s, 1H), 6.18 (d, J = 11.7 Hz, 1H), 4.81 (d, J = 11.7 Hz, 1H); ^13^C NMR (150.9 MHz, CDCl_3_), δ: 159.49, 134.92, 132.03, 131.52, 131.13, 129.47, 129.29, 128.79, 128.71, 128.64, 128.12, 126.38, 125.42, 101.37, 83.08, 67.96; IR (cm^−1^, neat): 3067, 1609, 1575, 1364, 1283, 1127, 1078, 910, 695; ESI HRMS, calculated for [C_22_H_18_SO_3_+H]^+^: 363.1050; found: 363.1066.

***2,6-Diphenyl-3-(2-iodophenyl)-2,3-dihydro-1,4-oxathiin S,S-dioxide* (5b)** was obtained as a white solid (2 batches) from benzyl 2-iodophenylethynyl sulfone and benzaldehyde using LDA on a 2 mmol scale, 78% yield using Method A. ^1^H NMR (600 MHz, CDCl_3_), δ: 7.71 (dd, J = 8.0, 1.0 Hz, 1H), 7.58 (d, J = 7.4 Hz, 2H), 7.46–7.02 (m, 11H), 6.85 (m, 1H), 6.39 (s, 1H), 6.07 (d, J = 11.4 Hz, 1H), 5.57 (d, J = 11.4 Hz, 1H); ^13^C NMR (150.9 MHz, CDCl_3_), δ: 159.48, 140.29, 134.34, 131.91, 131.56, 131.27, 130.69, 129,67, 129.38, 128.78, 128.67, 128.27, 128.06, 126.44, 104.48, 101.69, 83.94, 70.12; IR (cm^−1^, neat): 3068, 2931, 1609, 1575, 1495, 1451, 1307, 1283, 1120, 1078; ESI HRMS, calculated for [C_22_H_17_SO_3_I+H]^+^: 489.0016; found: 488.9979.

***2,6-Diphenyl-3-(3-chlorophenyl)-5,6-dihydro-1,4-oxathiin S,S-dioxide* (5c)** was obtained as a white solid from 3-chlorobenzyl 2-phenylethynyl sulfone and benzaldehyde using nBuLi on a 3 mmol scale, 76% yield using Method B; mp = 173.5–174.9 °C (dec.). ^1^H NMR (600 MHz, CDCl_3_), δ: 7.44–7.21 (m, 14H), 6.48 (s, 1H), 6.16 (d, J = 11.7 Hz, 1H), 4.79 (d, J = 11.7 Hz, 1H); ^13^C NMR (100.6 MHz, CDCl_3_), δ: 159.61, 134.50, 134.48, 131.81, 131.65, 131.14, 129.80, 129.72, 129.56, 129.24, 128.89, 128.82, 128.03, 127.43, 126.39, 101.16, 82.89, 67.39; IR (cm^−1^, neat): 3067, 3035, 1608, 1574, 1302, 1280, 1127, 1081 cm^−1^; Analysis calculated for C_22_H_17_ClO_3_S: C, 66.58; H, 4.32; found: C, 66.54; H, 4.41.

***2,6-Diphenyl-3-(4-nitrophenyl)-2,3-dihydro-1,4-oxathiin S,S-dioxide* (5d)** was obtained as a white solid from 4-nitrobenzyl 2-phenylethynyl sulfone and benzaldehyde using nBuLi on a 3 mmol scale, 62% yield using Method B; mp = 213–215 °C (dec.). ^1^H NMR (400 MHz, CDCl_3_), δ: 8.11 (d, J = 8.8 Hz, 2H), 7.63 (m, 2H), 7.55–7.48 (m, 3H), 7.44–7.40 (m, 2H), 7.31–7.29 (m, 5H) 6.47 (s, 1H), 6.18 (d, J = 11.6 Hz, 1H), 4.92 (d, J = 11.6 Hz, 1H); ^13^C NMR (100.6 MHz, CDCl_3_), δ: 159.86, 148.26, 134.09, 133.01, 132.06, 131.80, 131.59, 130.00, 129.06, 128.85, 127.93, 126.38, 123.58, 101.09, 82.80, 67.49; IR (cm^−1^, neat): 3074, 1609, 1522, 1347, 1295, 1128, 1082 cm^−1^; ESI HRMS, calculated for [C_22_H_17_SO_5_N+H]^+^: 408.0900; found: 408.0894.

***2,6-Diphenyl-3-(4-methylphenyl)-2,3-dihydro-1,4-oxathiin S,S-dioxide* (5e)** was obtained as a white solid from 4-methylbenzyl 2-phenylethynyl sulfone and benzaldehyde using nBuLi on a 1 mmol scale, 76% yield using Method A; mp = 225.5–226.3 °C (dec.). ^1^H NMR (600 MHz, CDCl_3_), δ: 7.64 (m, 2H), 7.48 (m, 1H), 7.42 (m, 2H), 7.36 (m, 2H), 7.31 (m, 3H), 7.23 (m, 2H), 7.09 (m, 2H), 6.48 (s, 1H), 6.19 (d, J = 11.7 Hz, 1H), 4.82 (d, J = 11.7 Hz, 1H), 2.28 (s, 3H); ^13^C NMR (100.6 MHz, CDCl_3_), δ:159.44, 139.27, 135.06, 132.09, 131.47, 130.91, 129.43, 128.77, 128.72, 128.18, 126.36, 122.08, 101.38, 83.01, 67.65, 21.23; IR (cm^−1^, neat): 3067, 3034, 2922, 1609, 1574, 1301, 1283, 1126, 1079; Analysis calculated for C_23_H_20_O_3_S: C, 73.38; H, 5.36; found: C, 73.56; H, 4.99.

***2,6-Diphenyl-3-(4-methoxyphenyl)-2,3-dihydro-1,4-oxathiin S,S-dioxide* (5f)** was obtained as a white solid from 4-methoxybenzyl 2-phenylethynyl sulfone and benzaldehyde using nBuLi on 0.9 mmol scale, 44% yield using Method A; mp = 163.2–164.2 °C (dec.). ^1^H NMR (600 MHz, CDCl_3_), δ: 7.44–7.27 (m, 12H), 6.71 (m, 2H), 6.38 (s, 1H), 6.06 (d, J = 11.7 Hz, 1H), 4.70 (d, J = 11.7 Hz, 1H), 3.66 (s, 3H); ^13^C NMR (100.6 MHz, CDCl_3_), δ: 160.22, 159.45, 132.12, 131.47, 131.17, 129.54, 128.77, 128.64, 128.14, 127.46, 127.08, 126.37, 114.21, 101.27, 83.06, 67.28, 55.19; IR (cm^−1^, neat): 3056, 2967, 1610, 1328, 1305, 1253, 1127, 1081 cm^−1^; Analysis calculated for C_23_H_20_O_4_S: C, 70.39; H, 5.14; found: C, 70.50; H, 5.21.

***2,6-Diphenyl-3-(4-bromophenyl)-2,3-dihydro-1,4-oxathiin S,S-dioxide* (5g)** was obtained as a white solid from 4-bromobenzyl 2-phenylethynyl sulfone and benzaldehyde using nBuLi on a 0.9 mmol scale, 73% yield using Method A; mp = 193.5–194.2 °C (dec.). ^1^H NMR (400 MHz, CDCl_3_), δ: 7.51–7.26 (m, 15H), 6.49 (s, 1H), 6.24 (d, J = 11.7 Hz, 1H), 4.88 (d, J = 11.7 Hz, 1H); ^13^C NMR (100.6 MHz, CDCl_3_), δ: 159.48, 134.36, 132.82, 132.02, 131.53, 131.32, 130.83, 129.48, 129.06, 128.91, 128.64, 127.30, 126.39, 101.45, 83.06, 57.70; IR (cm^−1^, neat): 3066, 2369, 1663, 1647, 1608, 1489, 1458, 1126, 1072; ESI HRMS, calculated for [C_22_H_17_^79^BrO_3_S+NH_4_]^+^: 458.0420; found: 458.0407.

***2,6-Diphenyl-3-(3-cyanophenyl)-2,3-dihydro-1,4-oxathiin S,S-dioxide* (5h)** was obtained as a white solid (after trituration and chromatography of the dissolved materials) from 3-cyanobenzyl 2-phenylethynyl sulfone and benzaldehyde using nBuLi on a 3 mmol scale, 64% yield using Method A; mp = 210.2–211.0 °C (dec.). ^1^H NMR (600 MHz, CDCl_3_), δ: 7.61–7.39 (m, 14H), 6.49 (s, 1H), 6.17 (d, J = 11.7 Hz, 1H), 4.86 (d, J = 11.7 Hz, 1H); ^13^C NMR (100.6 MHz, CDCl_3_), δ: 159.61, 134.49, 134.46, 131.81, 131.65, 131.15, 129.80, 129.72, 129.57, 129.23, 128.82, 128.82, 128.03, 127.42, 126.39, 101.17, 82.89, 67.41; IR (cm^−1^, neat): 3067, 3036, 1609, 1574, 1302, 1280, 1127, 1081 cm^−1^; ESI HRMS, calculated for [C_23_H_17_NSO_3_+H]^+^: 388.1002; found: 388.1272.

***3,6-Diphenyl-2-(4-cyanophenyl)-2,3-dihydro-1,4-oxathiin S,S-dioxide* (5i)** was obtained as a white solid (two batches) from benzyl 2-phenylethynyl sulfone and 4-cyanobenzaldehyde using nBuLi on a 1 mmol scale, 69% yield using Method A; mp = 215.1–215.9 °C (dec.). ^1^H NMR (600 MHz, CDCl_3_), δ: 7.50–7.19 (m, 15H), 6.41 (s, 1H), 6.15 (d, J = 11.6 Hz, 1H), 4.67 (d, J = 11.6 Hz, 1H); ^13^C NMR (100.6 MHz, CDCl_3_), δ: 159.19, 139.85, 132.52, 131.81, 131.59, 130.94, 129.80, 129.00, 128.92, 128.69, 126.31, 124.78, 117.95, 113.47, 101.92, 82.19, 67.77; IR (cm^−1^, neat): 3061, 2998, 2946, 2228, 1679, 1596, 1447, 1276, 1125, 1074; Analysis calculated for C_23_H_17_NO_3_S: C, 71.30; H, 4.42; found: C, 71.24; H, 4.67.

***3,6-Diphenyl-2-(4-bromophenyl)-2,3-dihydro-1,4-oxathiin S,S-dioxide* (5j)** was obtained as a white solid from benzyl 2-phenylethynyl sulfone and 4-bromobenzaldehyde using nBuLi on a 1 mmol scale, 34% yield using method A. mp = 164.5–165.3 °C (dec.). ^1^H NMR (400 MHz, CDCl_3_), δ: 7.54–7.13 (m, 14H), 6.39 (s, 1H), 6.08 (d, J = 11.7 Hz, 1H), 4.69 (d, J = 11.7 Hz, 1H); ^13^C NMR (100.6 MHz, CDCl_3_), δ: 159.32, 134.01, 131.99 131.83, 131.64, 131.05, 129.67, 129.54, 128.84, 126.33, 125.13, 123.69, 101.57, 82.38, 67.77; IR (cm^−1^, neat): 3066, 2921, 1609, 1574, 1286, 1220, 1126, 1012; Analysis calculated for C_22_H_17_BrO_3_S: C, 59.87; H, 3.88; found: C, 60.0; H, 3.96.

***3,6-Diphenyl-2-(4-fluorophenyl)-2,3-dihydro-1,4-oxathiin S,S-dioxide* (5k)** was obtained as a white solid (after trituration and chromatography of the dissolved materials) from benzyl 2-phenylethynyl sulfone and 4-fluorobenzaldehyde using nBuLi on a 1 mmol scale, 78% yield using Method A. mp = 198.0–198.8 °C (dec.). ^1^H NMR (400 MHz, CDCl_3_), δ: 7.64–7.00 (m, 12H), 76.98 (t, J = 2.2 Hz, 2H), 6.49 (s, 1H), 6.20 (d, J = 11.7 Hz, 1H), 4.80 (d, J = 11.7 Hz, 1H); ^13^C NMR (100.6 MHz, CDCl_3_), δ: 162.99 (d, ^1^J = 247.5 Hz), 159.36, 131.92, 131.60, 131.07, 130.95 (d, ^4^J = 3.6 Hz), 129.96 (d, ^3^J = 8.5 Hz), 129.43, 128.84, 128.76, 126.35, 125.32, 115.86 (d, ^2^J = 21.7 Hz), 101.52, 82.34, 67.99; IR (cm^−1^, neat): 3067, 2926, 1607, 1330, 1318, 1286, 1220, 1128; Analysis calculated for C_22_H_17_FO_3_S: C, 69.46; H, 4.50; found: C, 69.49; H, 4.78.

***3,6-Diphenyl-2-(4-nitrophenyl)-2,3-dihydro-1,4-oxathiin-S,S-dioxide* (5l)** was obtained as a white solid from benzyl 2-phenylethynyl sulfone and 4-nitrobenzaldehyde using nBuLi on a 1 mmol scale, 62% yield using method A. mp = 146–148 °C; ^1^H-NMR (400 MHz, CDCl_3_) δ: 8.15 (d, J = 8.8 Hz, 2H), 7.63 (m, 2H), 7.53 (d, J= 8.8 Hz, 2H), 7.43 (m, 3H), 7.31 (m, 5H), 6.50 (s, 1H), 6.29 (d, J= 11.6 Hz, 1H), 4.79 (d, J= 11.6 Hz, 1H); ^13^C-NMR (100.6 MHz, CDCl_3_) δ: 159.10, 148.22, 141.59, 131.80, 131.47, 130.89, 129.82, 129.0, 128.94, 128.89, 126.25, 124.62, 123.89, 101.87, 81.90, 67.72; IR (neat, cm^−1^): 3075, 1609, 1574, 1495, 1349, 1304, 1263, 1140, 1074, 897, 749; ESI HRMS, calculated for [C_22_H_17_NO_5_S+H]^+^: 408.0900; found: 408.0923.

***3,6-Diphenyl-2-(2-triflouromethylphenyl)-2,3-dihydro-1,4-oxathiin S,S-dioxide* (5m)** was obtained as a white solid from benzyl 2-phenylethynyl sulfone and 2-trifluoromethylbenzaldehyde using nBuLi on a 2 mmol scale, 35% yield using Method A. mp = 188.0–188.8 °C (dec.). ^1^H NMR (600 MHz, CDCl_3_), δ: 7.62 (d, J = 7.8 Hz, 1H), 7.45–7.26 (m, 10H), 7.20–7.18 (m, 3H), 6.56 (d, J = 11.5 Hz, 1H), 6.37 (s, 1H), 5.02 (d, J = 11.5 Hz, 1H), 4.80 (d, J = 11.7 Hz, 1H); ^13^C NMR (100.6 MHz, CDCl_3_), δ: 159.49, 132.54, 132.32, 131.88, 131.61, 131.04, 129.82, 129.71, 129.42, 128.85, 128.80, 124.95, 123.90 (q, J = 271.5 Hz, CF_3_), 101.94, 67.02; IR (cm^−1^, neat): 3070, 2933, 1609, 1312, 1222, 1165; Analysis calculated for C_23_H_17_F_3_O_3_S: C, 64.18; H, 3.98; C, 64.80; H, 3.90.

***3,6-Diphenyl-2-(4-methoxyphenyl)-2,3-dihydro-1,4-oxathiin S,S-dioxide* (5n)** was obtained as a white solid from benzyl 2-phenylethynyl sulfone and 4-methoxybenzaldehyde using nBuLi on a 2 mmol scale, 74% yield using Method A. mp = 164.5–165.0 °C (dec.). ^1^H NMR (400 MHz, CDCl_3_), δ: 7.38–7.16 (m, 11H), 6.70 (dd, J = 6.8 & 1.0 Hz, 2H), 6.36 (s, 1H), 6.06 (d, J =11.7 Hz, 1H), 4.73 (d, J = 11.7 Hz, 1H), 3.65 (s, 3H); ^13^C NMR (100.6 MHz, CDCl_3_), δ: 160.18, 159.50, 132.12, 131.47, 131.17, 129.54, 129.21, 128.77, 128.64, 128.14, 127.46, 127.08, 126.39, 125.61, 114.40, 114.08, 101.24, 82.70, 67.91, 55.23; IR (cm^−1^, neat): 3067, 2838, 1610, 1575, 1516, 1283, 1251, 1178, 1126; Analysis calculated for C_23_H_20_O_4_S: C, 70.39; H, 5.14; found: C, 70.07; H, 5.24.

***3,6-Diphenyl-5-(2-furyl)-2,3-dihydro-1,4-oxathiin S,S-dioxide* (5o)** was obtained from benzyl 2-phenylethynyl sulfone and furfural, using nBuLi on a 2 mmol scale, 37% yield using Method A; mp = 144–146 °C (dec.). ^1^H NMR (600 MHz, CDCl_3_), δ: 7.63 (d, J = 7.8 Hz, 2H), 7.48–7.26 (m, 9H), 6.42 (s, 1H), 6.39 (s, 1H), 6.24, (d, J = 11.7 Hz, 1H), 6.23 (s, 1H), 4.96 (d, J = 11.7 Hz, 1H); ^13^C NMR (150.9 MHz, CDCl_3_), δ: 159.25, 147.37, 143.91, 131.94, 131.56, 130.72, 129.44, 128.44, 128.79, 128.63, 126.43, 125.20, 112.18, 110.49, 101.52, 65.61; IR (cm^−1^, neat): 3070, 1609, 1575, 1496, 1283, 1127, 1078; ESI HRMS, calculated for [C_20_H_16_SO_4_+H]^+^: 353.0843; found: 353.0878.

***3-(4-Methylphenyl)-2-(4-nitrophenyl)-6-phenyl-2,3-dihydro-1,4-oxathiin S,S-dioxide* (5p)** was obtained as a white solid from 4-methylbenzyl 2-phenylethynyl sulfone and 4-nitrobenzaldehyde using nBuLi on a 1 mmol scale, 55% yield using Method A; mp = 213–215 °C (dec.). 1H NMR (400 MHz, CDCl_3_), δ: 8.16 (d, J = 8.8 Hz, 2H), 7.64–7.62 (m, 2H), 7.56–7.51 (m, 3H), 7.47–7.43 (m, 2H), 7.23 (d, J = 8.2 Hz, 2H), 7.11 (d, J = 8.2 Hz, 2H), 6.51 (s, 1H), 6.29 (d, J = 11.7 Hz, 1H), 4.75 (d, J = 11.7 Hz, 1H), 2.29 (s, 3H); ^13^C NMR (100.6 MHz, CDCl_3_), δ: 159.12, 148.29, 141.80, 139.90, 131.78, 131.61, 129.81, 129.04, 128.92, 126.30, 123.94, 121.37, 101.99, 81.86, 67.52, 21.24; IR (cm^−1^, neat): 3073, 2943, 2859, 1609, 1575, 1447, 1303, 1281, 1221, 117, 1082 cm^−1^; ESI HRMS, calculated for [C_23_H_19_NO_5_S+NH]^+^: 439.1322; found: 439.1310.

***3-(2-iodophenyl)-2-(4-nitrophenyl)-6-phenyl-2,3-dihydro-1,4-oxathiin S,S-dioxide* (5q)** was obtained as a white solid from 4-methylbenzyl 2-phenylethynyl sulfone and 4-nitrobenzaldehyde using LDA on a 1 mmol scale, 64% yield using Method B; mp = 185–187 °C; ^1^H NMR (400 MHz, CDCl_3_) δ: 8.14 (d, J= 8.8 Hz, 2H), 7.78 (dd, J= 1.2 & 8.0 Hz, 1H), 7.63 (m, 3H), 7.57 (m, 3H), 7.46 (m, 2H), 7.33 (m, 1H), 6.95 (m, 1H), 6.51 (s, 1H), 6.23 (d, J= 11.3 Hz, 1H), 5.63 (d, J= 11.3 Hz, 1H); ^13^C NMR (100.6 MHz, CDCl_3_) δ: 159.21, 140.95, 140.73, 131.96, 131.93, 131.45, 131.27, 130.88, 129.20, 128.94, 128.64, 128.39, 126.38, 123.86, 104.46, 102.28, 82.66, 69.92; IR (neat, cm^−1^): 3055, 2987, 2305, 1600, 1574, 1521, 1447, 1422, 1346, 1322, 1304, 1283, 1223, 1130, 1086, 896; ESI HRMS, calculated for [C_22_H_16_INO_5_S+H]+: 533.9967; found: 533.9857.

***3-(2-Iodophenyl)-6-phenyl-2-(2-thienyl)-2,3-dihydro-1,4-oxathiin S,S-dioxide* (5r)** was obtained as a white solid from 2-iodobenzyl 2-phenylethynyl sulfone and thiophene-2-carboxaldehyde using LDA on a 4 mmol scale, 35% yield using Method B; mp = 163–165 °C. ^1^H NMR (400 MHz, CDCl_3_): δ 7.86 (dd, *J* = 8.0 & 1.2 Hz, 1H), 7.68 (dd, *J* = 8.3 & 1.5 Hz, 2H), 7.52–7.30 (m, 6H), 7.06 (dd, *J* = 0.8 & 1.2 Hz, 1H), 7.01 (td, *J* = 1.2 & 7.8 Hz, 1H), 6.88 (m, 1H), 6.50 (s, 1H), 6.48 (d, *J* = 11.3 Hz, 1H), 5.61 (d, *J* = 11.3 Hz, 1H); ^13^C NMR (100 MHz, CDCl_3_): δ 159.1, 140.4, 136.7, 131.6, 131.1, 130.8, 129.2, 128.8, 128.6, 128.1, 127.6, 126.8, 126.4, 104.6, 101.7, 78.9, 70.7. IR (cm^−1^, neat): 3071, 2930, 1604, 1573, 1495, 1448, 1308, 1278, 1129, 1066; ESI HRMS, calculated for [C_20_H_15_IO_3_S_2_+H]^+^: 494.9586; found: 494.9580.

***6-(t-Butyl)-3-(2-iodophenyl)-2-phenyl-2,3-dihydro-1,4-oxathiin S,S-dioxide* (5s)** was obtained as a white solid from 2-iodobenzyl 3,3-dimethylbutynyl sulfone and benzaldehyde using LDA on a 3 mmol scale, 39% yield using Method A; mp = 180–182 °C (dec.). ^1^H NMR (600 MHz, CDCl_3_), δ: 7.66 (dd, J = 8.4 & 1.2 Hz, 1H), 7.42 (dd, J = 8.1 & 1.8 Hz, 1H), 7.20–7.17 (m, 6H), 6.82 (dt, J = 7.8, 1.2 Hz, 1H), 5.85 (s, 1H), 5.81 (d, J = 11.5 Hz, 1H), 5.19 (d, J = 11.5 Hz, 1H), 1.12 (s, 9H); ^13^C NMR (150.9 MHz, CDCl_3_), δ: 171.40, 140.25, 134.62, 131.14, 130.62, 129.52, 129.48, 128.56, 128.03, 127.98, 104.45, 100.08, 83.45, 69.75, 37.27, 27.48; IR (cm^−1^, neat): 2967, 1608, 1468, 1305, 1287, 1218, 1136, 1103; ESI HRMS, calculated for [C_20_H_21_SO_3_I+H]^+^: 469.0329; found: 469.0310.

***6-(2-Iodophenyl)-5-phenyl-3-methyl-5,6-dihydro-1,4-oxathiin S,S-dioxide* (5t)** was obtained as a white solid from sulfone 2-iodobenzyl 1-propynyl sulfone and benzaldehyde using nBuLi on a 1 mmol scale, 38% yield using Method A; mp = 165–166 °C (dec.). ^1^H NMR (600 MHz, CDCl_3_), δ: 7.76 (dd, J = 8.0 & 2.2 Hz, 1H), 7.45 (dd, J = 8.0 & 1.6 Hz, 1H), 7.29 (m, 6H), 6.92 (m, 1H), 5.96 (d, J = 11.4 Hz, 1H), 5.89 (s, 1H), 5.50 (d, J = 11.4 Hz, 1H), 2.08 (s, 3H); ^13^C NMR (100.6 MHz, CDCl_3_), δ: 161.54, 140.25, 134.26, 131.15, 130.65, 129.68, 129.347, 128.68, 128.18, 128.06, 104.48, 103.10, 83.63, 69.42, 21.39; IR (cm^−1^, neat): 3064, 2924, 2853, 1626, 1468, 1305, 1288, 1206, 1165, 1117; Analysis calculated for C_17_H_15_O_3_IS: C, 47.90; H, 3.55; found: C, 48.11; H, 3.70.

### 3.3. Oxathiins Ring Opening Experiments

#### 3.3.1. Example Procedure for Base Catalyzed Ring Openings of Oxathiin **5a**

Oxathiin **5a** (1 eq., 0.41 mmol) was dissolved in dry THF (0.041 M, 10 mL), after which one equivalent of the base was added at −35 °C (monitored by TLC). All reactions were completed in 24 h, whereupon EtOAc was added and the mixture was poured into NH4Cl. The organic layer was washed with brine and dried over magnesium sulfate. The solvent was removed under reduced pressure. There was 100% consumption of **5a**, providing **12Z** as the major isomer in 67% total yield. ^1^H NMR (600 MHz, CDCl_3_) δ: 7.79 (dd, J = 1.3 & 8.2 Hz, 2H), 7.72–7.69 (m, 2H), 7.61–7.56 (m, 3H), 7.46–7.42 (m, 4H), 7.38–7.3 (m, 4H), 4.34 (2H, s); ^13^C NMR (100.6 MHz, CDCl_3_) δ: 198.97, 188.54, 140.73, 139.02, 134.29, 133.92, 131.25, 130.79, 129.85, 129.29, 128.87, 128.70, 128.59, 128.51, 127.79, 57.74; IR (neat, cm^−1^): 3065, 2927, 1682, 1598, 1448, 1323, 1277, 1145, 1125; ESI HRMS, calculated for [C_22_H_16_O_3_S+H]^+^: 363.1049 found: 363.1050.

#### 3.3.2. Example Procedure for Isomerization of **12Z** to **12E**

One equivalent (0.37 mmol, of **12Z**/**12E** mixture (dominated by **12Z**) was placed in dry THF (0.041 M) and DBU (3 eq., 1.11 mmol) was then added. The reaction was then heated to 50 °C and monitored for 72 h. The reaction was then taken up in 15 mL of EtOAc, and the organic layer was washed with water 3 times to remove the amidine. The organic layer was then washed with brine and dried over magnesium sulfate. Volatiles were then removed under reduced pressure to afford **12E**, 82%. ^1^H NMR (600 MHz, CDCl_3_) δ: 7.96 (m, 2H), 7.71 (s, 1H), 7.64–7.60 (m, 3H), 7.52–7.47 (m, 5H), 7.27 (m, 1H), 7.19 (m, 2H), 7.07 (m, 2H), 4.46 (2H, s); ^13^C NMR (150.6 MHz, CDCl_3_): δ 188.54, 140.73, 138.29, 136.11, 134.32, 132.52, 131.25, 130.95, 130.80, 130.32, 129.86, 129.47, 129.30, 128.89, 128.57, 128.52, 57.74; IR (neat, cm^−1^): 3052, 2924, 2853, 1673, 1631, 1594, 1489, 1447, 1408, 1313, 1275, 1212, 1182, 1133; ESI HRMS, calculated for [C22H18O3S+H]^+^: 363.1049 found: 363.1037.

## 4. Conclusions

This paper introduces details of a conceptually new synthetic protocol to assemble the *trans-*(±)-2,3-dihydro-2,3,6-triaryl-1,4-oxathiin, S,S-dioxide ring system. The protocol requires deprotonation of the benzyl position of benzyl alkynyl sulfones employing sub-stoichiometric amounts of strong base. The anion so formed undergoes nucleophilic attack of an added aryl aldehyde and the ensuing alkoxy anion performs an intramolecular conjugate addition on the proximal triple bond of the original starting material. The protocol delivers fair to good yields of 2,3,6 trisubstuted-1,4-oxathiins, many in analytically pure form. The reaction is hindered by breakdown of the products under the reaction conditions, affording a kinetically formed keto sulfone, the presence of which reduces the yield of oxathiin and may hinder purification of the oxathiin product.

The preferred 1,4-oxathiin preparation for applications in pesticide science seems to be ring expansion chemistry of oxathiolanes. More recent work has demonstrated alternative protocols that introduce stereochemistry on the saturated 2-and 3-carbons. Our 1,4-oxathiin preparation complements those existing protocols for oxathiin synthesis in that it represents the only method for the placement of aryl groups at positions 2, 3 and 6. Moreover the relative stereochemistry at positions 2 and 3 is reliably set as trans during the chemistry. The preparation unveils a new library of highly substituted 1,4-oxathiins, which may hold value as bioactive compounds in a number of applications. From a strategy point of view, the methodology represents a previously unknown assembly mode for oxathiins and opens the door for additional variations on this reaction model.

A computational assessment of both oxathiin formation and breakdown reveal eight modes for benzyl anion attack of aryl aldehyde which converge to cis and trans addition products possessing an active alkoxide. The oxyanion can attack the pendant alkyne to create cis- and trans-diaryl forms of the oxathiin ring system, with a clear preference for the trans system, as is obtained practically. The computational work also offers pathways to account for the observed ring-opening decomposition to keto sulfone. If **5_cis_** is formed through cyclization, there appears to be proton transfer reaction that would promote its breakdown over that of the isolated product **5_trans_**.

## Figures and Tables

**Table 1:** Formation of 2,3,6-trisubstitutd 1,4-oxathiins.

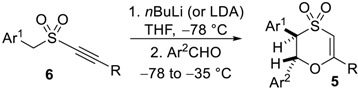				
#			Scale *^a^*	Yield *^b,c^*
1	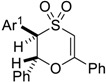	Ar^1^ = C_6_H_5_, **5a**	1	74%
2		C_6_H_5_, **5a**	4	86% *^d^*
3		C_6_H_5_, **5a**	4	75% *^e^*
4		2-I-C_6_H_4_, **5b**	2	78%
5		3-Cl-C_6_H_4_, **5c**	3	76% *^e^*
6		4-NO_2_-C_6_H_4_, **5d**	3	62% *^e^*
7		4-CH_3_-C_6_H_4_, **5e**	1	76%
8		4-CH_3_O-C_6_H_4_, **5f**	1	44%
9		4-Br-C_6_H_4_, **5g**	1	73%
10		3-CN-C_6_H_4_, **5h**	3	64%
11	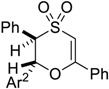	Ar^2^ = 4-CN-C_6_H_4_, **5i**	1	69%
12		4-Br-C_6_H_4_, **5j**	1	34%
13		4-F-C_6_H_4_, **5k**	1	78%
14		4-NO_2_-C_6_H_4_, **5l**	1	62%
15		2-CF_3_-C_6_H_4_, **5m**	2	35%
16		4-CH_3_O-C_6_H_4_, **5n**	2	74%
17		2-furyl, **5o**	2	37%
18	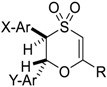	X = 4-Me; Y = 4-NO_2_; R = Ph, **5p**	1	55%
19		X = 2-I; Y = 4-NO_2_; R = Ph, **5q**	1	64% *^d,e^*
20		X = 2-I; Y-Ar = 2-thienyl; R = Ph, **5r**	4	35% *^d,e^*
21		X = 2-I; Y = H; R = *t*Bu, **5s**	3	39% *^d^*
22		X = 2-I; Y = H; R = Me, **5t**	3	38%

*^a^* Moles of starting sulfone substrate. *^b^* Purification was by method A unless otherwise noted. (see Experimental); *^c^ n*BuLi was the base unless otherwise indicated. *^d^* LDA was employed as the base. *^e^* Purified by Method B.

**Table 2:** Isomerization of double-bonded sulfones **12** *^a^*.

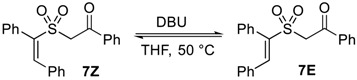		
	Relative Ratio of Isomers *^b^*	
Duration (h)	% 7Z	% 7E
0 *^c^*	76	24
24	26	74
48	3	97
72	0	100

*^a^* As mentioned in the text, although compounds submitted to the conditions are neutral isomers **7Z/E**, anionic forms **12** (Scheme 1) are mostly likely to succumb to base attack and isomerization. *^b^* Measured at each juncture in time. *^c^* 76:24 is the starting ratio.

**Table 3:** Bond lengths and angles for cyclization leading to cis and trans oxathiin formation.

	C5-C6 (Å)	C5-S (Å)	S-O1 (Å)	S-O2 (Å)	S-C3 (Å)	C3-C2 (Å)	C2-Ob (Å)	O1-Li *^a^* (Å)	O2-Li ^a^ (Å)	Ob-Li ^a^ (Å)	Ob-C6 (Å)	PhC_3_-C_2_Ph Dihedral	HC_3_-C_2_H Dihedral
**9_cis_**	1.206	1.717	1.457	1.473	1.819	1.577	1.353	-	1.971	1.783	3.969	55.0	39.2
**9_cis_^‡^**	1.250	1.715	1.460	1.484	1.839	1.574	1.379	-	1.977	1.848	2.152	38.7	23.7
**10_cis_**	1.344	1.732	1.465	1.502	1.845	1.570	1.434	-	1.915	2.152	1.426	10.7	4.7
**9_trans_**	1.206	1.718	1.473	1.456	1.822	1.577	1.351	1.979	-	1.774	3.819	−60.8	176.5
**9_trans_^‡^**	1.250	1.716	1.486	1.459	1.831	1.574	1.376	1.974	-	1.852	2.158	−76.0	161.7
**10_trans_**	1.344	1.731	1.505	1.464	1.856	1.548	1.434	1.900	-	2.287	1.423	−83.3	161.1

^a^ In this table, O1 and O2 represent sulfonyl oxygens; Ob designates the oxathiin ring oxygen.

## Supplementary Materials

The following supporting information can be downloaded at: https://www.mdpi.com/article/10.3390/molecules28176180/s1, seven Figures and eight Tables of addition synthetic and computational information; copies of NMR’s of compounds **5** and **7**; Cartesian coordinates and thermochemistry data of computed structures and transition states (120 pp).

## Sample Availability

Selected samples of the compounds are available from the authors.
