# Supplementary material for: The Synthesis and Base-Induced Breakdown of Triaryl 1,4-Oxathiins—An Experimental and DFT Study
Source: Molecules. 2023 Aug 22;28(17):6180. doi: 10.3390/molecules28176180 (PMC10489040; doi:10.3390/molecules28176180)
Supplement: Supplementary file 1 [file molecules-28-06180-s001.zip › molecules-2564571-supplementary.pdf]

The Synthesis and Base-Induced Breakdown of Triaryl 1,4-Oxathiins. An Experimental and DFT Study.

Eric A. Nicol, Matthew Sing, Lilly U. Luu, Erwin J. Remigio, Michelle B. Mills and Adrian L. Schwan*

Supplementary Materials

Tables relating to cyclization reaction optimization	S2
Description of NOE analysis of compounds **7E/Z**	S5
Figures and Tables and some analysis of computation findings	S6
Characterization data for unreported sulfone starting materials (**6**)	S19
NMR Spectra for compounds **5** and **7**	S20
Cartesian coordinates and thermochemistry data of structures and transition states	S43

Table S1. The effect of time on the production of product, by-product, starting material recovery and overall mass.

Time of reaction	Equivalency of			Mass recovery
	Starting material remaining	Oxathiin-S,S-dioxide (**5a**)[a]	By-product (**7**)	
45 min	2.0	1.0	0	84%
4 hours	0.8	1.0	0.12	70%
6 hours	0.3	1.0	0.26	63%
18 hours	0.1	1.0	0.35	52%
4 days	0	0	0	0%

a Relatvie amont of oxathiin arbitrarily set to 1

Table S2: Optimizations for oxathiin formation

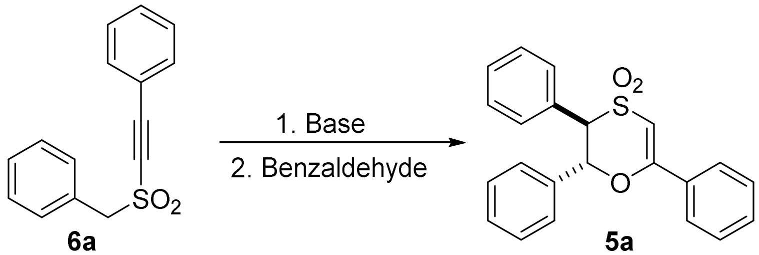

6a 5a

Entry	Base	Equivalents	Aldehyde equivalents	Isolated yield (%)[a]
1	BuLi	1.0	2.0	58
2	BuLi	0.7	2.0	23
3	BuLi	0.6	2.0	50
4	BuLi	0.45	2.0	57
5	BuLi	0.4	2.0	12
6	BuLi	0.25	2.0	19
7	BuLi	0.5	2.0	70
8	LDA	0.5	2.0	68
9	tBuOK	1.2	2.0	38
10	tBuOK	1.2	Xs	~5 (NMR)
12	NaH	1.2	2.0	~5 (NMR)
13	BuLi	0.5	1.5	72-76[b]
14	LDA	0.5	1.5	58

a Isolated yield unless indicated otherwise. *B Multiple trials*

Table S3. Ring openings of oxathiin **5a**

#	Base[a]	conditions	Extent of reaction[b]	Isolated yield	E/Z ratio[c]
1	LiOMe (1M in MeOH)	-35 °C/ 24 h	0%	--	--
2	NaOH (1M in MeOH)	-35 °C/ 24 h	90%	73%	10/90
3	KO*t*Bu	-35 °C/ 24 h	68%	67%	22/78
4	LiOMe (1M in MeOH)	-35 °C/3 h; then rt/21 h	100%	82%	18/82
5	NaOH (1M in MeOH)	-35 °C/3 h; then rt/21 h	94%	75%	8/92
6	KO*t*Bu	-35 °C/3 h; then rt/21 h	100%	87%	12/88
7	LiOMe (1M in MeOH)	rt/24 h	100%	83%	18/82
8	NaOH (1M in MeOH)	rt/24 h	95%	76%	8/92
9	KO*t*Bu	rt/24 h	100%	90%	15/75
10	KOH (1M in MeOH)	rt/24 h	96%	81%	7/93
11	DBU	rt/24 h	100%	96%	17/83[d]

a Reactions were performed in THF; Some bases were introduced via a 1 molar MeOH solution.

b Estimated by inspection of the ^{1}H NMR of the crude reaction mixture.

c Determined by ^{1}H NMR.

d Extended exposure created additional E isomer.

nOe Experiments of Keto-sulfones **7E**/**7Z**

The 3D ^{13}C-NOESYHSQC spectrum was collected on a Bruker AVANCE III 600 MHz spectrometer equipped with a 5 mm TCI cryoprobe. The sample temperature was regulated at 298 ± 1 K. The pulse sequence *noesyhsqcetgp3d* was used as provided by Bruker. The NOESY mixing time was set to 500 ms, the interscan delay was set to 2.5 sec, and 8 scans were collected per increment.

Two approaches were utilized to optimize spectral resolution while minimizing spectrometer time. First: the ^{13}C dimension was collected with a spectral width of 9 ppm (centred at 131 ppm) which resulted in the aliasing of peaks associated with the vinyl C-H and CH_2 groups. The spectral width and central frequency were optimized to ensure the aliased peaks did not interfere with any other peaks. Second: the non-uniform sampling (NUS) feature of the Bruker Topspin acquisition software was used to randomly acquire 1792 increments from a conventional sampling grid of 36 indirect ^{13}C increments and 384 indirect ^{1}H increments (spanning 8.5 ppm), representing a NUS percentage of 13%. The total acquisition time was 12.74 h, as compared to > 98 h for a conventionally acquired spectrum. The spectrum was processed using the SMILE[1] NUS processing plug-in to the NMRPipe[2] processing suite.

Relevant nOe enhancements of **7E**:

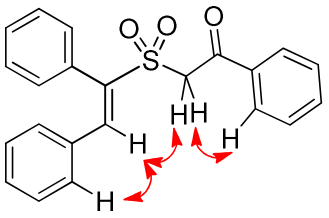

Relevant nOe enhancements of **7Z**:

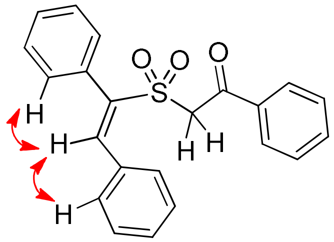

[1] J. Ying, F. Delaglio, D.A. Torchia, and A. Bax: Sparse Multidimensional Iterative Lineshape-Enhanced (SMILE) Reconstruction of Both Non-Uniformly Sampled and Conventional NMR Data. J Biomol NMR 68, 101-118 (2017).

[2] Delaglio F, Grzesiek S, Vuister GW, Zhu G, Pfeifer J, Bax A: NMRpipe—a multidimensional spectral processing system based on UNIX pipes. J Biomol NMR 6:277–293 (1995).

Figure S1. Optimized geometries of pre-complexes for addition of benzaldehyde to lithiated sulfone **8**.

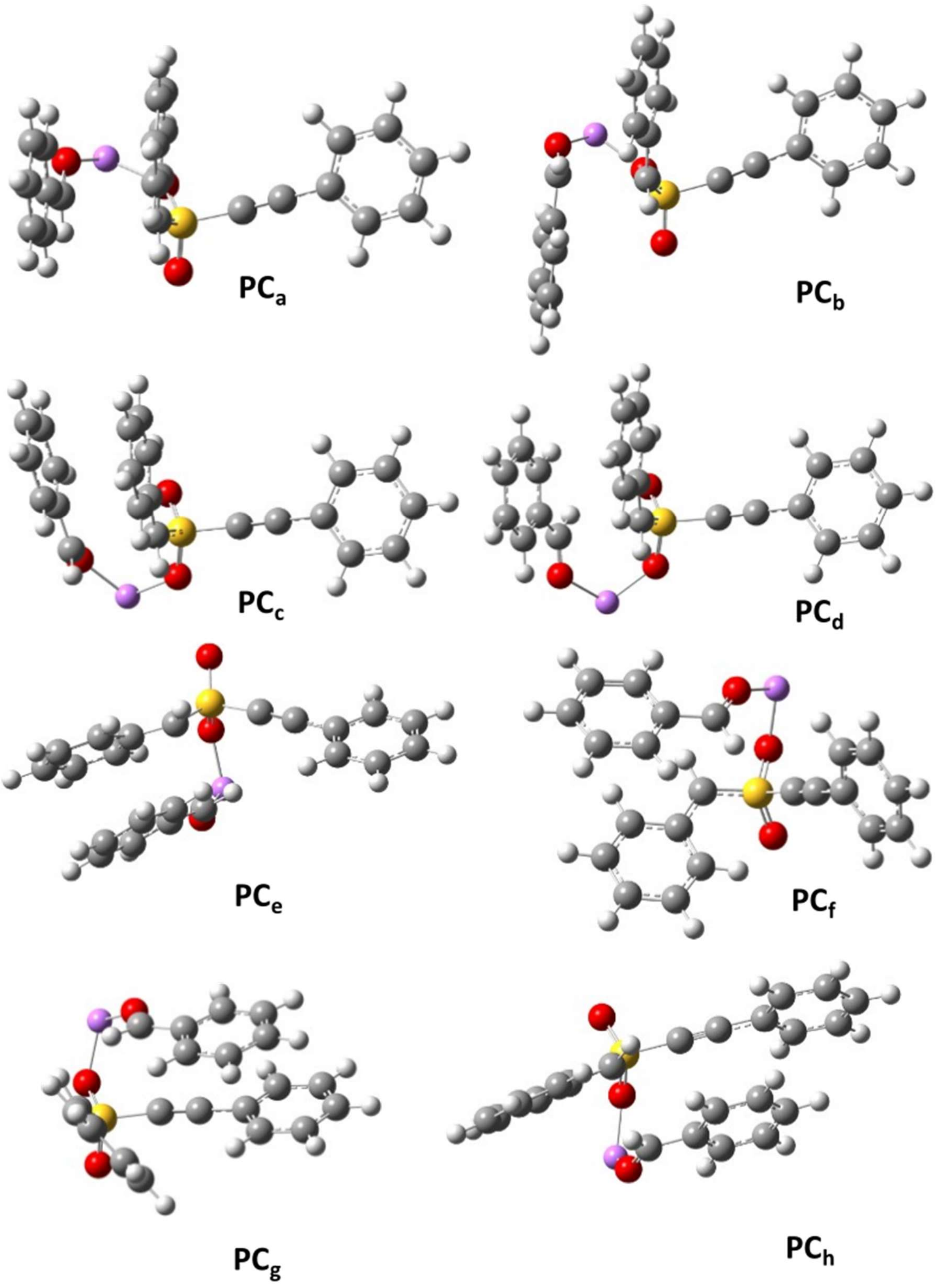

Figure S2: Optimized transition state structures for addition of benzaldehyde to lithiated sulfone **8**.

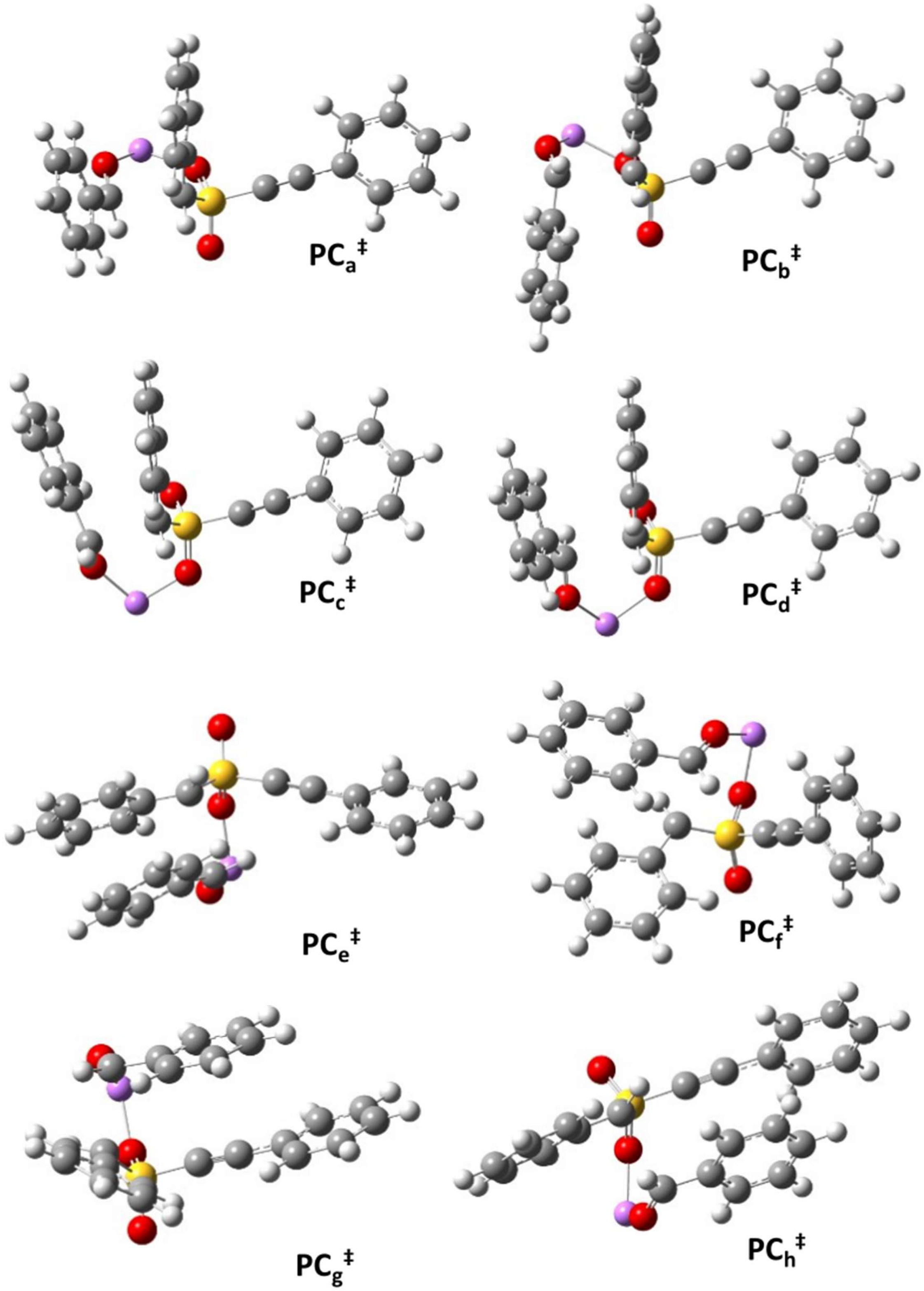

Figure S3. Optimized geometries of benzaldehyde addition products $\mathbf{9_{a\text{-}h}}$

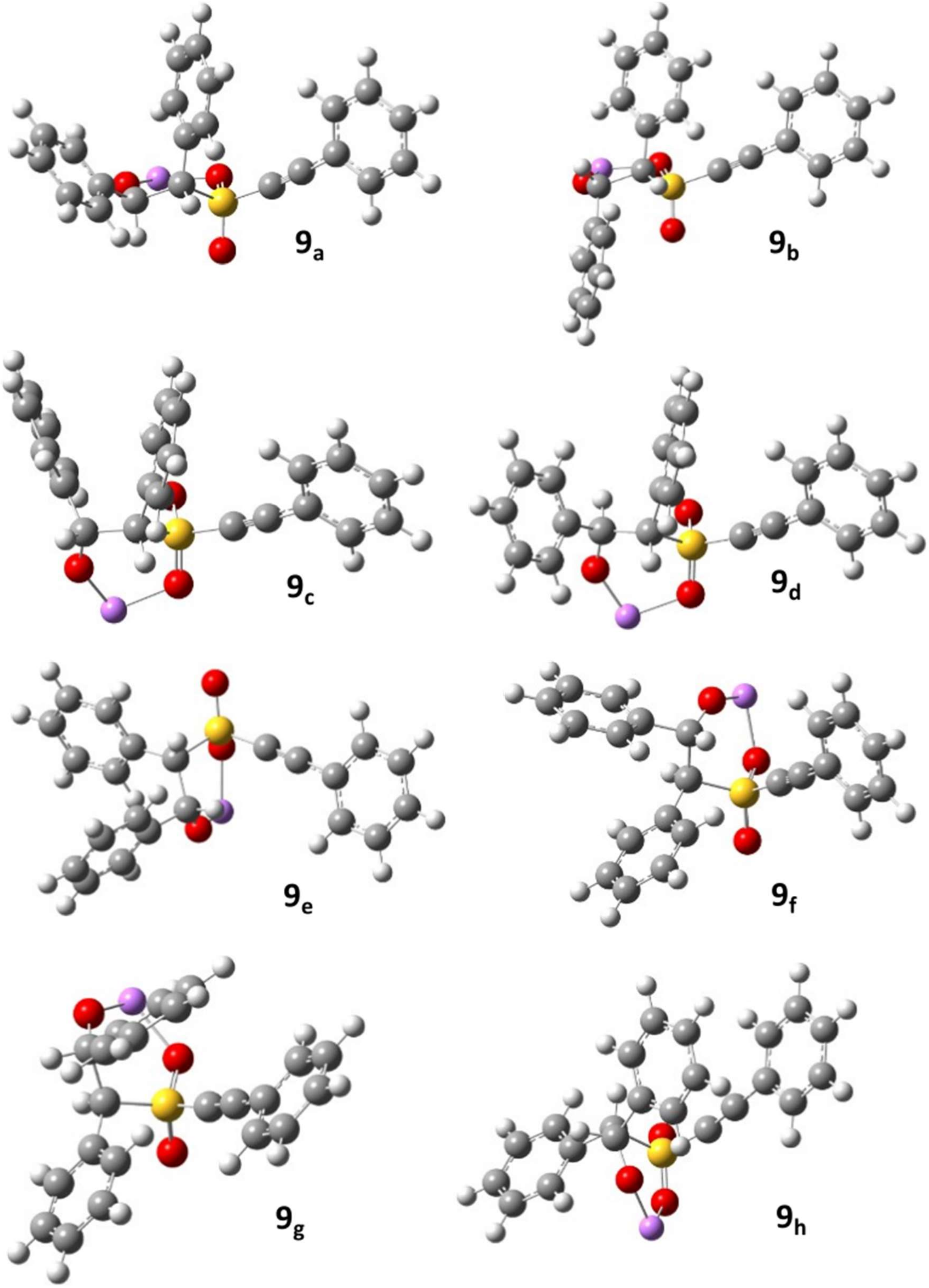

Figure S4. Energy profile for benzaldehyde addition to lithiated benzyl alkynyl sulfones. Reactions originate in the center and proceed left for cis cyclizations and right for trans cyclizations.

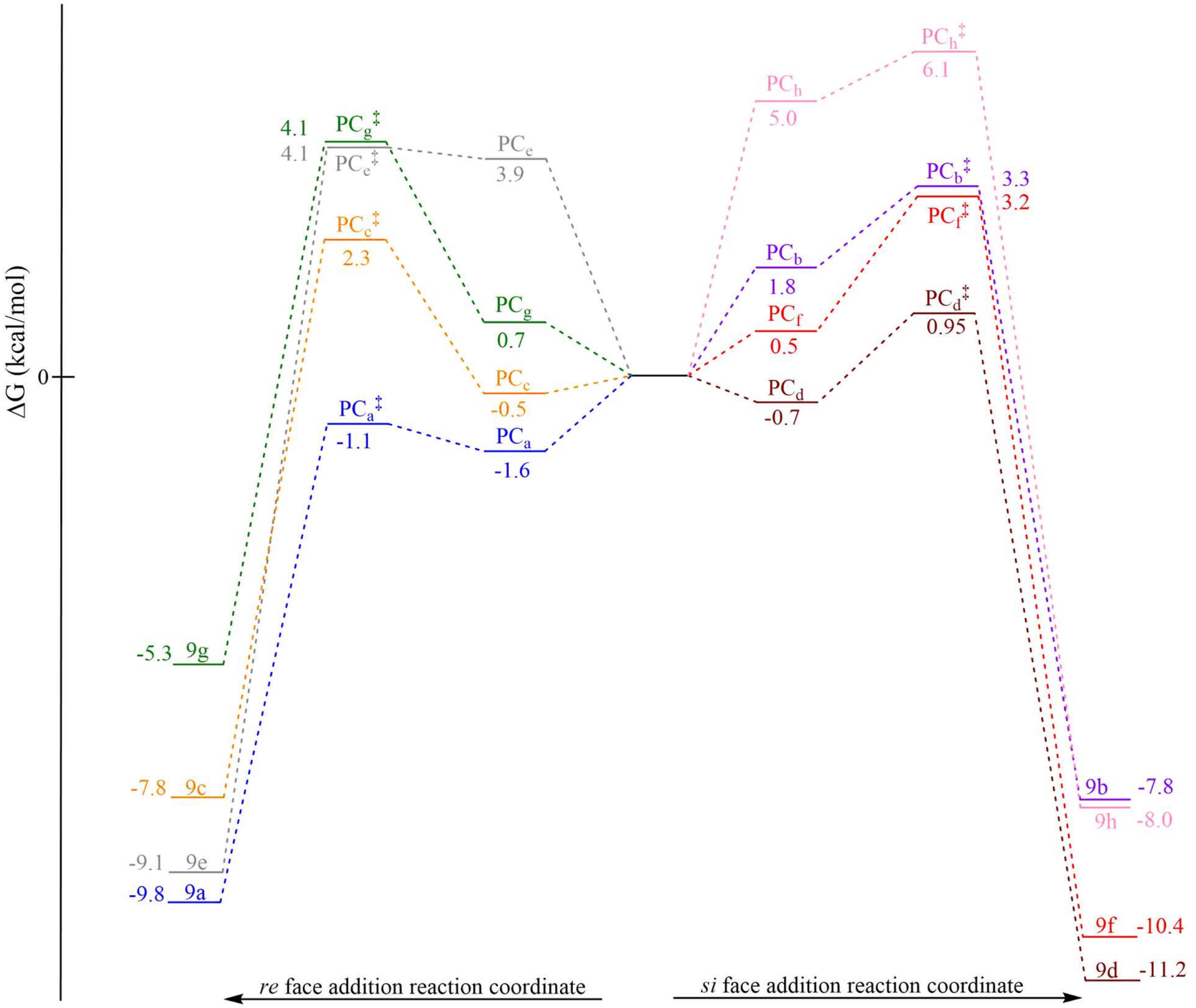

Table S4. Selected computed parameters for addition of benzaldehyde to lithiated sulfone **8.**

	C_5-C_6 (Å)	C_5-S (Å)	S-O_1 (Å)	S-O_2 (Å)	S-C_3 (Å)	C_2-C_3 (Å)	C_2-O_b (Å)	O_1-Li (Å)	O_2-Li (Å)	O_b-Li (Å)	PhC_3-C_2Ph dihedral (°)	HC_3-C_2H dihedral (°)
**PC_a**	1.209	1.754	1.490	1.467	1.661	2.876	1.223	1.875	-	1.918	-52.1	-48.6
**PC_b**	1.209	1.754	1.490	1.464	1.659	2.801	1.222	1.882	-	1.921	-172.3	64.3
**PC_c**	1.209	1.757	1.467	1.490	1.657	2.856	1.225	-	1.901	1.932	15.7	22.2
**PC_d**	1.209	1.754	1.469	1.490	1.654	2.826	1.226	-	1.889	1.948	-27.1	-150.5
**PC_e**	1.207	1.738	1.471	1.491	1.673	2.755	1.226	-	1.898	1.921	-53.3	-49.0
**PC_f**	1.209	1.758	1.491	1.467	1.651	2.889	1.222	1.916	-	1.937	-56.9	177.7
**PC_g**	1.209	1.754	1.493	1.467	1.656	3.165	1.220	1.916	-	1.937	35.6	59.9
**PC_h**	1.206	1.729	1.473	1.495	1.679	2.706	1.225	-	1.883	1.908	-170.0	65.9
**$PC_a^‡$**	1.208	1.743	1.486	1.464	1.683	2.419	1.239	1.903	-	1.885	-62.2	-55.7
**$PC_b^‡$**	1.208	1.745	1.486	1.461	1.684	2.407	1.237	1.904	-	1.876	-169.8	65.5
**$PC_c^‡$**	1.208	1.741	1.462	1.484	1.696	2.311	1.248	-	1.920	1.864	24.7	32.8
**$PC_d^‡$**	1.209	1.743	1.465	1.485	1.683	2.394	1.242	-	1.921	1.893	-37.3	-161.5
**$PC_e^‡$**	1.207	1.736	1.470	1.489	1.684	2.573	1.232	-	1.902	1.908	-54.1	-48.7
**$PC_f^‡$**	1.207	1.741	1.487	1.465	1.684	2.449	1.238	1.933	-	1.880	-56.5	179.1
**$PC_g^‡$**	1.206	1.730	1.488	1.467	1.690	2.399	1.241	1.905	-	1.877	64.9	69.3
**$PC_h^‡$**	1.206	1.728	1.472	1.493	1.687	2.569	1.230	-	1.890	1.895	-169.1	66.5
**9_a**	1.206	1.718	1.473	1.457	1.816	1.585	1.351	1.994	-	1.785	-56.8	-42.2
**9_b**	1.206	1.721	1.473	1.455	1.824	1.584	1.352	1.988	-	1.772	-150.3	86.6
**9_c**	1.206	1.722	1.456	1.474	1.823	1.590	1.350	-	1.983	1.771	43.6	55.6
**9_d**	1.206	1.720	1.458	1.474	1.823	1.575	1.353	-	1.991	1.777	-59.8	179.2
**9_e**	1.206	1.717	1.457	1.473	1.819	1.577	1.353	-	1.971	1.783	-55.0	-39.2
**9_f**	1.206	1.718	1.473	1.456	1.823	1.577	1.351	1.978	-	1.775	-61.0	176.4
**9_g**	1.205	1.715	1.472	1.457	1.815	1.592	1.352	1.964	-	1.780	68.5	76.4
**9_h**	1.206	1.711	1.458	1.472	1.825	1.576	1.353	-	1.963	1.772	-150.9	84.7

Brief Discussion of Trends exhibited in Table S4.

Pre-complexes contain similar bond lengths throughout the structures aside from C_2-C_3 distances, which vary from 2.706 Å in **PC_h** to 3.165 Å in **PC_g**. Regarding geometries for transition states of benzaldehyde addition, notable changes in bond length occur between C_2 and C_3 as formation of the bond progresses. The most significant change is observed in **$PC_g^‡$** where the distance shortens by 0.766 Å, while the least significant change occurs in **$PC_h^‡$** where the separation decreases by 0.137 Å. These systems have the highest energy transition states of their respective pathways, either due to the higher energy of the initial pre-complex (**PC_h**), or as a result of larger activation energies (**PC_g**). In conjunction with C_2-C_3 bond formation, a slight lengthening of both the S-C_3 and C_2-O_b bonds arises as the O_b-Li distance decreases.

Transition states for benzaldehyde addition closely resemble the pre-complexes as evidenced by the low activation barriers and bond length data. Aside from the **PC_g/9_g** system, >60% of the closure of the C_2-C_3 interatomic distances occurs from transition state to product. The addition products **9** exhibit a slightly longer than average C-C bond length of 1.575-1.592 Å, in addition to elongated C_3-S (~0.16 Å) and C_2-O_b (~0.13 Å) bond distances. As the reaction progresses and the negative charge is transferred to O_b, there is an average decrease in the O_b-Li distance by 0.15 Å. The O_1 and O_2 to Li distances show minor increases as the counterion moves closer to the O_b oxyanion with elongations ranging from 0.05 – 0.12 Å.

Table S5. Bond lengths and angles for selected benzaldehyde addition products and pre-cyclization structures.

	C_5-C_6 (Å)	C_5-S (Å)	S-O_1 (Å)	S-O_2 (Å)	S-C_3 (Å)	C_3-C_2 (Å)	C_2-O_b (Å)	O_1-Li (Å)	O_2-Li (Å)	O_b-Li (Å)	PhC_3-C_2Ph dihedral (°)	HC_3-C_2H dihedral (°)
**9_g**	1.205	1.715	1.472	1.457	1.815	1.592	1.352	1.964	-	1.780	68.5	76.4
**9_e /9_{cis}**	1.206	1.717	1.457	1.473	1.819	1.577	1.353	-	1.971	1.783	-55.0	-39.2
**9_f**	1.206	1.718	1.473	1.456	1.823	1.577	1.351	1.978	-	1.775	-61.0	176.4
**9_h**	1.206	1.711	1.458	1.472	1.825	1.576	1.353	-	1.963	1.772	-150.9	84.7
**9_{trans}**	1.206	1.718	1.473	1.456	1.822	1.577	1.351	1.979	-	1.774	-60.8	176.5

Figure S5. Energy profiles of ring opening of lithiated oxathiins.

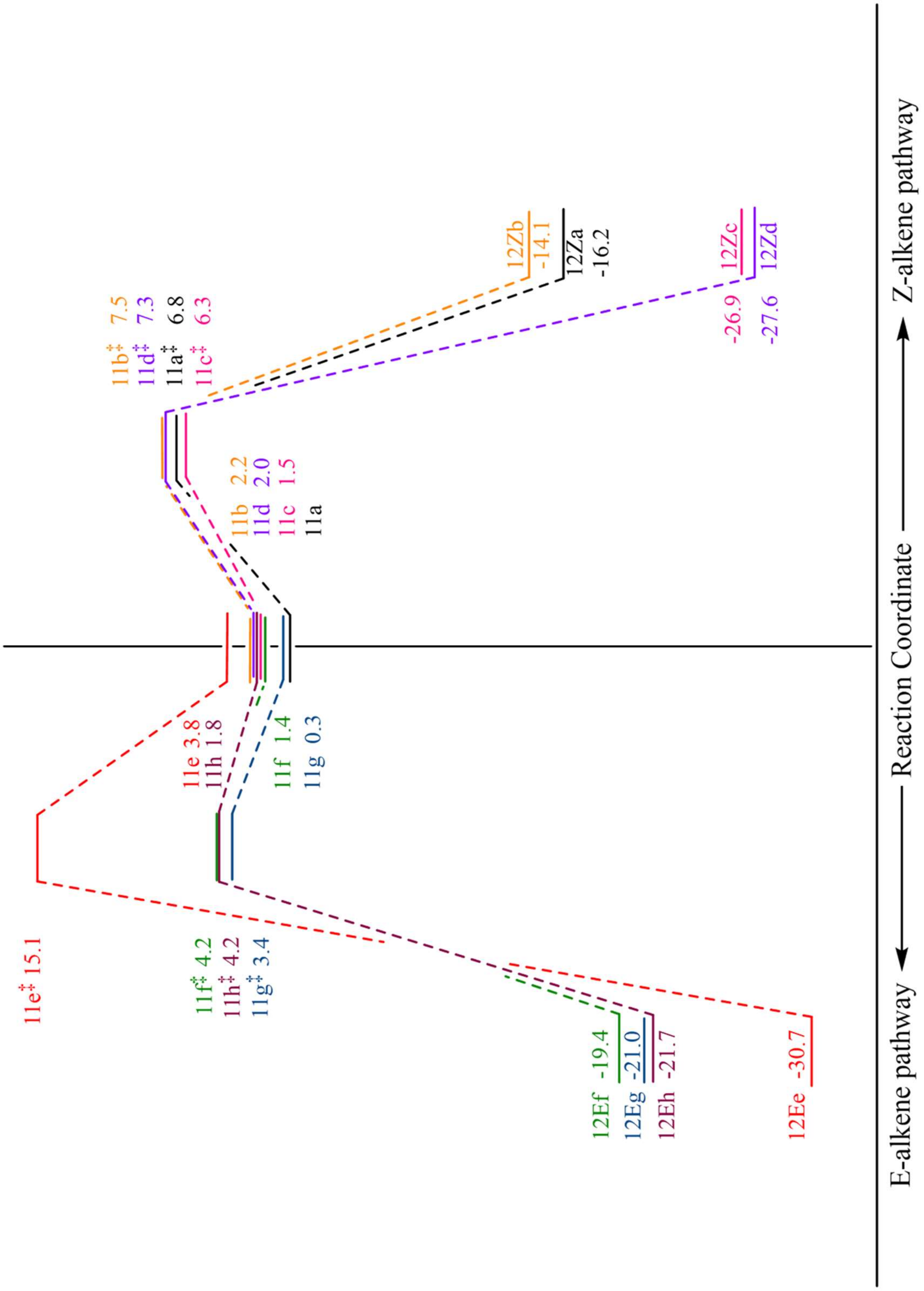

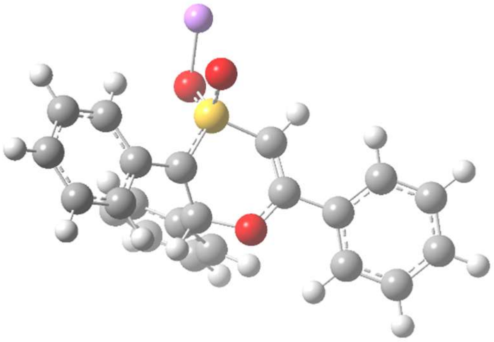

11a: r(Li-O) = 1.989, 2.006 Å

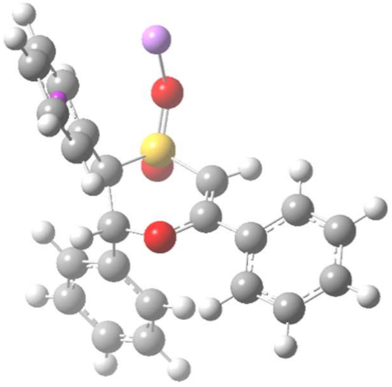

11b: r(Li-O): 1.847 Å; r(Li to 3-Ar) = 3.257 Å

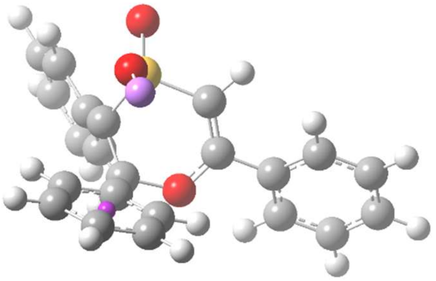

11c: r(Li-O): 1.831 Å; r(Li to 2-Ar) = 3.119 Å

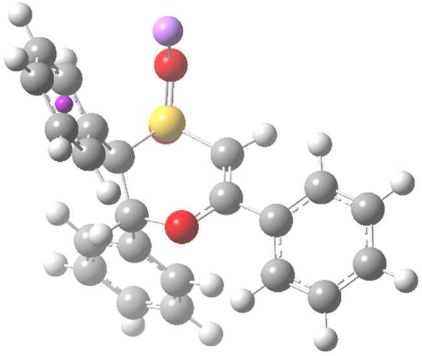

11d: r(Li-O): 1.857 Å; r(Li to 3-Ar) = 3.257 Å

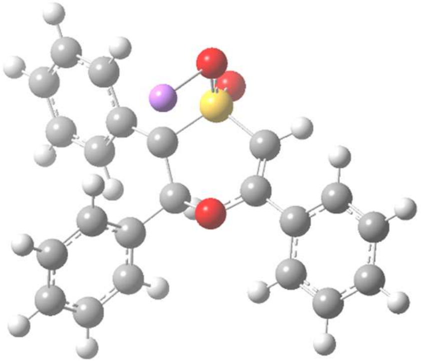

11e: r(Li-O): 1.981 Å; r(Li-C): 2.339 Å

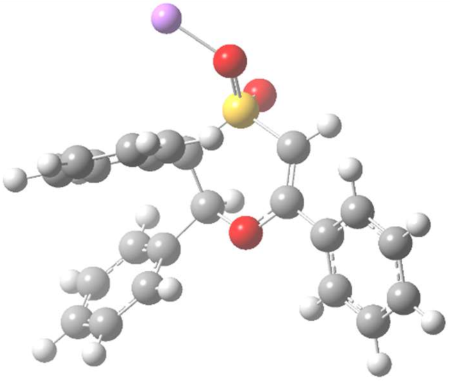

11f: r(Li-O): 1.869 Å; r(Li-C): 3.462 Å

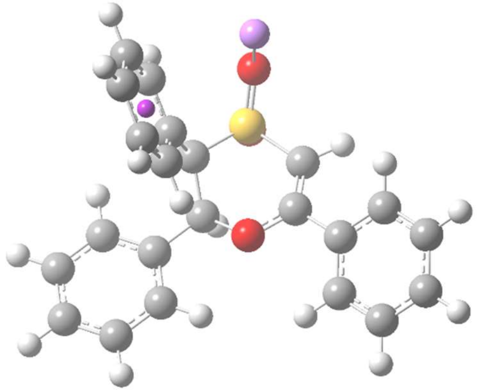	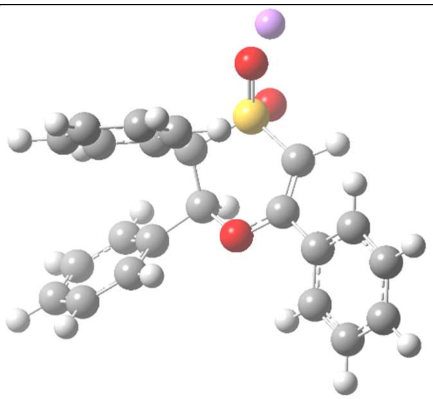
**11d**: r(Li-O): 1.868 Å; r(Li to 3-Ar) = 3.377 Å	**11h**: r(Li-O) = 2.021, 2.071 Å

Figure S6 Optimized oxathiins 11 monolithated at carbon-3.

Comments about lithiated ring opening precursors 11 (Figure S6) :

1. Only structures **11a -11d** underwent ring opening to the kinetic product.
2. Structure **11b** and **11d** are very similar in geometry and energy.
3. Structures **11b**, **11c** and **11d** exhibit a strong Li to Ar interaction. Structure **11c** engages the Li with the Ph group on the 2-carbon of the ring, whereas structures **11b** and **11d** demonstrate a Li interaction with the Ph group on the 3-carbon of the ring.
4. Only structures **11a** and **11h** exhibit the lithium bridging between the two sulfonyl oxygens.
5. All eight of the structures (Figure S6) exhibit at least one strong sulfonyl O to Li affiliation (1.831 to 2.021 Å)
6. Structure **11e** holds the Li closest to the 'deprotonated' carbon (2.339 Å)

starting anion	Transition state	Ring-opened byproduct
**11_a** →	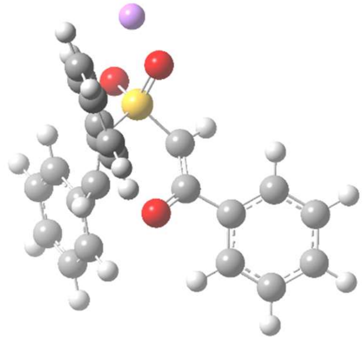<br>**11_aTS**: r(Li-O) = 2.014, 2.038 Å	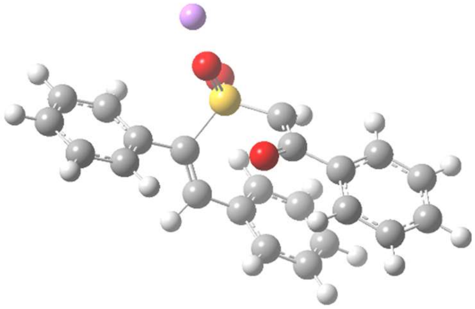<br>**$12Z_a$**: r(Li-O) = 1.858, 2.077 Å
**11_b** →	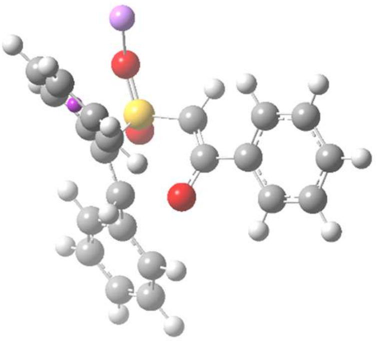<br>**11_b**: r(Li-O): 1.855 Å; r(Li to 3-Ar) = 3.503 Å	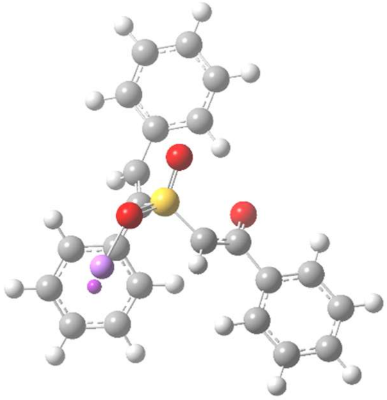<br>**$12Z_b$**: r(Li-O): 1.855 Å; r(Li to 3-Ar) = 3.503 Å
**11_c** →	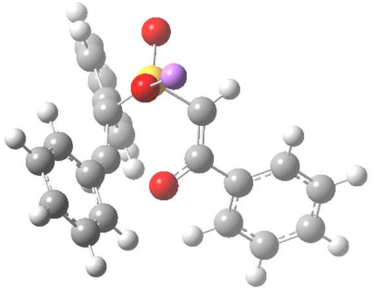<br>**11_c**: r(Li-O): 1.845 Å; r(L to 2-Ar) = 4.395 Å	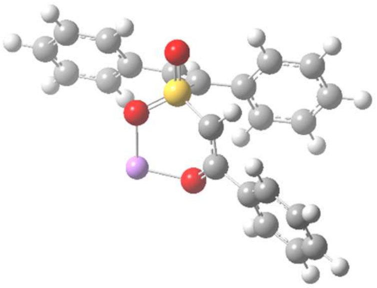<br>**$12Z_c$**: r(Li-OS) = 1.925 Å; r(Li-OC) = 1.883 Å
**11_d** →	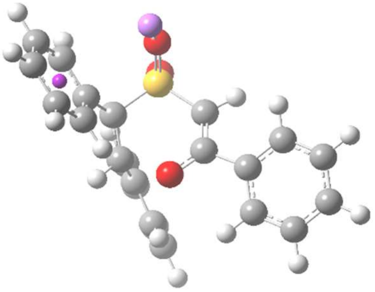<br>**11_d**: r(Li-O): 1.852 Å; r(Li to 3-Ar) = 3.202 Å	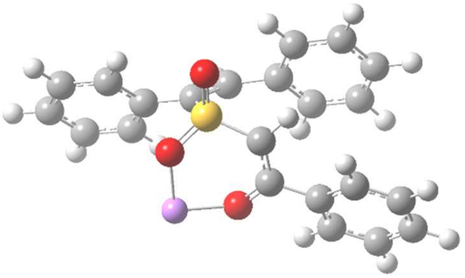<br>**$12Z_d$**: r(Li-OS) = 1.926 Å; r(Li-OC) = 1.880 Å

Figure S7 Optimized structures through ring opening for lithated substrates **11_{a-d}**.

Table S6. Bond lengths and angles of optimized geometries for the oxathiin ring opening process.

	C5-C6 (Å)	C5-S (Å)	S-O1 (Å)	S-O2 (Å)	S-C3 (Å)	C3-C2 (Å)	C2-Ob (Å)	O1-Li (Å)	O2-Li (Å)	Ob-Li (Å)	Ob-C6 (Å)	PhC3-C2Ph dihedral	Li-Ph centroid (Å)
**11a**	1.348	1.745	1.508	1.517	1.663	1.497	1.450	2.005	1.989	-	1.339	100.4	
**11b**	1.349	1.757	1.499	1.471	1.698	1.490	1.472	1.847	-	1.847	1.340	152.8	
**11c**	1.350	1.757	1.497	1.471	1.697	1.491	1.474	1.831	-	4.768	1.340	-155.7	
**11d**	1.349	1.757	1.500	1.471	1.698	1.489	1.473	1.857	-	4.854	1.340	153.4	
**11e**	1.343	1.750	1.508	1.476	1.731	1.516	1.458	1.981	-	3.477	1.341	-55.6	
**11f**	1.352	1.753	1.498	1.475	1.699	1.498	1.487	1.869	-	-	1.338	55.1	
**11g**	1.351	1.761	1.497	1.472	1.700	1.487	1.483	1.868	-	5.007	1.338	53.7	
**11h**	1.355	1.747	1.495	1.501	1.688	1.496	1.486	2.071	2.021	-	1.334	54.5	
**11a‡**	1.384	1.715	1.501	1.503	1.731	1.394	1.890	2.038	2.014	-	1.282	158.5	
**11b‡**	1.373	1.733	1.497	1.468	1.734	1.406	1.838	1.855	-	5.418	1.294	160.4	
**11c‡**	1.373	1.736	1.492	1.470	1.728	1.414	1.799	1.845	-	4.730	1.299	-158.1	
**11d‡**	1.378	1.730	1.501	1.472	1.744	1.394	1.889	1.852	-	4.757	1.288	160.7	
**11e‡**	1.349	1.764	1.513	1.470	1.718	1.443	1.733	1.940	-	2.113	1.347	-8.8	
**11f‡**	1.372	1.730	1.497	1.475	1.725	1.411	1.790	1.866	-	-	1.300	33.5	
**11g‡**	1.374	1.735	1.497	1.471	1.732	1.405	1.820	1.874	-	5.198	1.296	42.1	
**11h‡**	1.378	1.720	1.494	1.502	1.717	1.409	1.791	2.078	2.020	-	1.293	33.4	
**12Za**	1.412	1.683	1.491	1.498	1.814	1.335	3.335	2.076	2.066	-	1.243	178.3	
**12Zb**	1.402	1.698	1.499	1.463	1.808	1.337	3.623	1.858	-	-	1.248	-179.1	
**12Zc**	1.389	1.707	1.487	1.469	1.819	1.337	3.962	1.925	-	1.883	1.269	-176.2	
**12Zd**	1.390	1.702	1.490	1.468	1.822	1.336	3.677	1.926	-	1.879	1.268	178.8	
**12Ee**	1.392	1.704	1.491	1.467	1.807	1.336	3.654	1.913	-	1.862	1.265	-5.1	
**12Ef**	1.406	1.695	1.496	1.468	1.804	1.336	3.251	1.853	-	-	1.245	-4.1	
**12Eg**	1.405	1.698	1.498	1.467	1.801	1.335	3.317	1.855	-	-	1.246	4.2	
**12Eh**	1.408	1.685	1.496	1.494	1.799	1.335	3.461	2.093	2.035	-	1.245	6.4	

Table S7. Reaction free energies for proton transfers between possible anionic intermediates.

reactants	products	ΔG_r
**10**$_{trans'}$ + **6**	**5**$_{trans}$ + **8c**	-1.7
**10**$_{trans'}$ + **5**$_{trans}$	**5**$_{trans}$ + **11c**	2.7
**10**$_{trans'}$ + **5**$_{cis}$	**5**$_{trans}$ + **11a**	-3.3
**10**$_{trans'}$ + **5**$_{cis}$	**5**$_{trans}$ + **11b**	-1.1
**10**$_{trans'}$ + **5**$_{cis}$	**5**$_{trans}$ + **11d**	-1.3
**10**$_{cis'}$ + **6**	**5**$_{cis}$ + **8c**	2.8
**10**$_{cis'}$ + **5**$_{trans}$	**5**$_{cis}$ + **11c**	7.3
**10**$_{cis'}$ + **5**$_{cis}$	**5**$_{cis}$ + **11b**	3.4
**10**$_{cis'}$ + **5**$_{cis}$	**5**$_{cis}$ + **11d**	3.3
**10**$_{cis'}$ + **5**$_{cis}$	**5**$_{cis}$ + **11a**	1.2
**10**$_{trans}$ + **6**	**5**$_{trans}$ + **8c**	-11.0
**10**$_{trans}$ + **5**$_{trans}$	**5**$_{trans}$ + **11c**	-6.6
**10**$_{trans}$ + **5**$_{cis}$	**5**$_{trans}$ + **11a**	-12.6
**10**$_{trans}$ + **5**$_{cis}$	**5**$_{trans}$ + **11b**	-10.4
**10**$_{trans}$ + **5**$_{cis}$	**5**$_{trans}$ + **11d**	-10.6
**10**$_{cis}$ + **6**	**5cis** + **8c**	-9.6
**10**$_{cis}$ + **5**$_{trans}$	**5cis** + **11c**	-5.2
**10**$_{cis}$ + **5**$_{cis}$	**5cis** + **11a**	-11.2
**10**$_{cis}$ + **5**$_{cis}$	**5cis** + **11b**	-9.0
**10**$_{cis}$ + **5**$_{cis}$	**5cis** + **11d**	-9.2
**5**$_{cis}$ + **8c**	**6** + **11a**	-1.6
**5**$_{trans}$ + **8c**	**6** + **11c**	4.4
**5cis** + **8c**	**6** + **11d**	0.4
**5cis** + **8c**	**6** + **11b**	0.6
**8a** + **5**$_{trans}$	**6** + **11c**	4.2
**8a** + **5**$_{cis}$	**6** + **11a**	-1.8
**8a** + **5**$_{cis}$	**6** + **11b**	0.4
**8a** + **5**$_{cis}$	**6** + **11d**	0.2
**8b** + **5**$_{trans}$	**6** + **11c**	3.6
**8b** + **5**$_{cis}$	**6** + **11a**	-2.4
**8b** + **5**$_{cis}$	**6** + **11b**	-0.2
**8b** + **5**$_{cis}$	**6** + **11d**	-0.4

Characterization data for unreported sulfone starting materials (6)

4-Bromobenzyl 2-phenylethynyl sulfone was obtained as a white solid. Yield: 44%. Mp: 157 – 158 °C. ^{1}H NMR (400 MHz, $CDCl_3$): δ 7.55 (m, 5H, Ar H), 7.50 (m, 2H, Ar H), 7.38 (m, 2H, Ar H), 4.48 (s, 2H, CH_2). ^{13}C NMR (100.6 MHz, $CDCl_3$): δ 132.87, 132.80, 132.18, 131.95, 128.88, 126.23, 124.06, 117.28, 94.49, 82.37, 63.89. IR (cm^{-1}, neat): 2988, 2181, 1487, 1328, 1149, 1126, 887, 750. Analysis calculated for $C_{15}H_{11}BrO_2S$; C, 53.74; H, 3.31; found C, 53.60; H, 3.50.

4-Methylbenzyl 2-phenylethynyl sulfone was obtained as a white solid. Yield: 90%. Mp: 75-76 °C. ^{1}H NMR (400 MHz, $CDCl_3$): δ 7.50 (m, 3H, Ar H), 7.41 (m, 4H, Ar H), 7.25 (m, 2H, Ar H), 4.49 (s, 2H, CH_2), 2.41 (s, 3H, CH_3). ^{13}C NMR (100.6 MHz, $CDCl_3$): δ 132.78, 131.73, 131.21, 129.42, 128.87, 128.62, 127.42, 117.46, 94.16, 82.54, 64.62, 21.21; IR (cm^{-1}, neat): 3061, 2979, 2921, 2183, 1512, 1488, 1443, 1199, 1149, 1126, 759. Analysis calculated for $C_{16}H_{14}O_2S$; C, 71.08; H, 5.22; found C, 71.23; H, 5.40.

4-Methoxybenzyl 2-phenylethynyl sulfone was obtained as a white solid. Yield: 65%. Mp: 74 – 75 °C. ^{1}H NMR (400 MHz, $CDCl_3$): δ 7.51 (m, 3H, Ar H), 7.43 (m, 4H, Ar H), 6.97 (m, 2H, Ar H), 4.48 (s, 2H, CH_2), 3.85 (s, 3H, CH_3). ^{13}C NMR (100.6 MHz, $CDCl_3$): δ 160.54, 132.82, 132.53, 131.72, 128.79, 118.96, 117.60, 114.37, 94.04, 82.67, 64.00, 55.38. IR (cm^{-1}, neat): 3001, 2967, 2838, 2182, 1610, 1328, 1305, 1253, 1127. Analysis calculated for $C_{16}H_{14}O_3S$; C, 67.11; H, 4.93; found C, 66.89; H, 5.24.

3-Chlorobenzyl 2-phenylethynyl sulfone was obtained as a white solid. Yield: 78%. Mp: 84 – 85 °C. ^{1}H NMR (400 MHz, $CDCl_3$): δ 7.51 (m, 4H, Ar H), 7.46 (m, 5H, Ar H), 4.49 (s, 2H, CH_2). ^{13}C NMR (100.6 MHz, $CDCl_3$): δ 134.73, 132.88, 131.94, 131.28, 130.20, 129.71, 129.47, 129.18, 128.86, 117.24, 94.72, 82.27, 63.95. IR (cm^{-1}, neat): 3064, 2983, 2924, 2182, 1330, 1256, 1151, 1127. ESI HRMS, calculated for $[C_{15}H_{11}{}^{35}ClO_2S+NH_4]^+$: 308.0507; found: 308.0496.

4-Nitrobenzyl 2-phenylethynyl sulfone (82h) was obtained as a white solid. Yield: 64%. Mp: 169 – 170 °C. ^{1}H NMR (400 MHz, $CDCl_3$): δ 7.53 (m, 9H, ArH), 4.49 (s, 2H, CH_2). ^{13}C NMR (100.6 MHz, $CDCl_3$): δ 134.15, 132.91, 131.94, 130.49, 129.93, 128.86, 122.75, 117.22, 94.78, 82.24, 63.89. IR (cm^{-1}, neat): 3077, 3061, 2932, 2181, 1522, 1490, 1348, 1332, 1150, 1127. Analysis calculated for $C_{15}H_{11}NO_4S$; C, 59.79; H, 3.68; found C, 59.55; H, 3.71.

NMR Spectra for Compounds 5 and 7.

NMR spectra for (±)-**5a**:

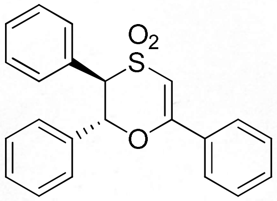

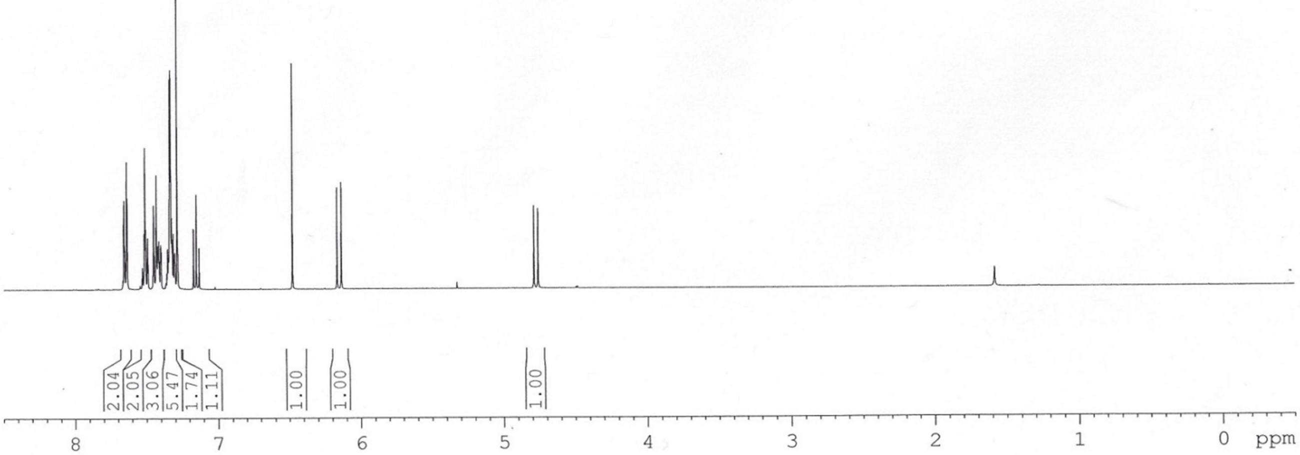

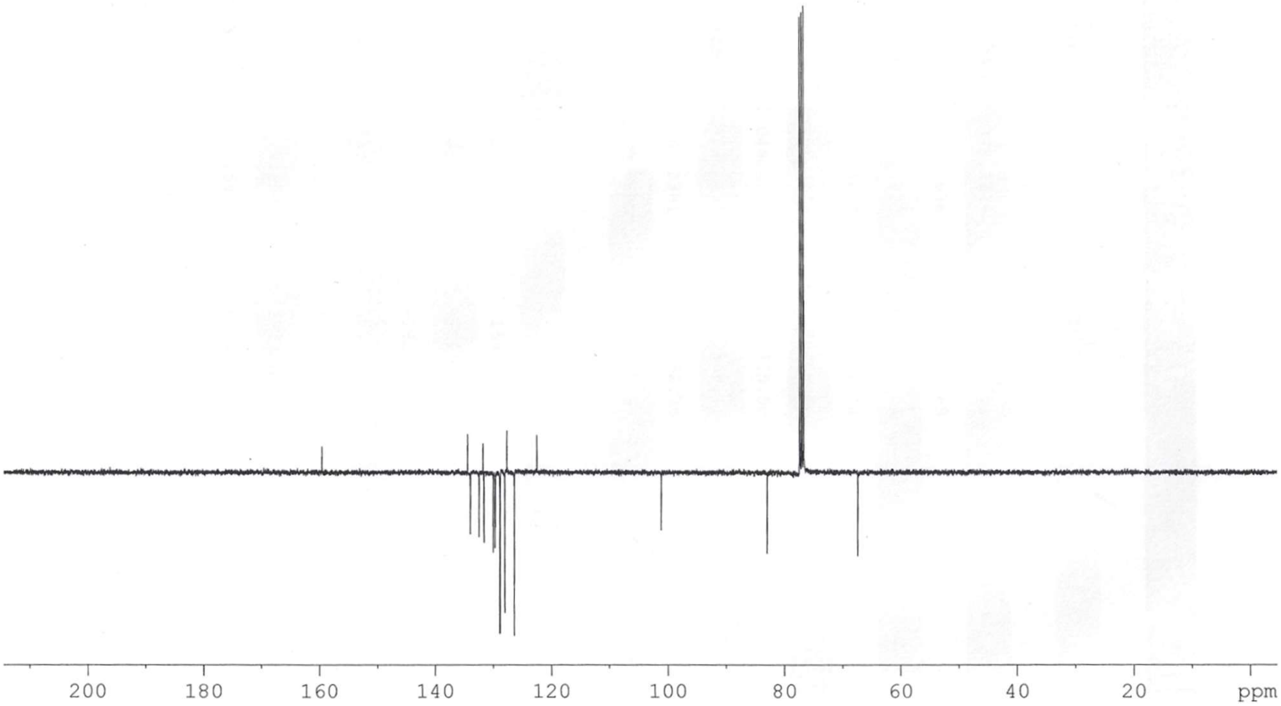

^{1}H and ^{13}C NMR spectra for (±)-**5b**:

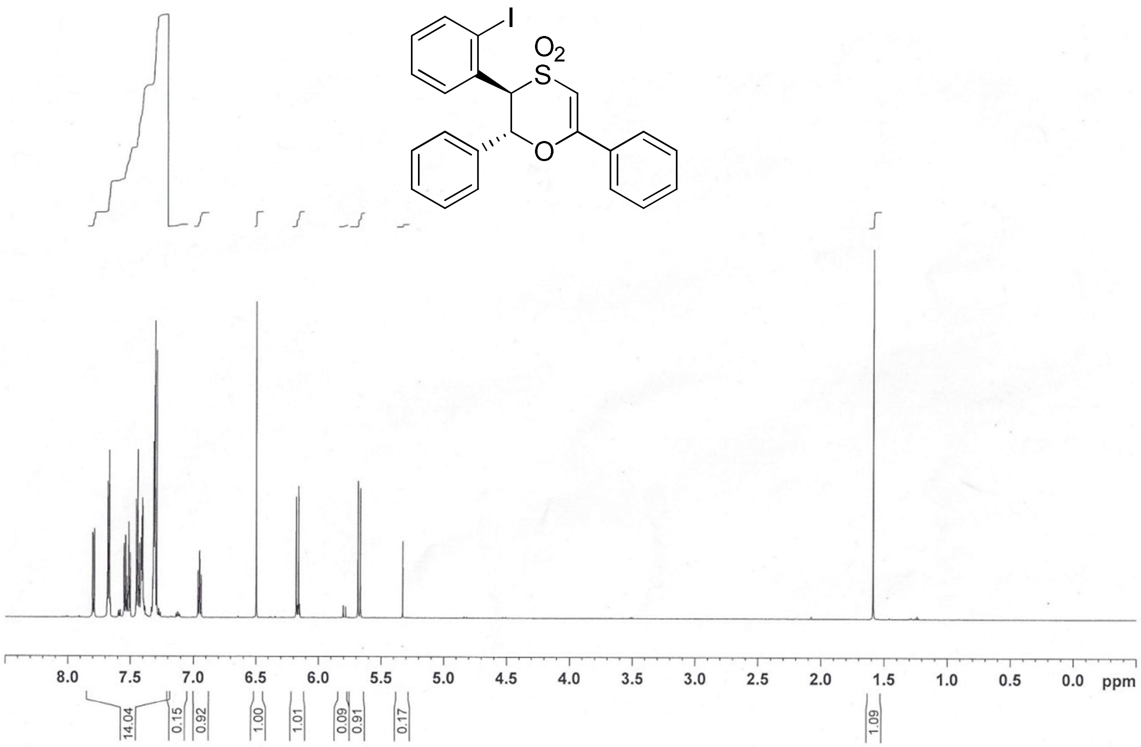

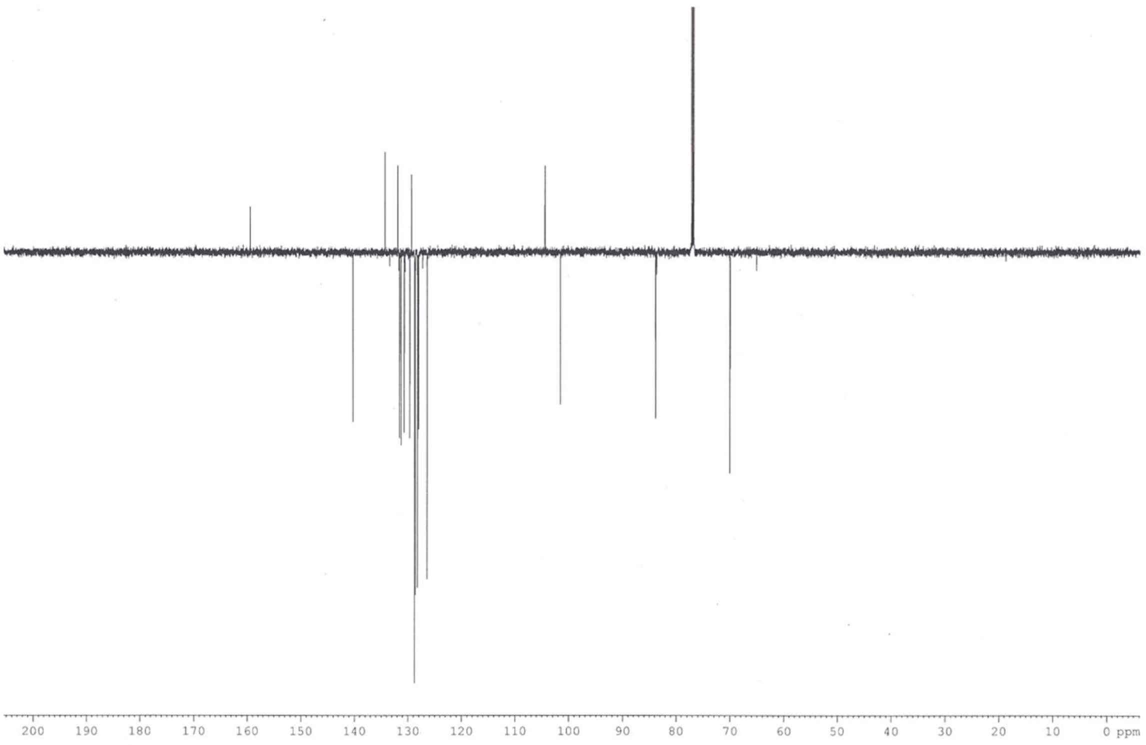

^{1}H and ^{13}C NMR spectra for (±)-**5c**:

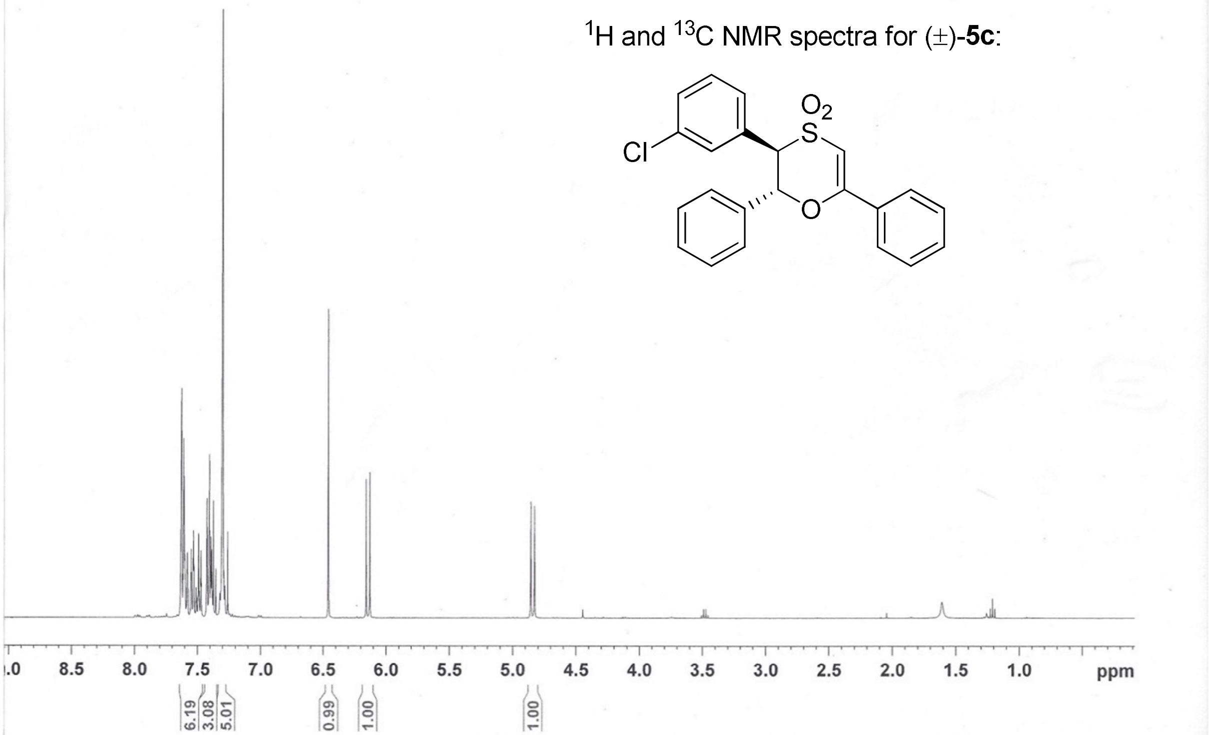

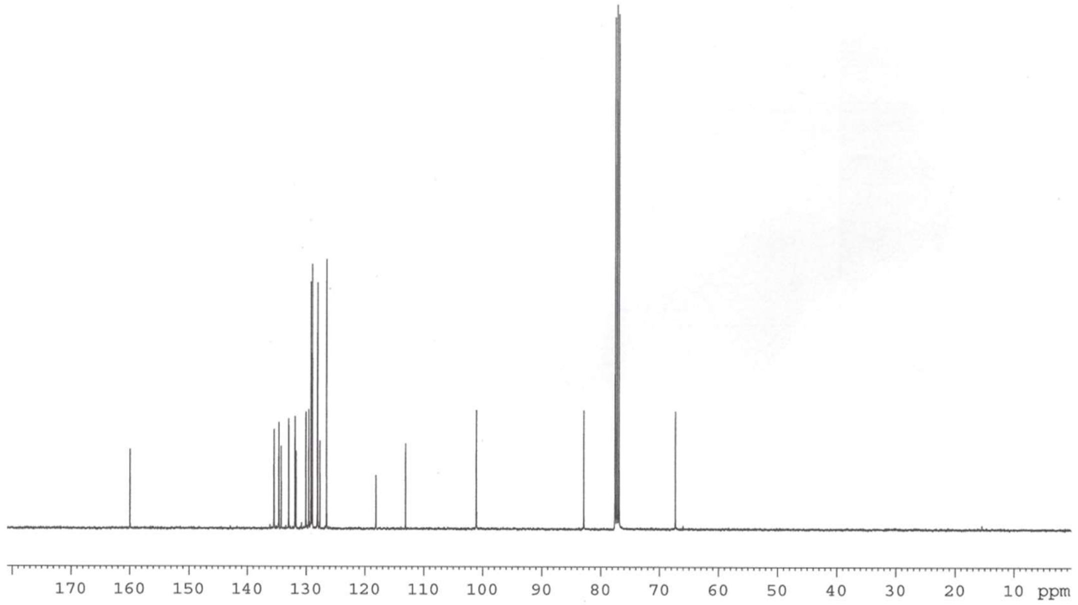

^{1}H and ^{13}C NMR spectra for (±)-**5d**:

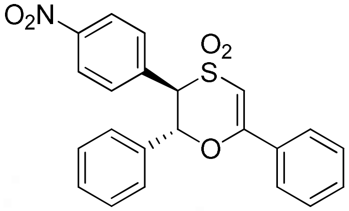

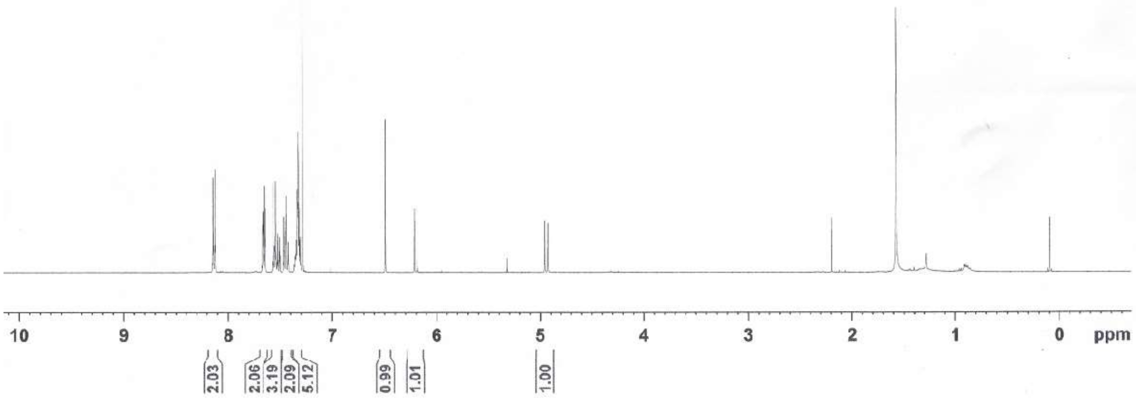

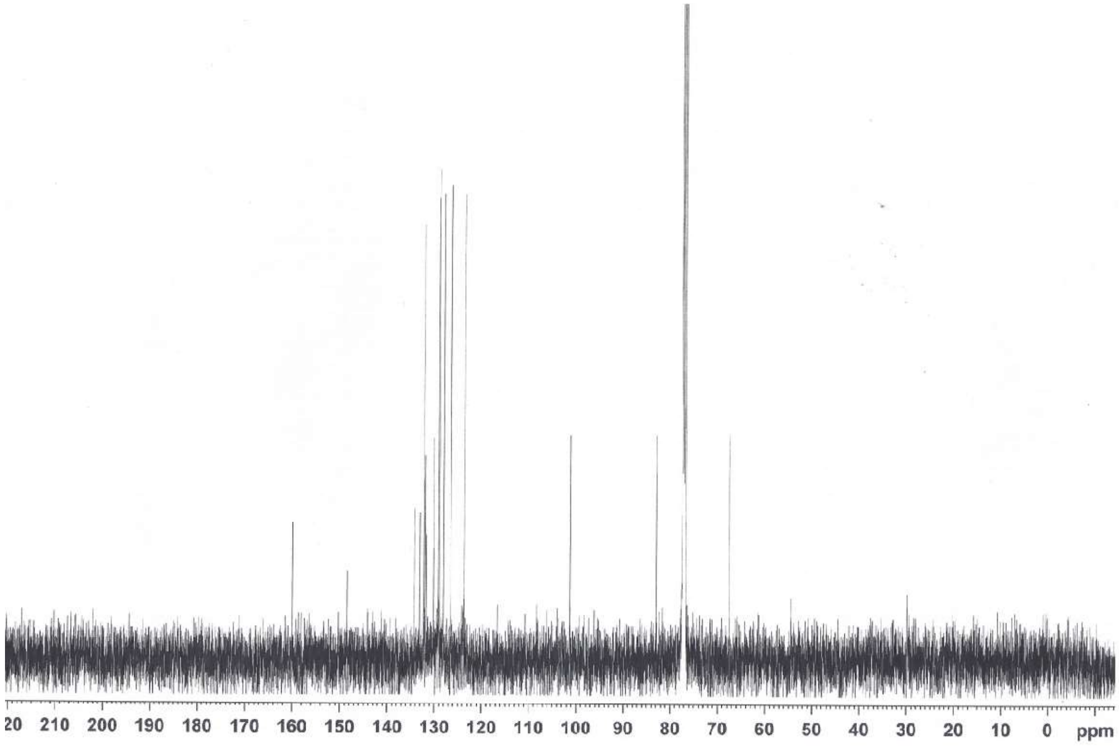

^{1}H and ^{13}C NMR spectra for (±)-**5e**:

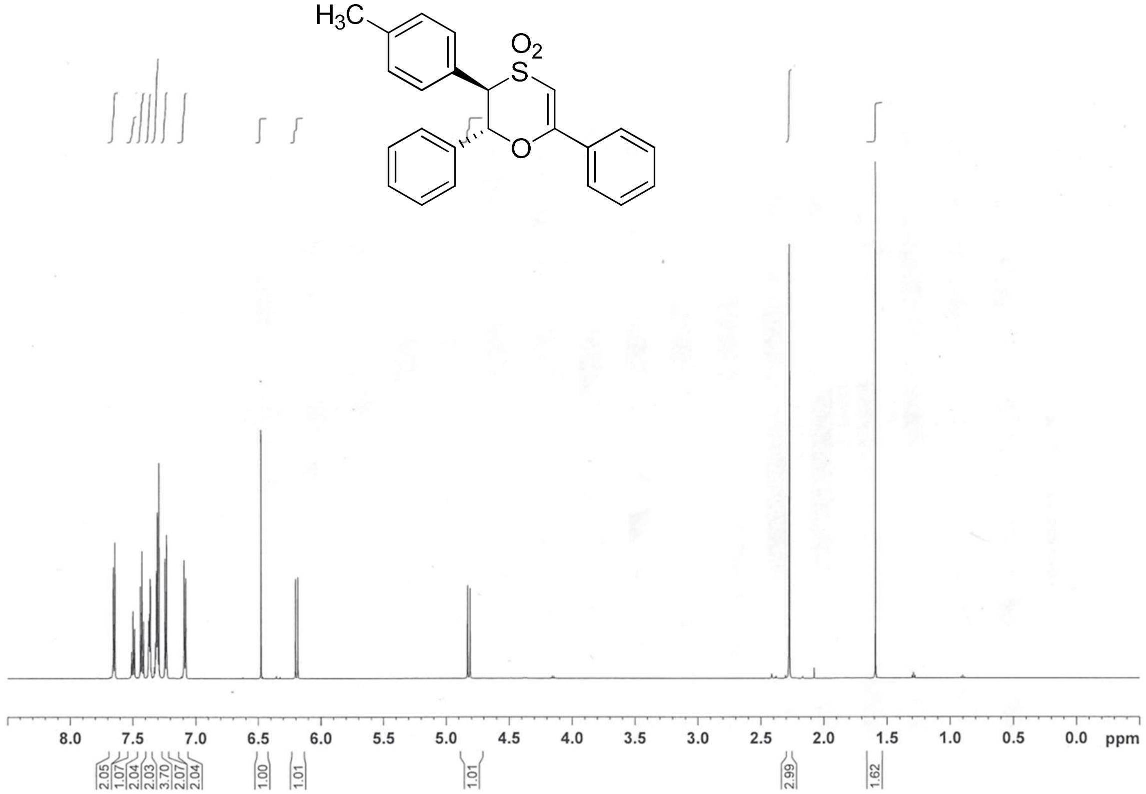

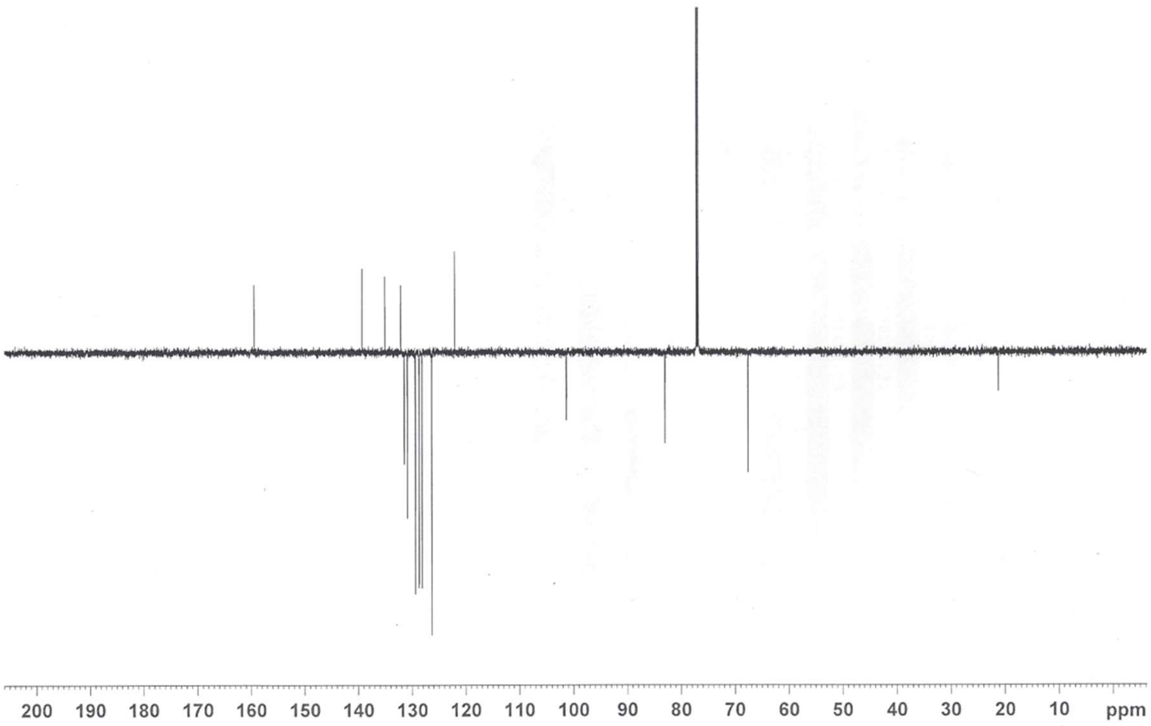

^{1}H and ^{13}C NMR spectra for (±)-**5f**:

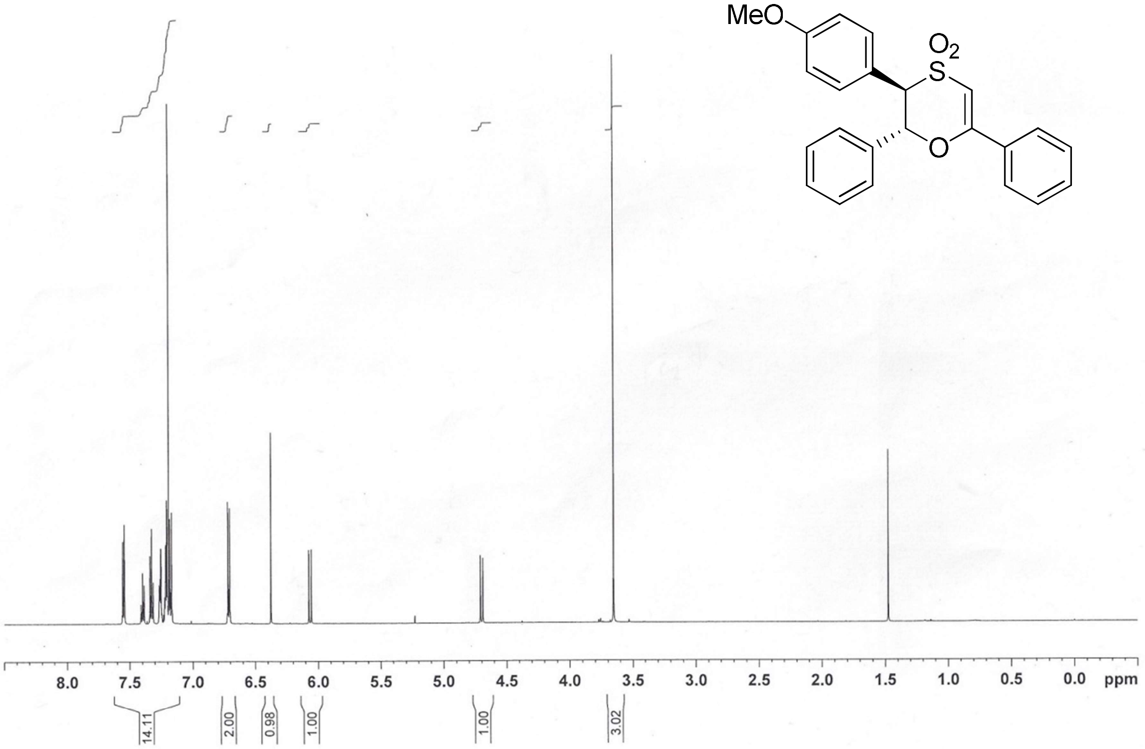

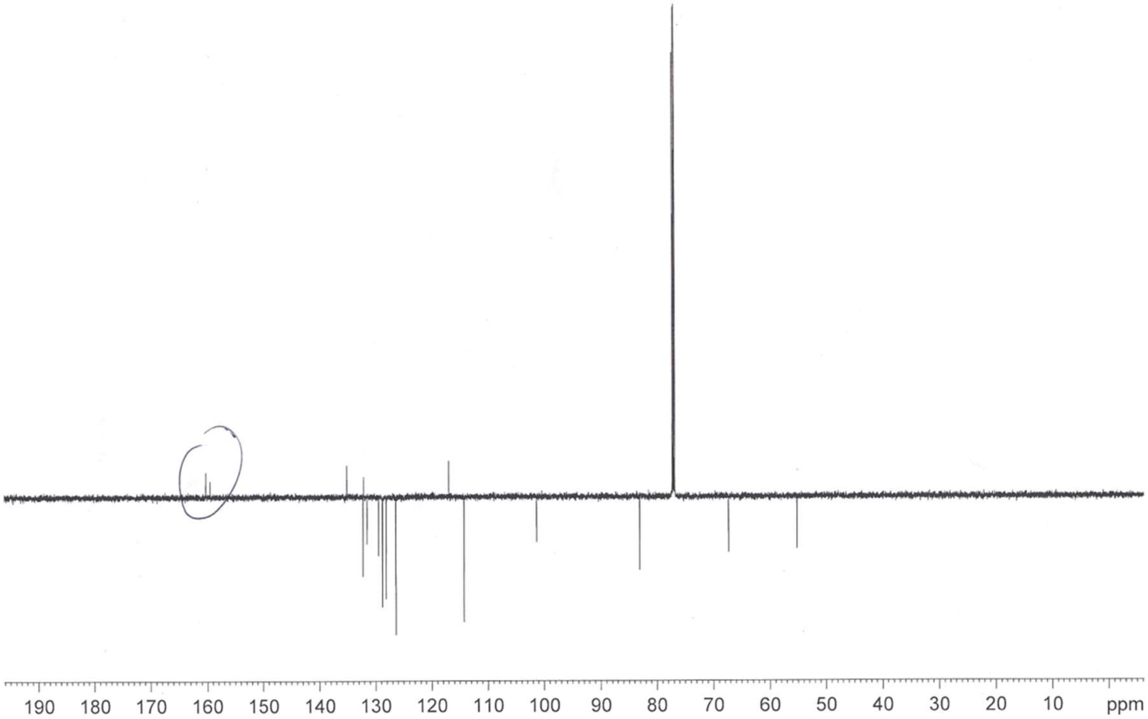

^{1}H and ^{13}C NMR spectra for (±)-**5g**:

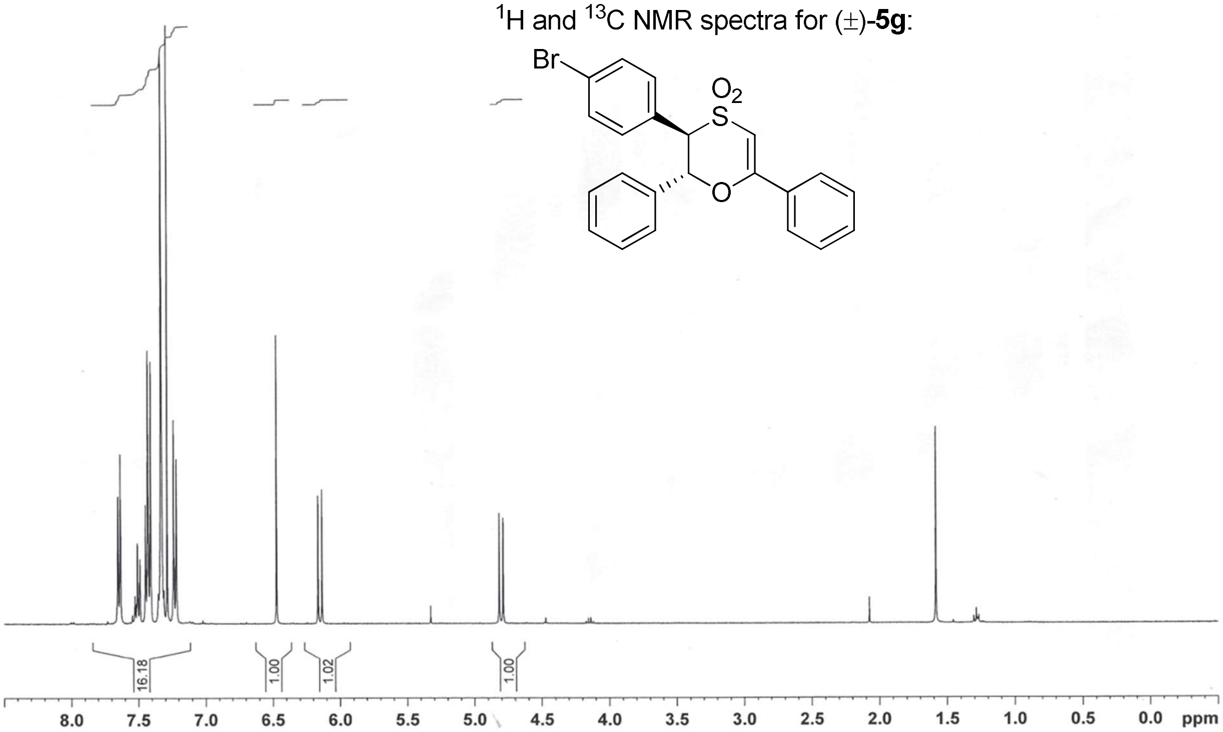

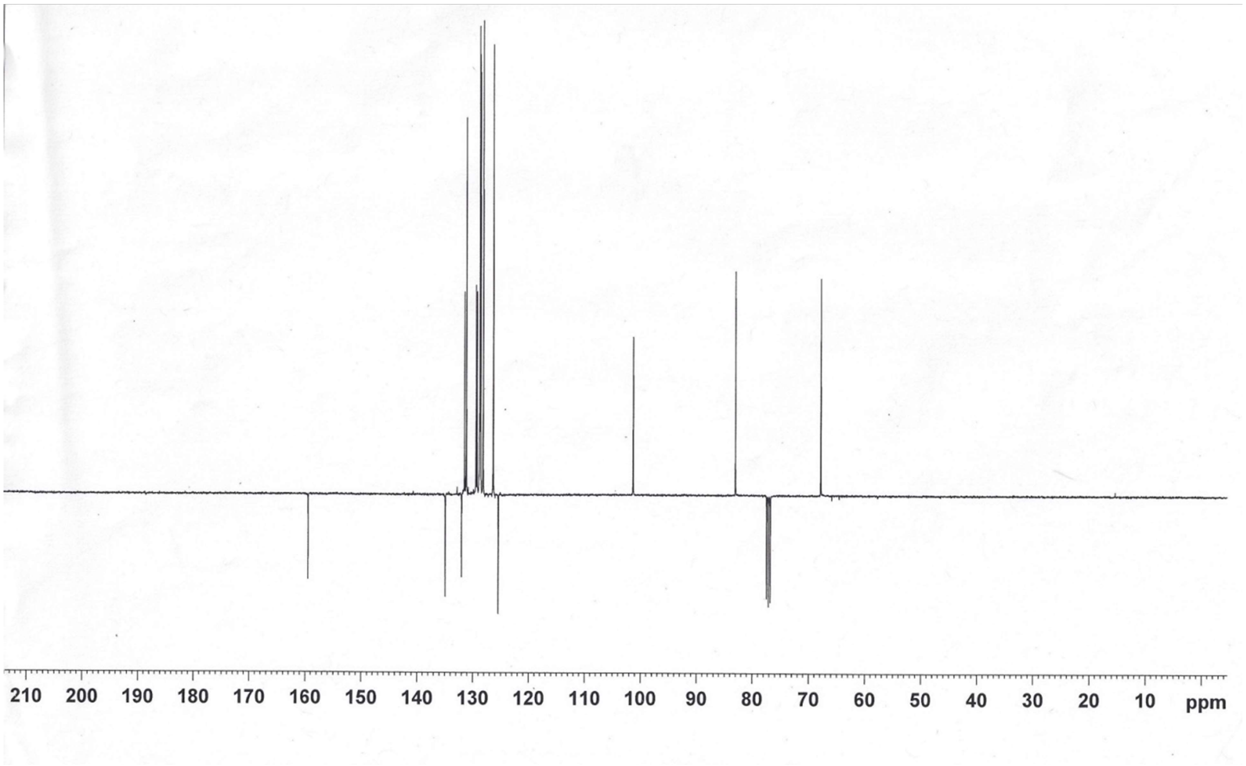

^{1}H and ^{13}C NMR spectra for (±)-**5h**:

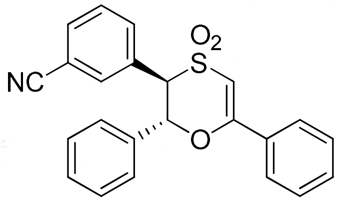

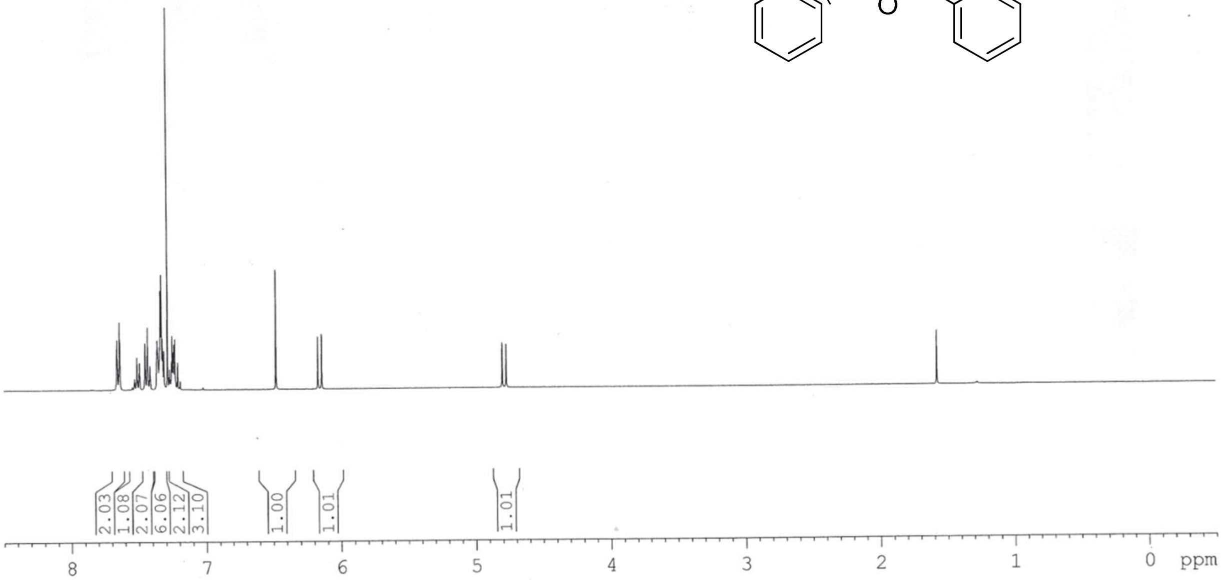

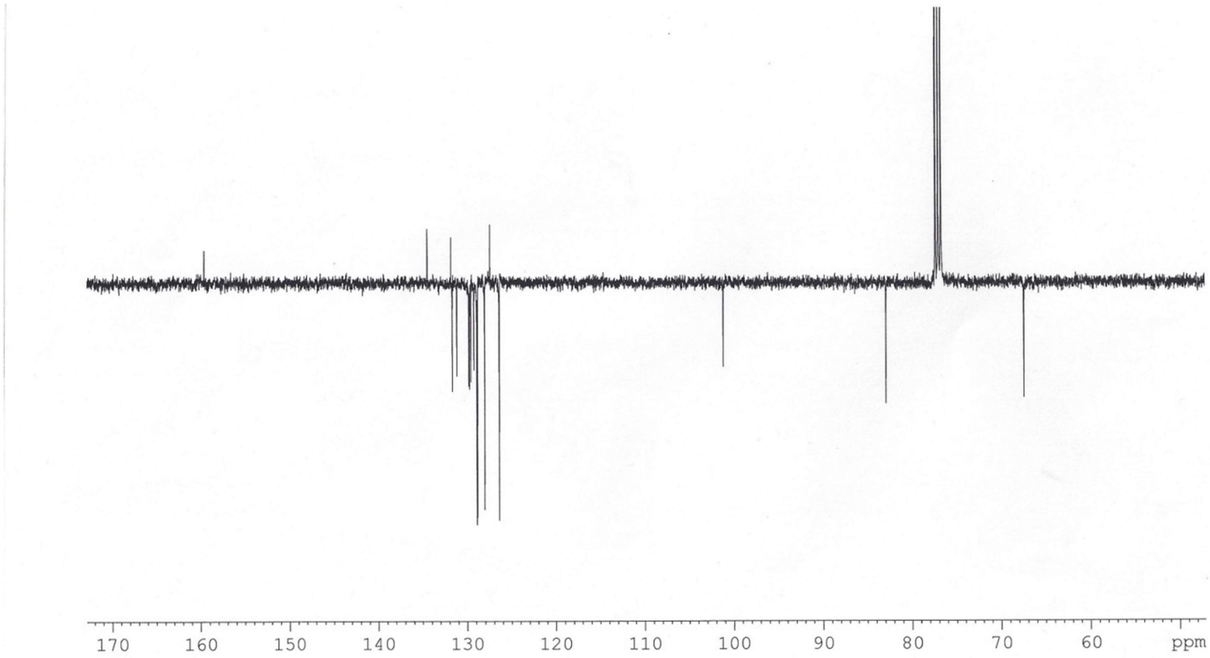

^{1}H and ^{13}C NMR spectra for (±)-**5i**:

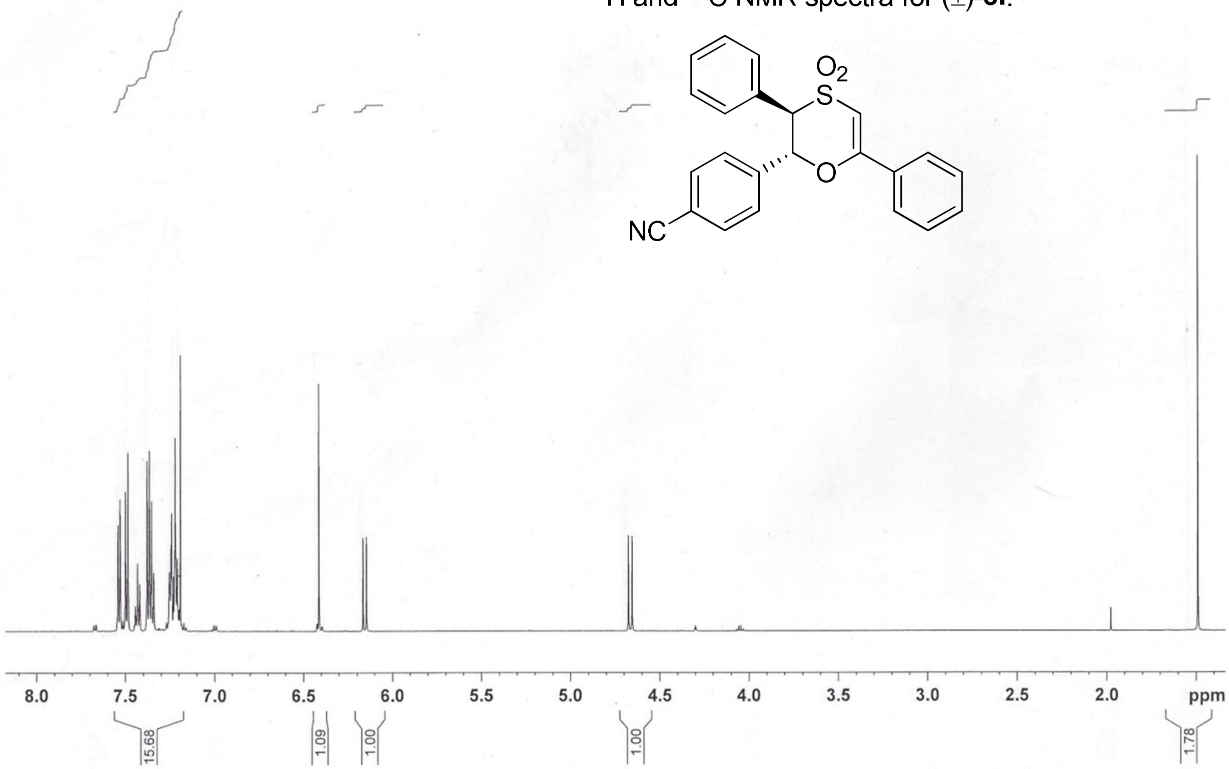

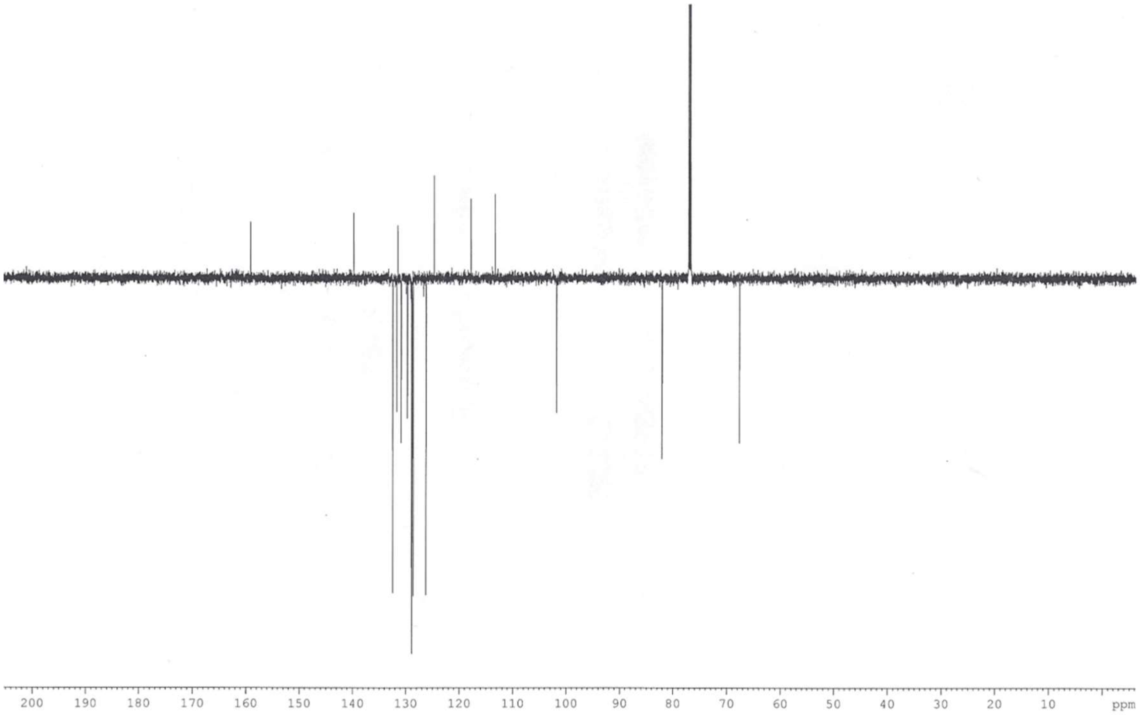

^{1}H and ^{13}C NMR spectra for (±)-**5j**:

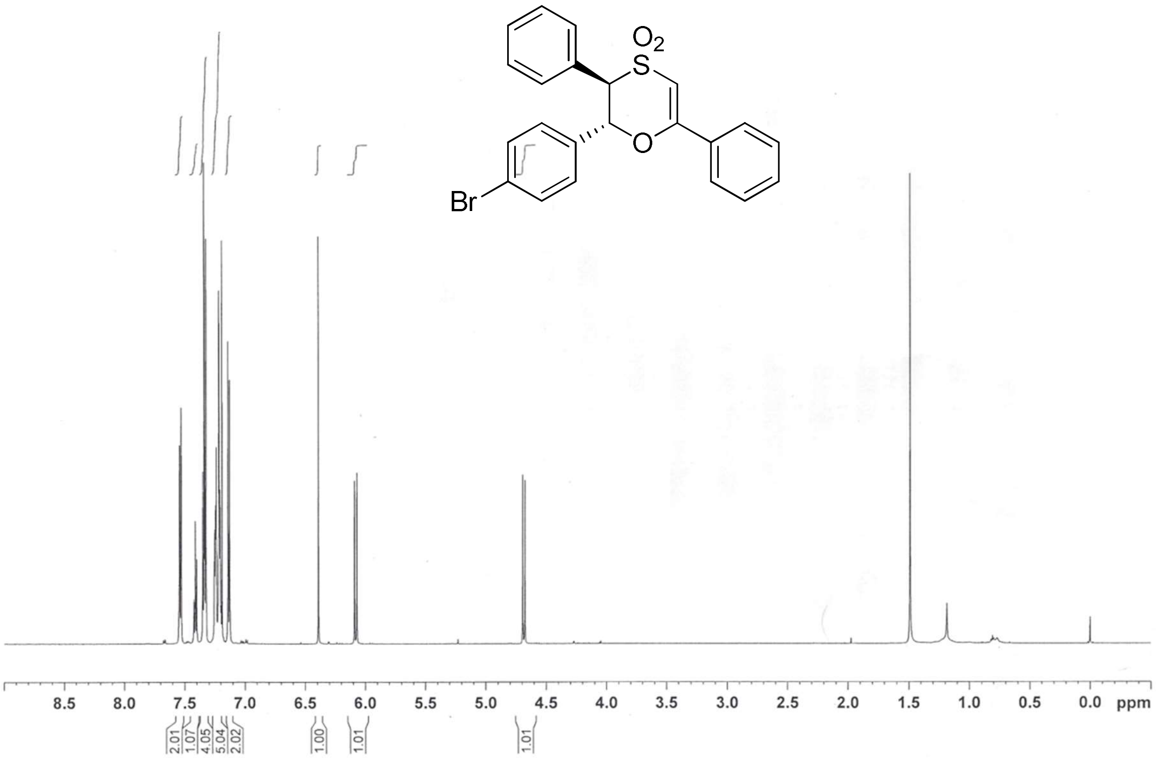

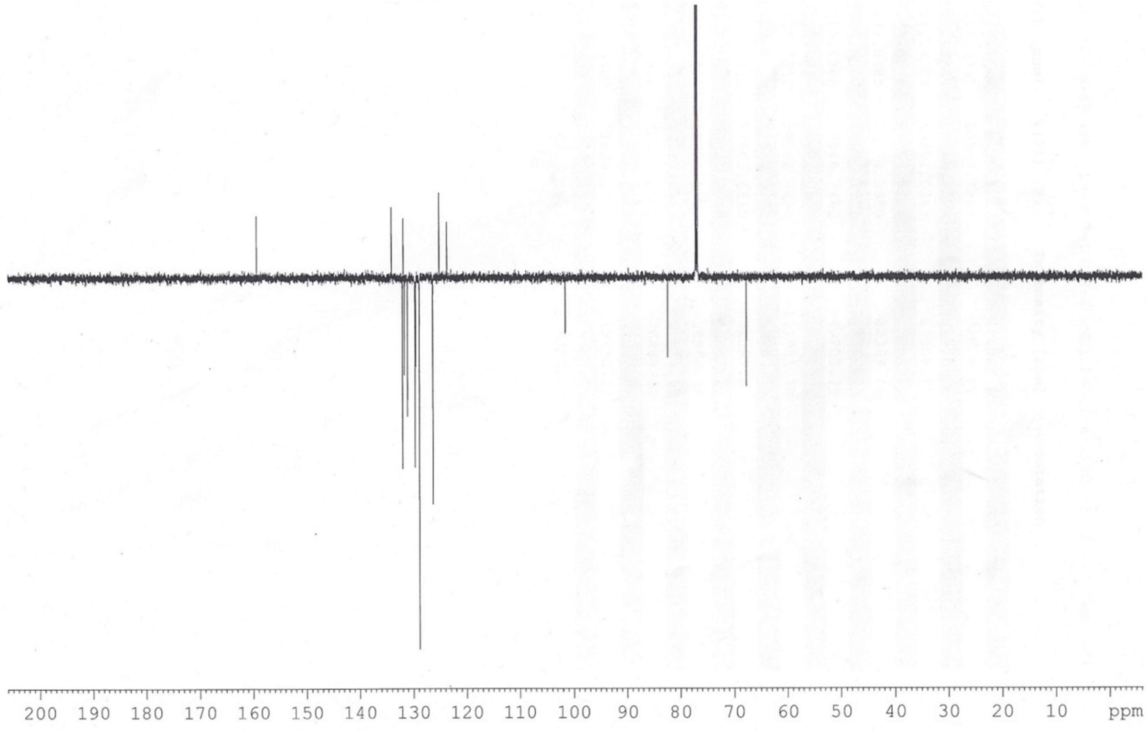

^{1}H and ^{13}C NMR spectra for (±)-**5k**:

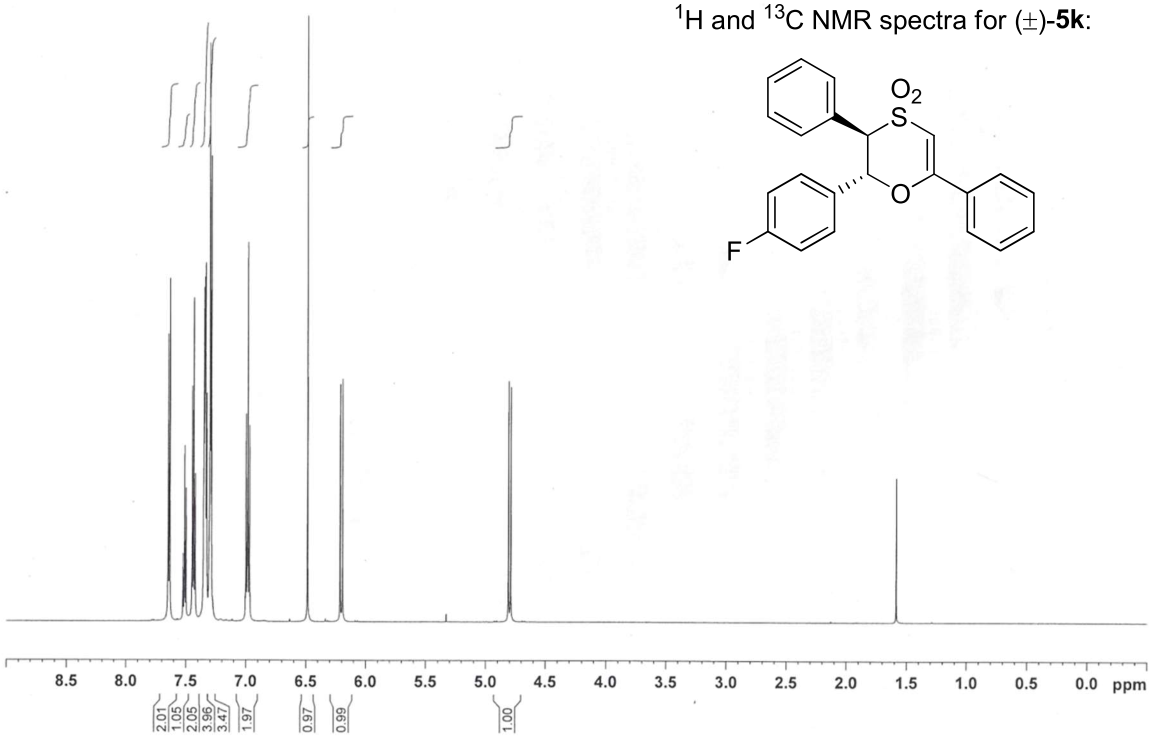

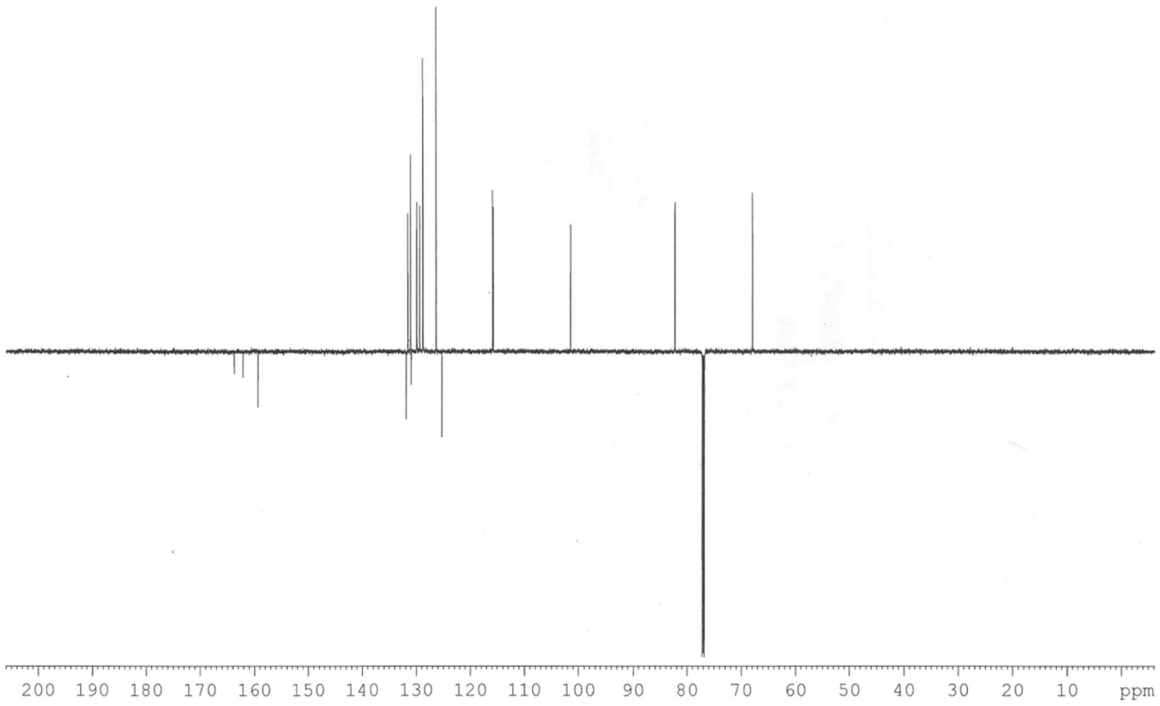

^{1}H and ^{13}C NMR spectra for (±)-**5l**:

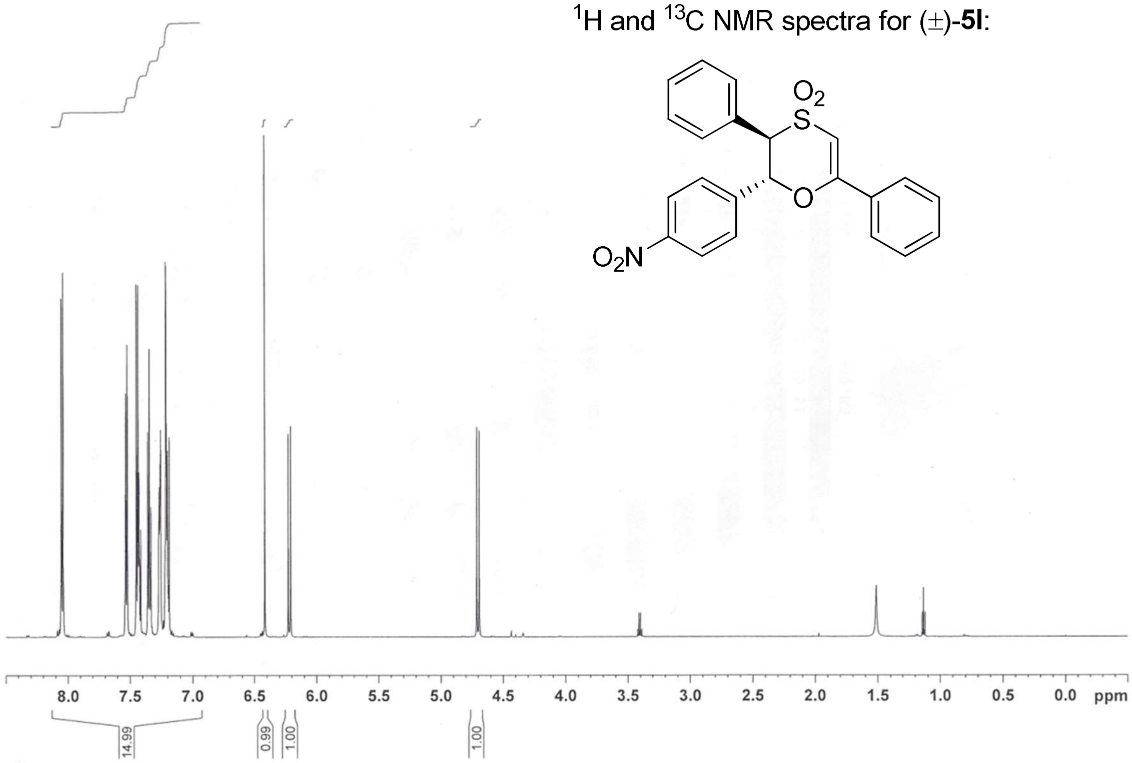

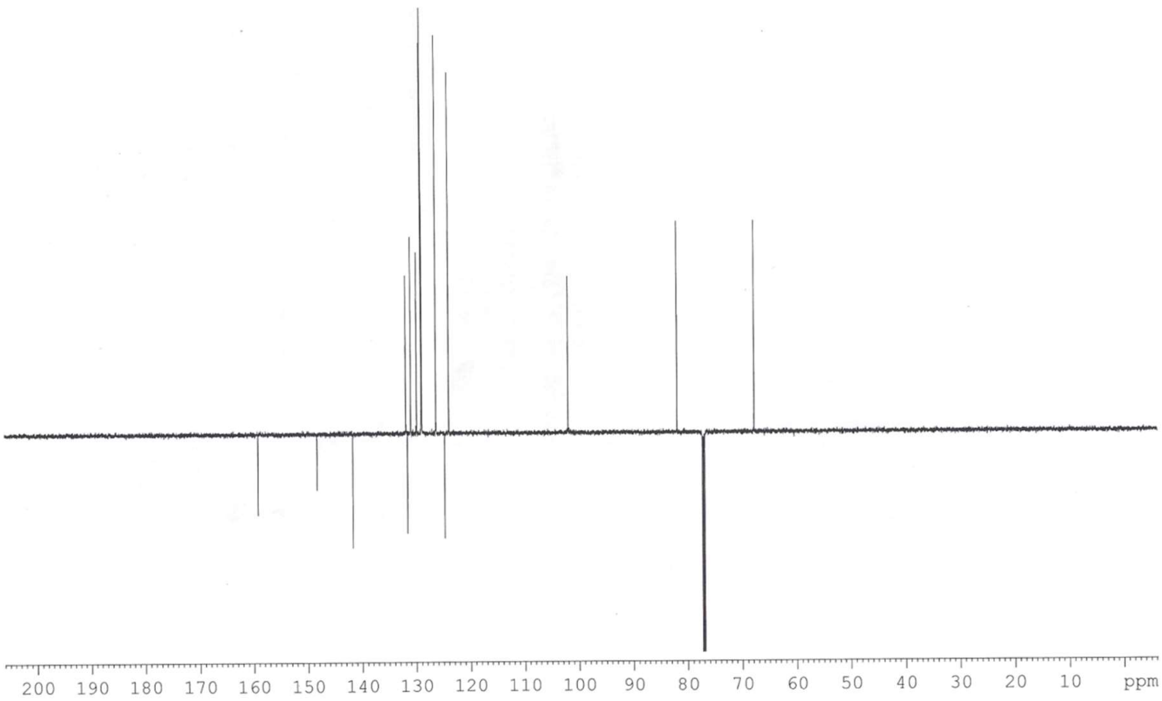

^1H and ^{13}C NMR spectra for (±)-**5m**:

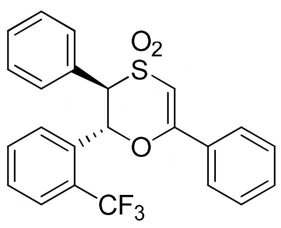

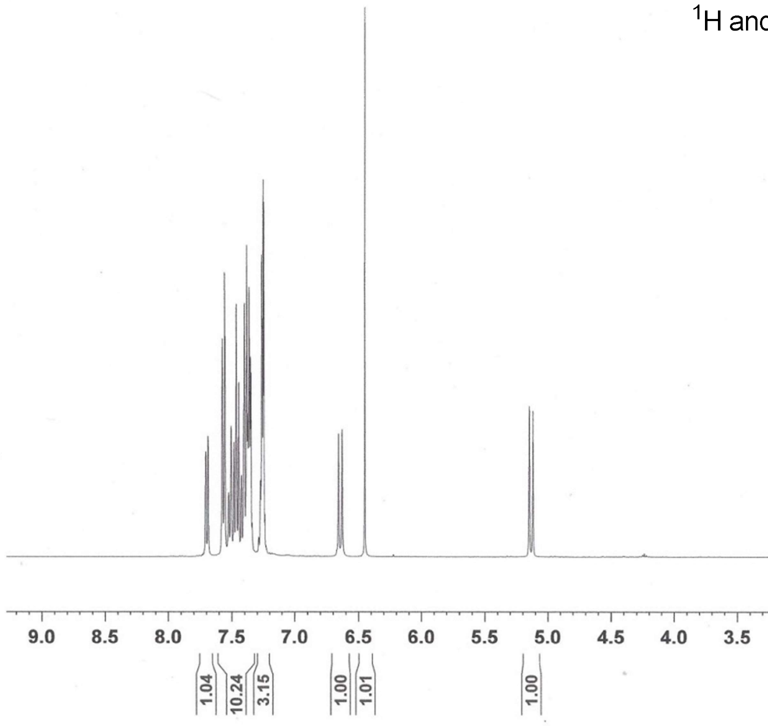

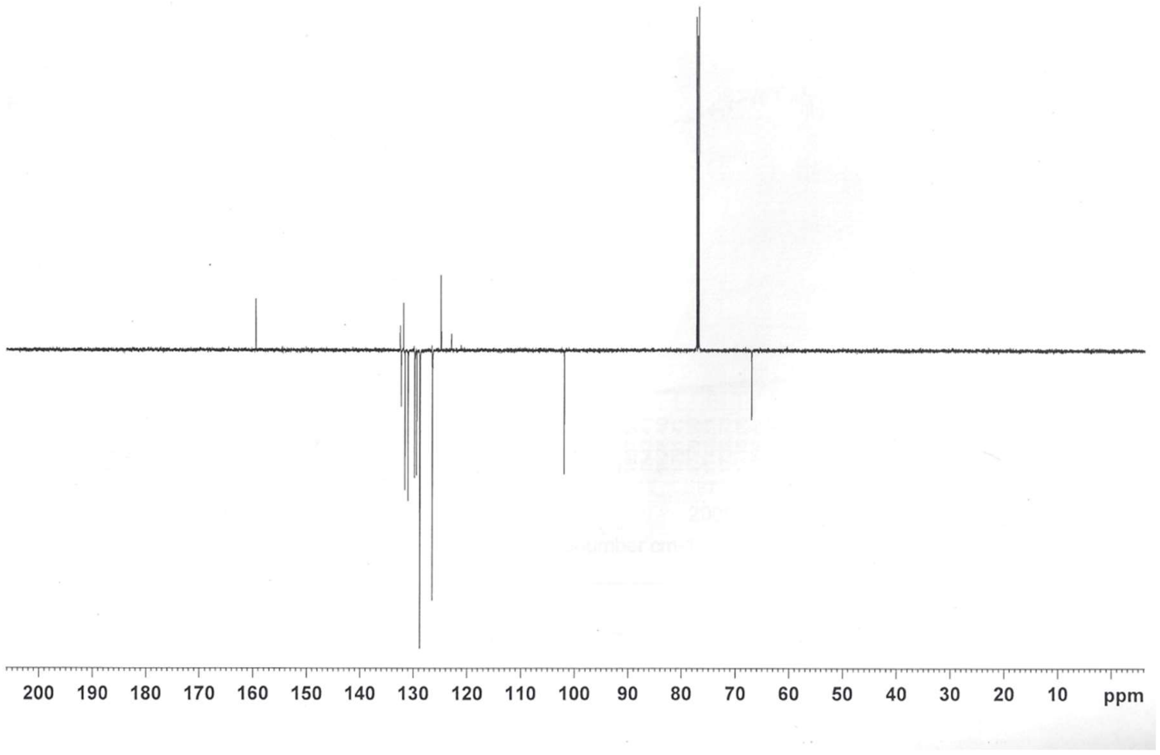

^{1}H and ^{13}C NMR spectra for (±)-**5n**:

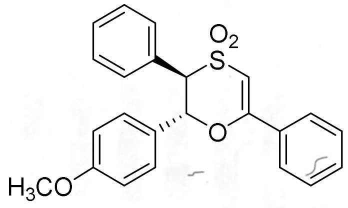

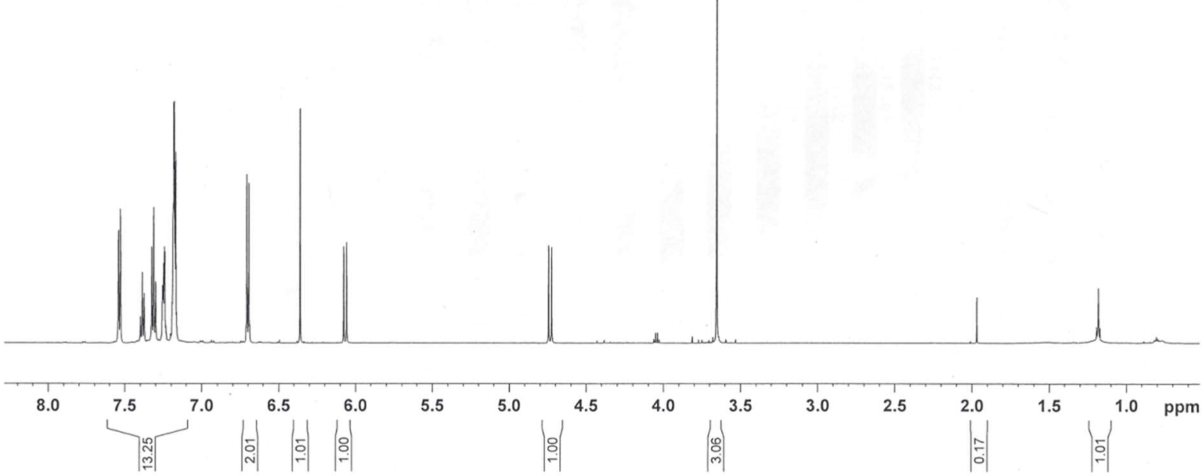

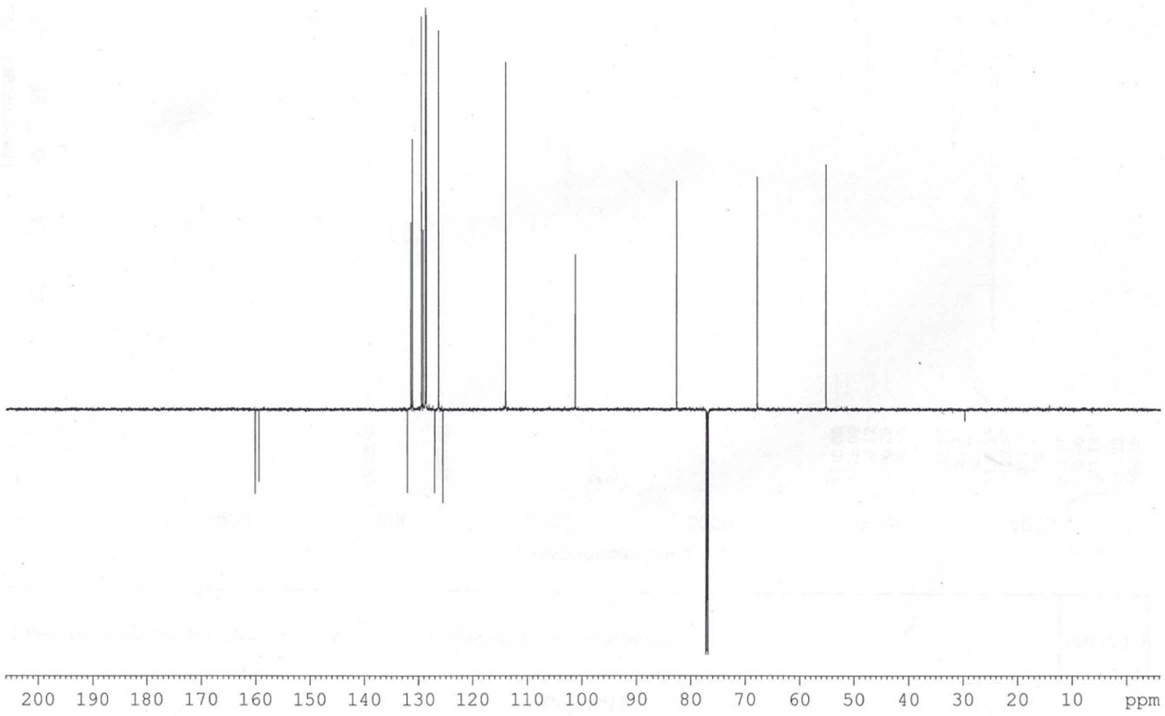

^{1}H and ^{13}C NMR spectra for (±)-**5o**:

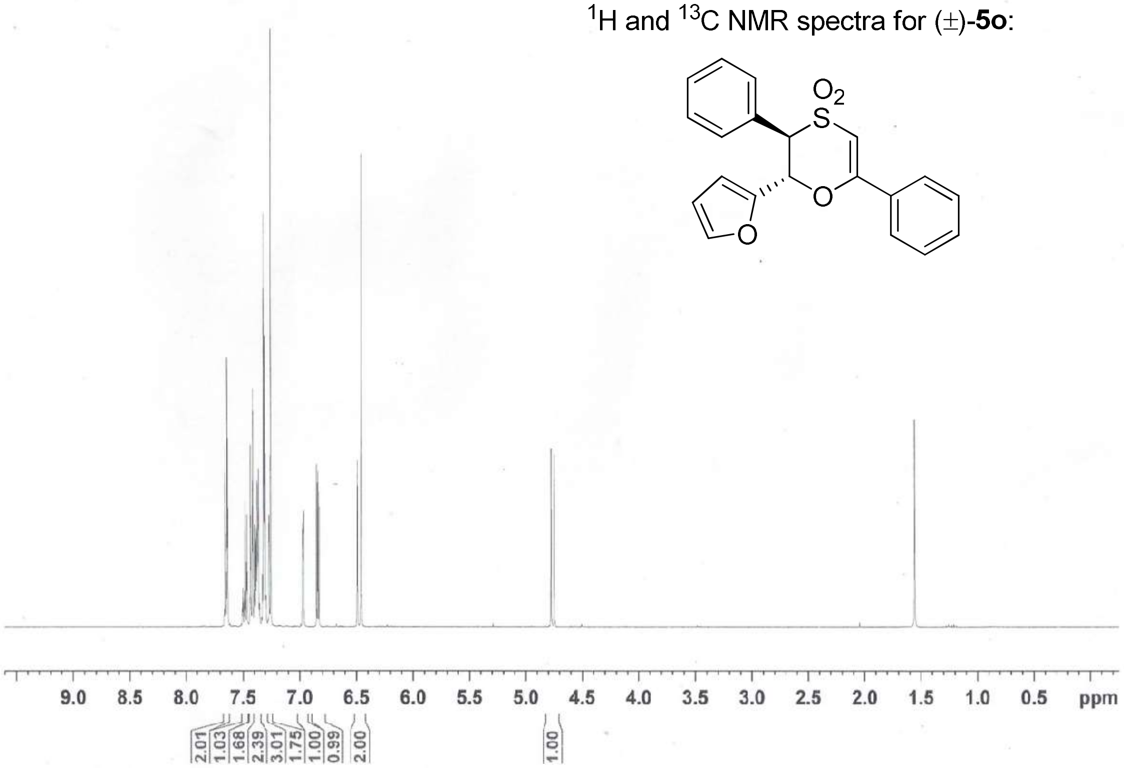

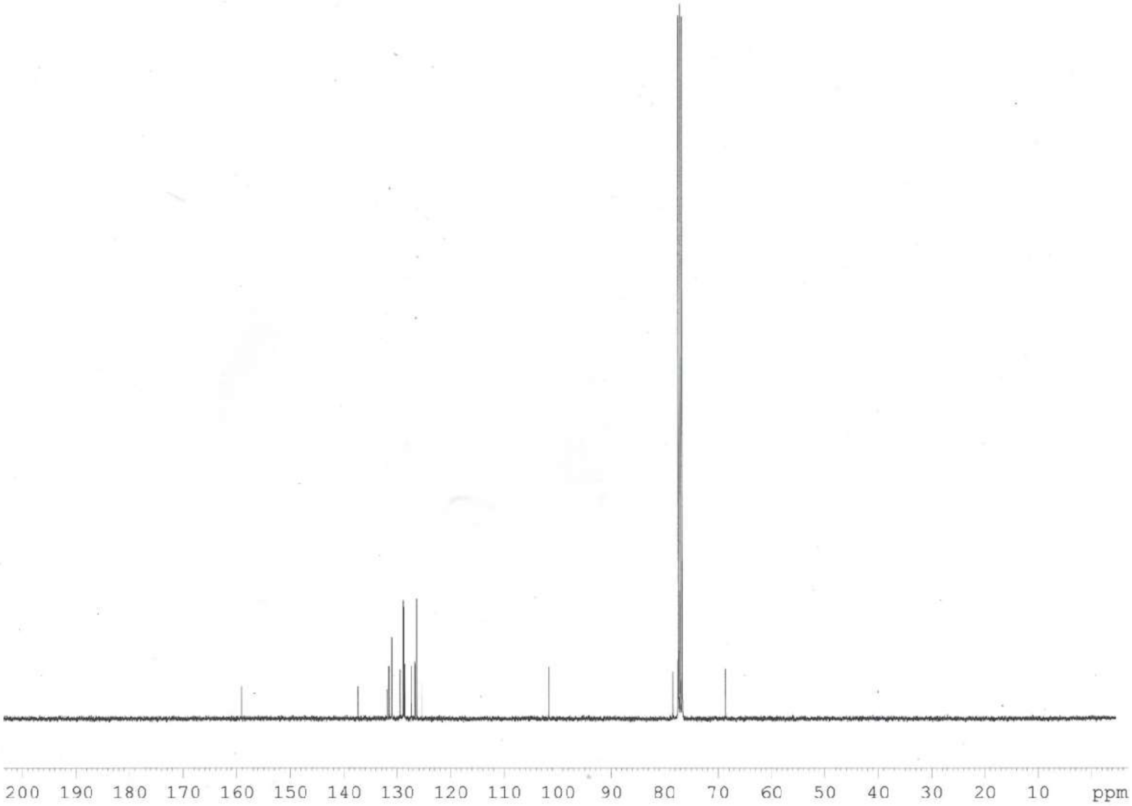

^{1}H and ^{13}C NMR spectra for (±)-**5p**:

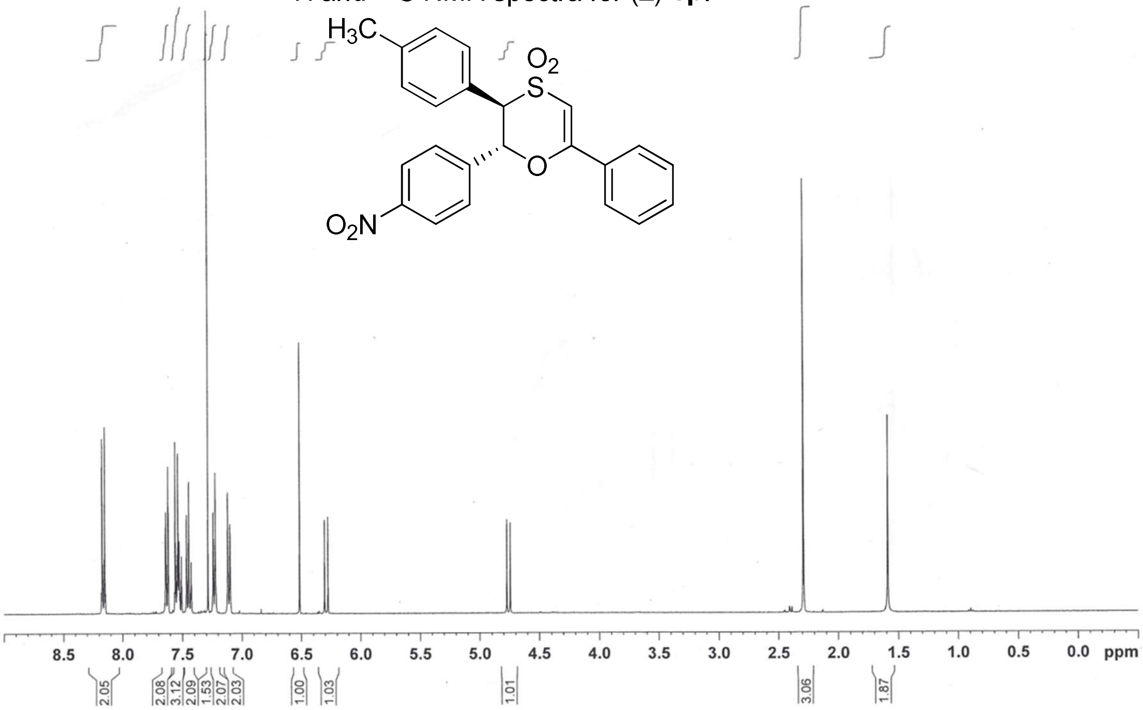

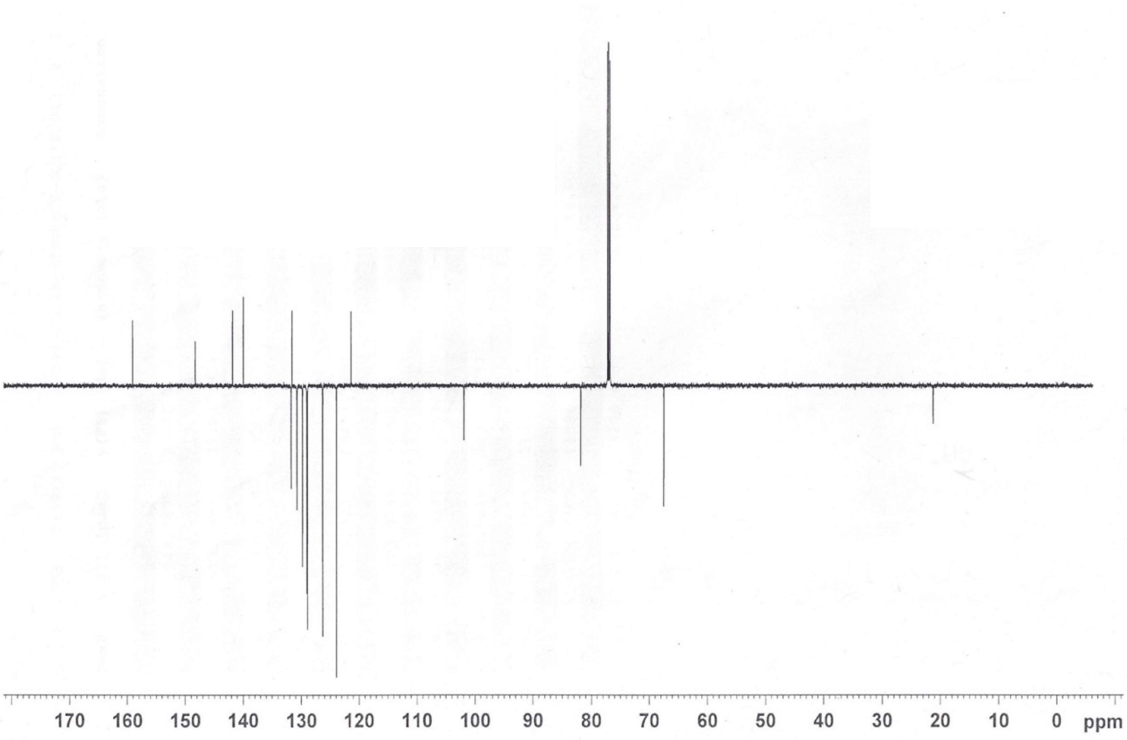

^1H and ^{13}C NMR spectra for (±)-**5q** (in DMSO-d_6):

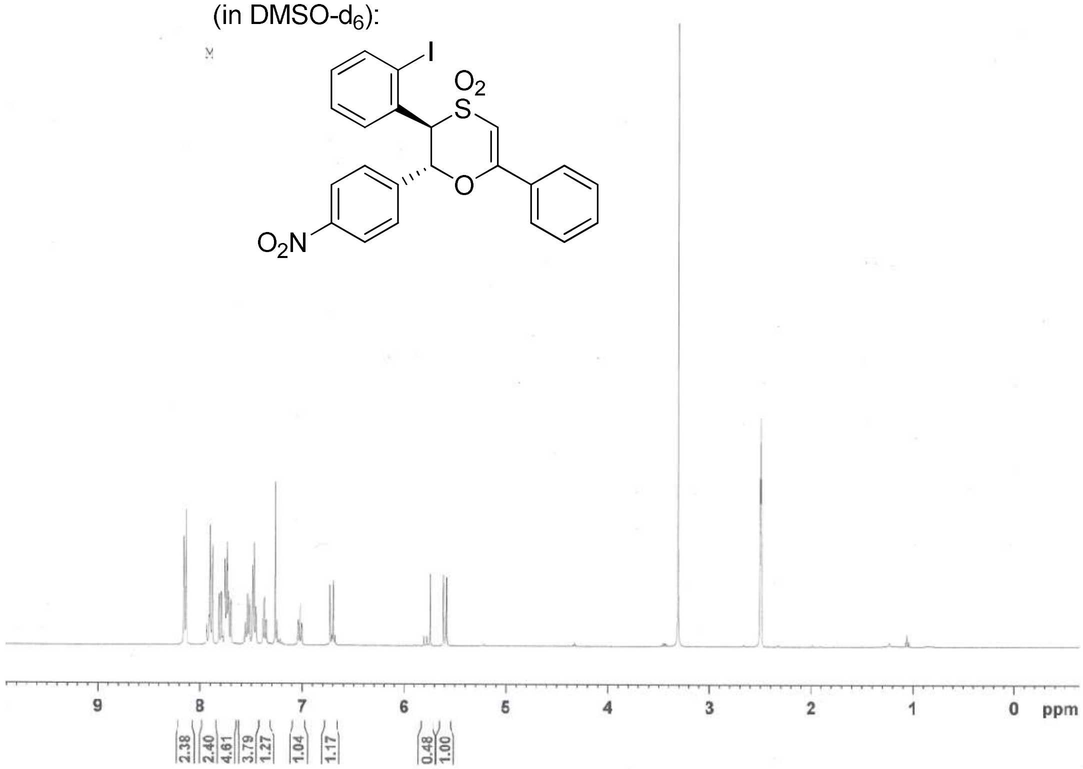

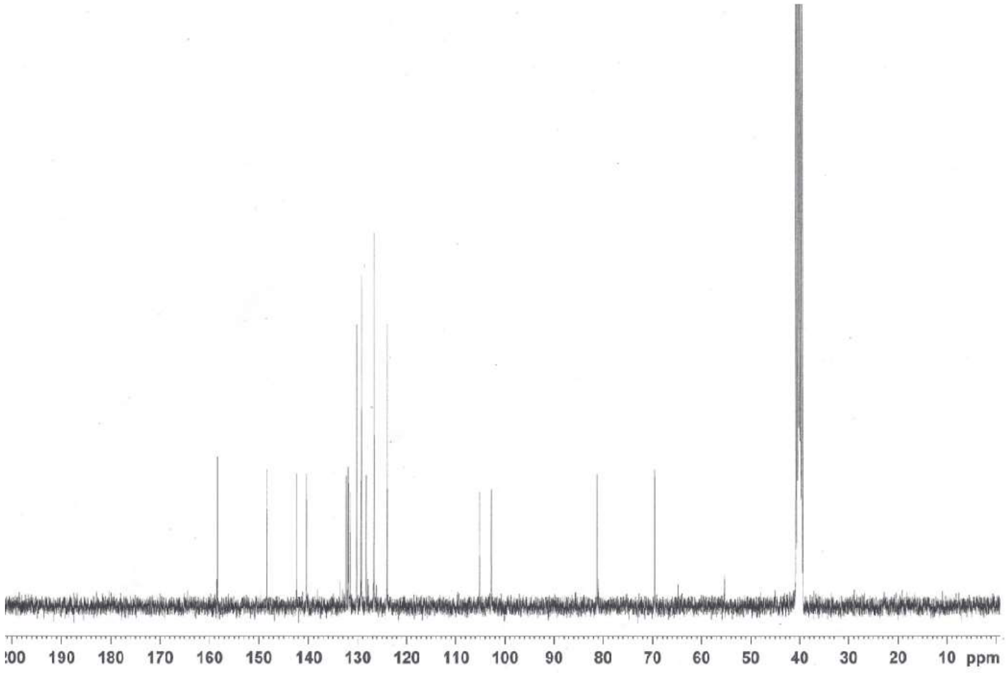

^{1}H and ^{13}C NMR spectra for (±)-**5r**:

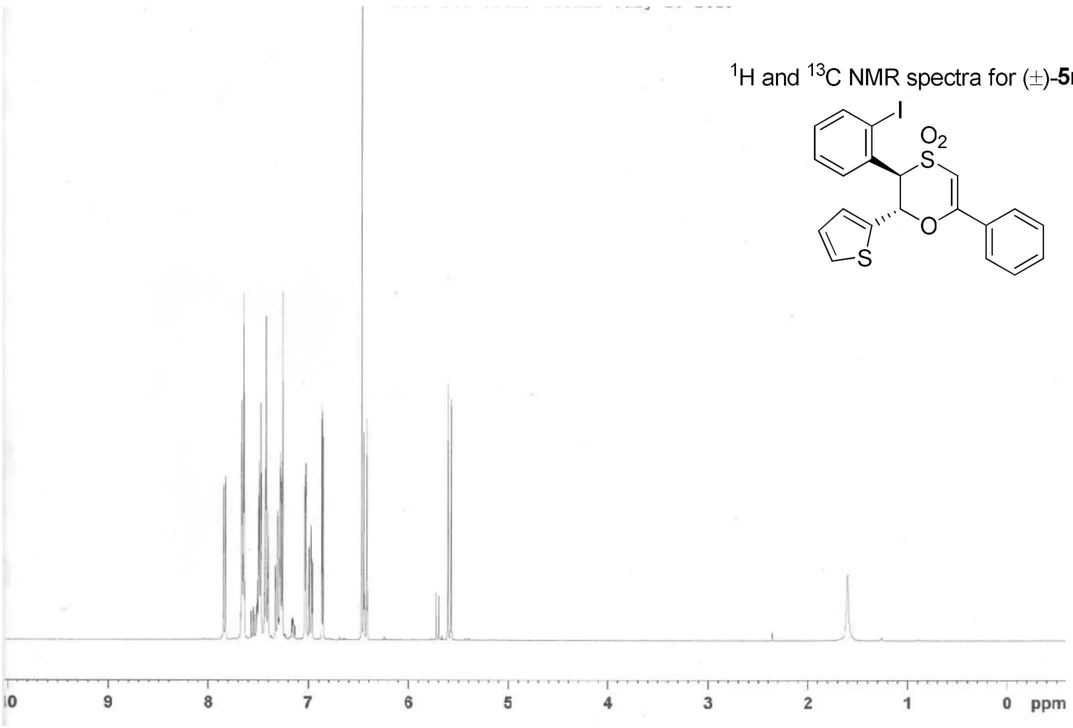

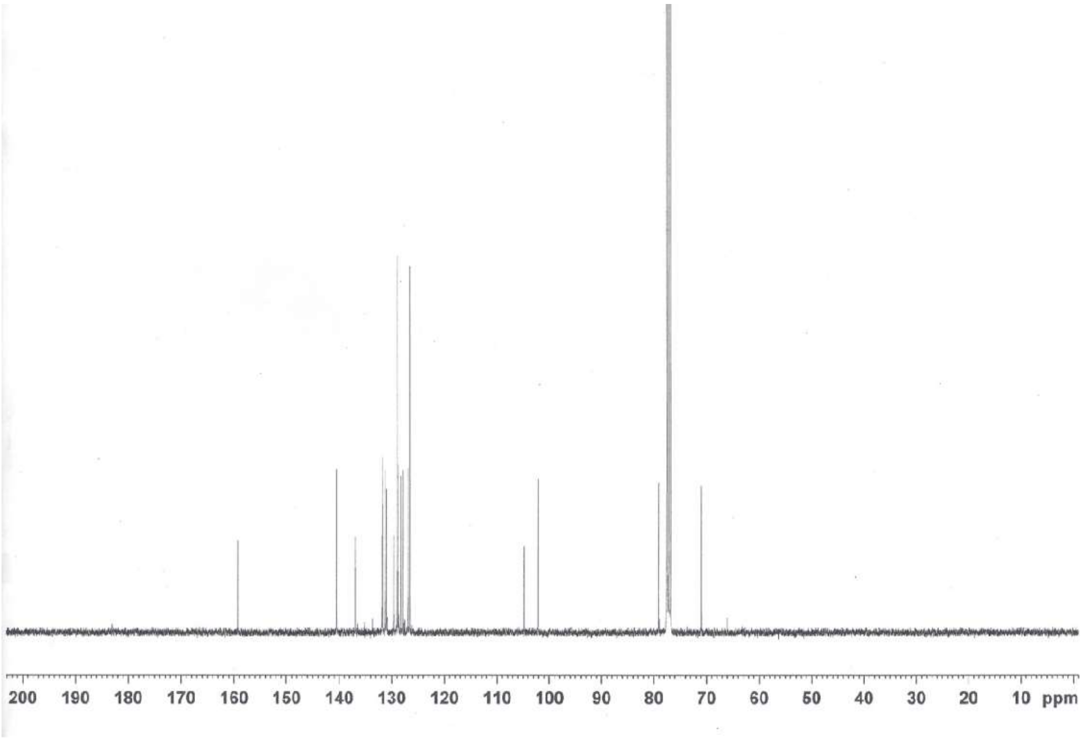

^{1}H and ^{13}C NMR spectra for (±)-**5s**:

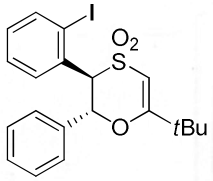

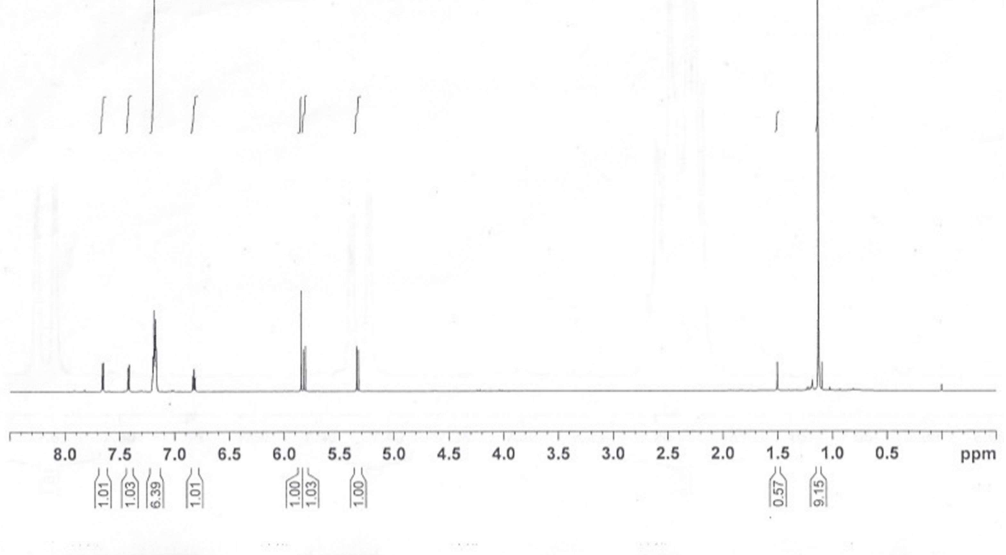

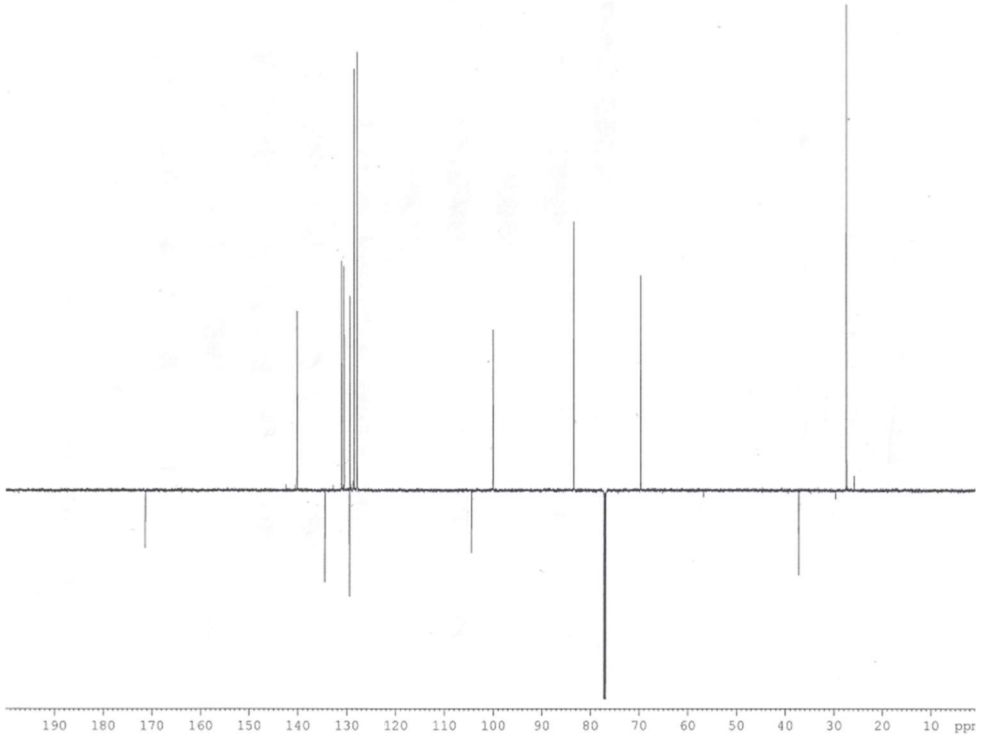

^1H and ^{13}C NMR spectra for (±)-**5t**:

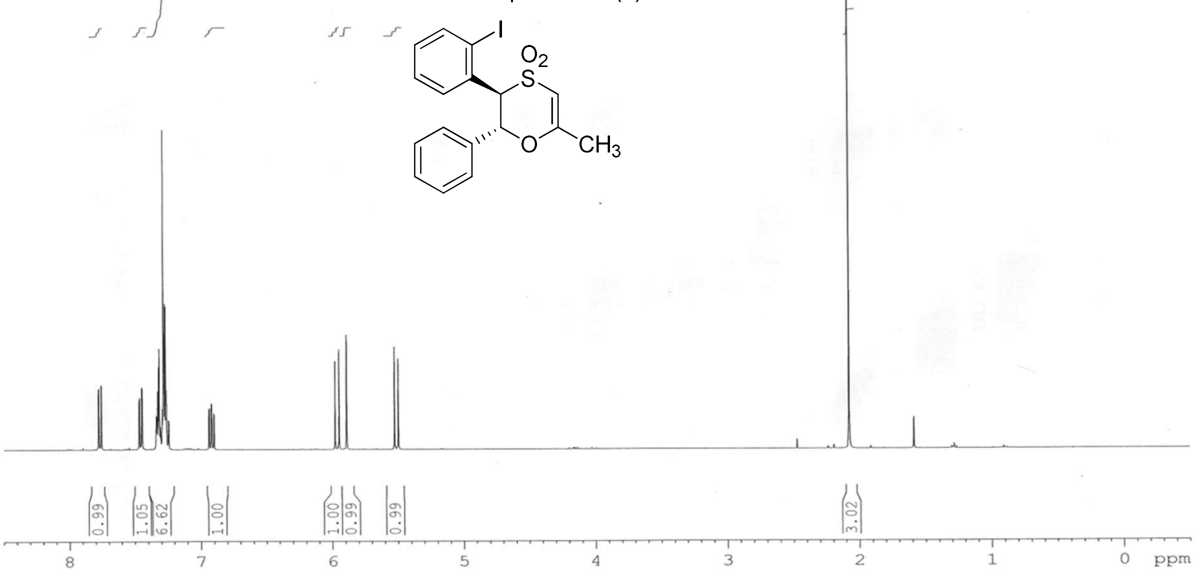

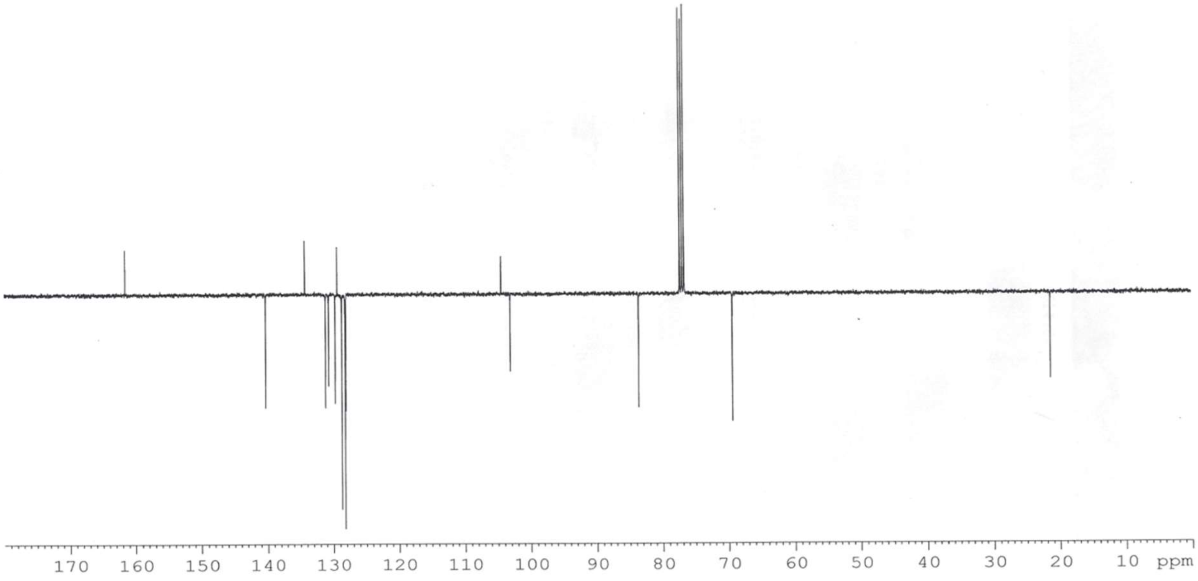

^{1}H and ^{13}C NMR spectra for **7Z/E** mixture (structure drawn is major, **7Z**):

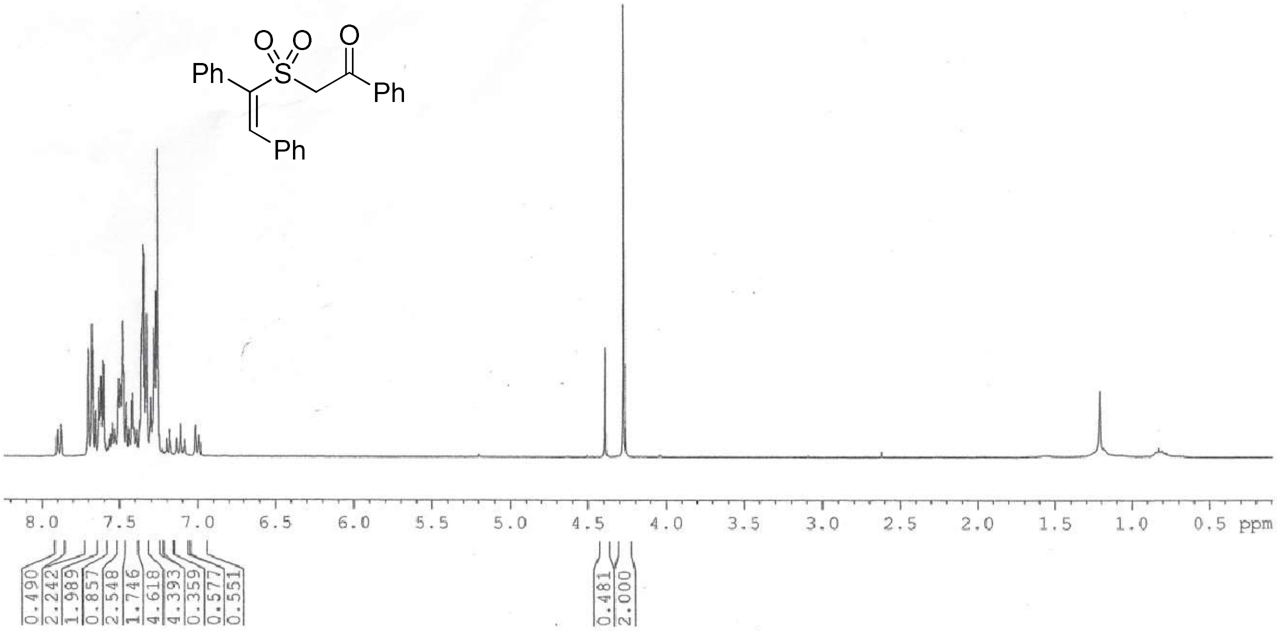

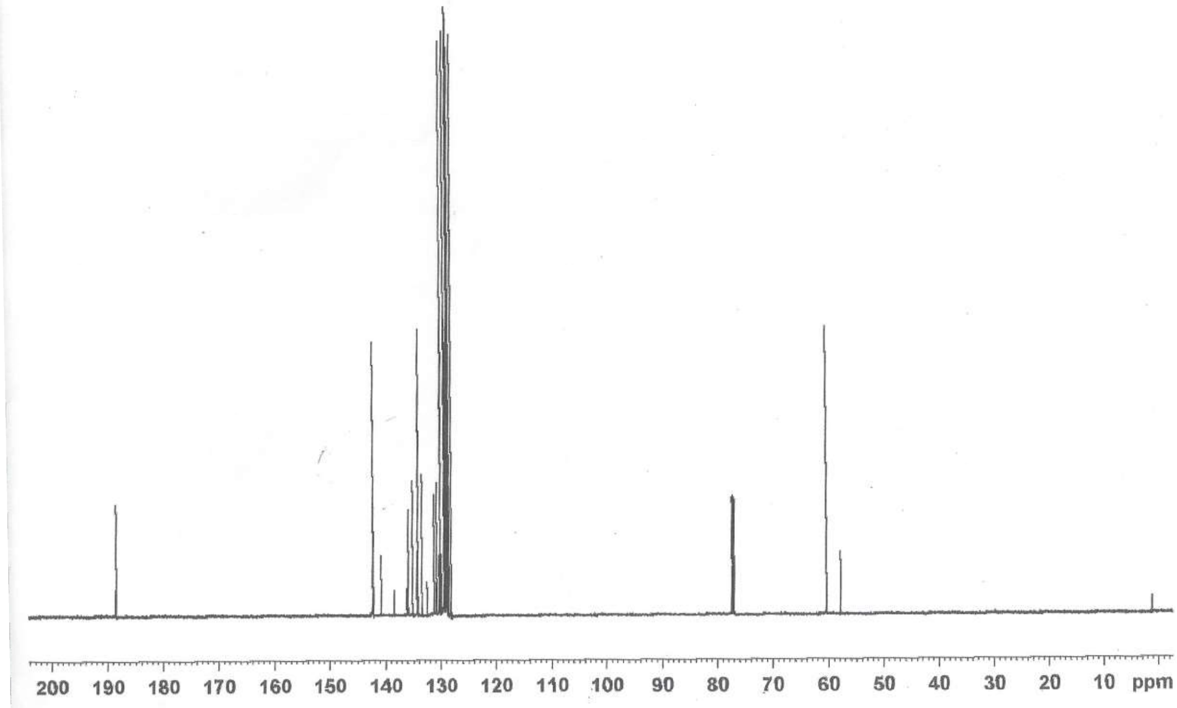

^{1}H and ^{13}C NMR spectra for **7E**:

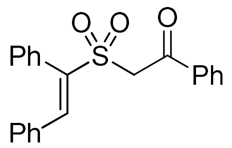

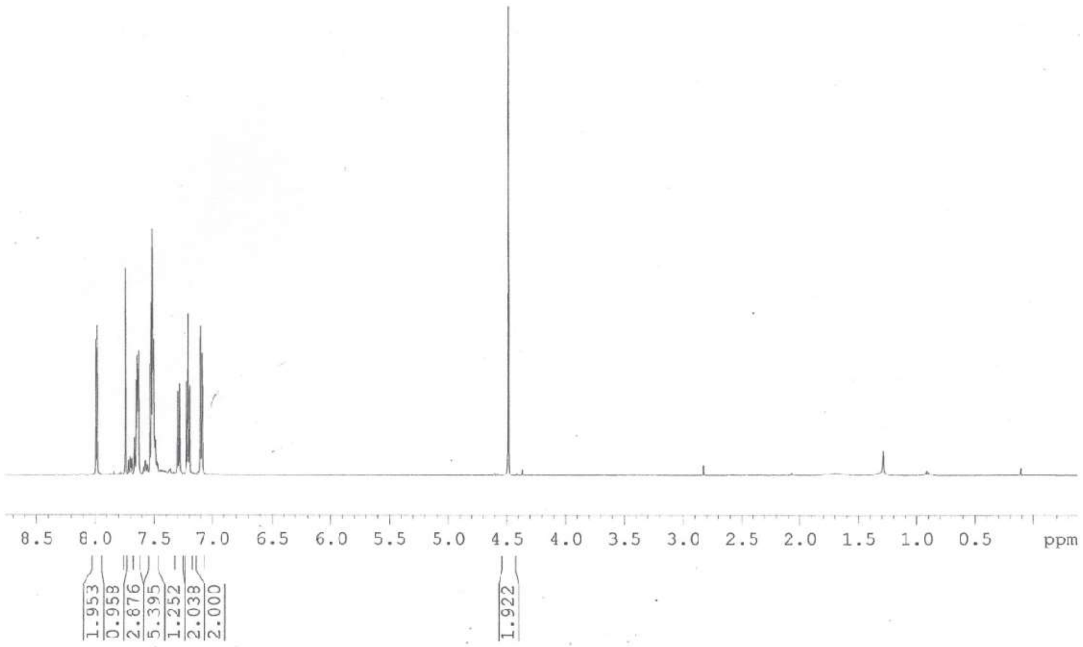

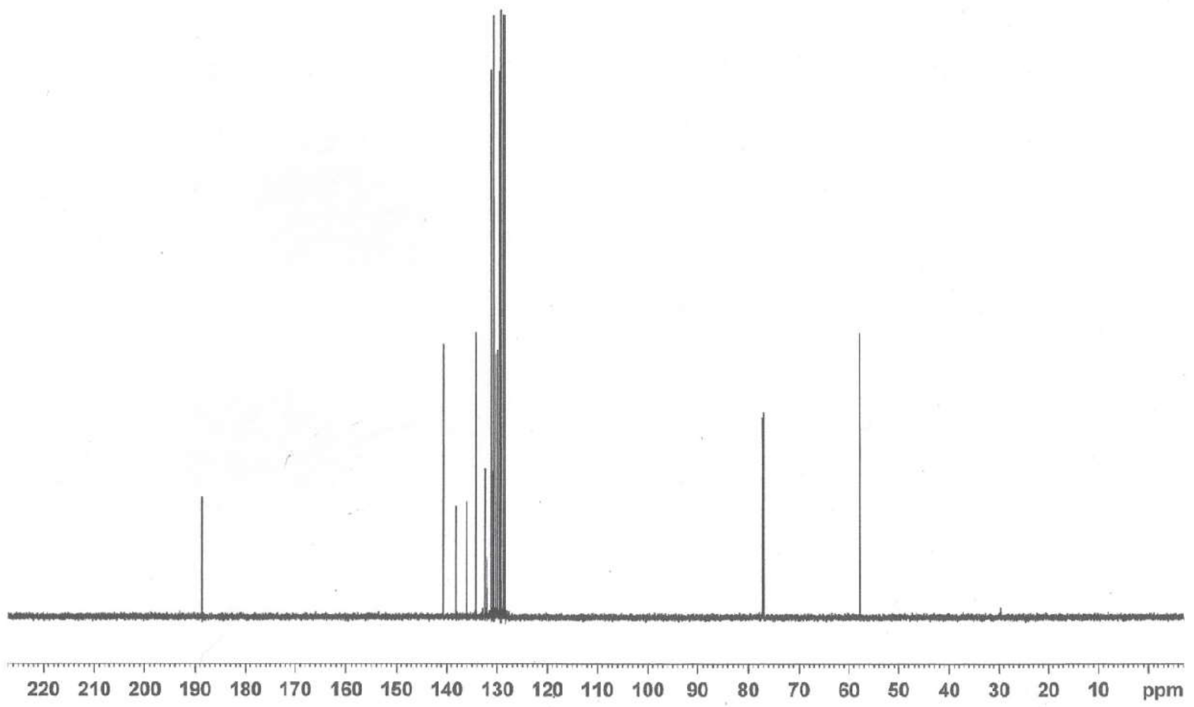

Cartesian coordinates and thermochemistry data of structures and transition states

Reactants:

Benzyl Alkynyl Sulfone 6

S	1.90761800	-1.78066300	0.15982200
O	2.47580200	-1.48330200	1.46736600
O	2.13415200	-3.09851700	-0.41656900
C	0.21560000	-1.48753900	0.23328700
C	-0.92379400	-1.09361800	0.19333500
C	2.03160300	0.85172300	-0.63246900
C	2.72312700	1.55416200	0.35557300
C	0.91026100	1.42784100	-1.23130600
C	2.29516600	2.81984500	0.74071600
H	3.59151400	1.10480900	0.82439100
C	0.48354200	2.69433900	-0.84618500
H	0.37122400	0.88241800	-1.99889900
C	1.17431700	3.39031800	0.14230000
H	2.83673900	3.36098000	1.50711500
H	-0.38652100	3.13605000	-1.31729900
H	0.84297000	4.37720700	0.44270100
C	-2.26610600	-0.60912700	0.16091100
C	-3.30566200	-1.42672400	-0.29852100
C	-2.52803900	0.70124500	0.58184500
C	-4.60188600	-0.93122400	-0.33113800
H	-3.08970500	-2.43720000	-0.62244700
C	-3.82812500	1.18601700	0.54131400
H	-1.71195100	1.32247900	0.93161400
C	-4.86333400	0.37176500	0.08707800
H	-5.40888400	-1.56134800	-0.68379300
H	-4.03441900	2.19845100	0.86564200
H	-5.87672200	0.75366500	0.05896100
C	2.46072000	-0.52958300	-1.02419200
H	3.54580800	-0.66074400	-1.02270200
H	2.05408600	-0.84825100	-1.98409700

```
Zero-point correction=                           0.231366
(Hartree/Particle)
 Thermal correction to Energy=                    0.247082
 Thermal correction to Enthalpy=                  0.248027
 Thermal correction to Gibbs Free Energy=         0.184354
 Sum of electronic and zero-point Energies=          -1126.987555
 Sum of electronic and thermal Energies=             -1126.971838
 Sum of electronic and thermal Enthalpies=           -1126.970894
```

Sum of electronic and thermal Free Energies= -1127.034567

Anionic Sulfone Lithiated on One Oxygen (8a)

S	1.55098300	1.93804100	-0.05132900
O	1.92611400	1.93386000	-1.49848100
O	1.68986600	3.22081200	0.64082500
C	-0.15528400	1.57571900	-0.10611900
C	-1.23730000	1.03991900	-0.04411800
C	2.39839100	0.72863700	0.72700400
C	2.19247500	-0.67955700	0.40311900
C	1.58880900	-1.15610500	-0.77869300
C	2.64240200	-1.64660500	1.32657300
C	1.44980700	-2.52110100	-1.01444200
H	1.22026900	-0.45921400	-1.52270700
C	2.50601900	-3.00425700	1.08116900
H	3.10467200	-1.31220400	2.24946900
C	1.90698300	-3.45845600	-0.09438900
H	0.97578300	-2.85016300	-1.93280500
H	2.86556400	-3.71555800	1.81648600
H	1.79793000	-4.51900500	-0.28483900
Li	3.69689100	1.10978600	-1.49630700
H	2.55533900	0.98423900	1.76836700
C	-2.52707000	0.42384100	-0.00764700
C	-3.54124300	0.93956200	0.80887900
C	-2.77090500	-0.71259200	-0.79045500
C	-4.78548900	0.32322800	0.83599400
H	-3.34585200	1.81664600	1.41343700
C	-4.01799300	-1.32173100	-0.75495900
H	-1.97884300	-1.10816900	-1.41510500
C	-5.02611800	-0.80576600	0.05638600
H	-5.56867100	0.72401700	1.46782800
H	-4.20343000	-2.20055000	-1.36042100
H	-5.99790600	-1.28386600	0.08191200

Zero-point correction= 0.220260 (Hartree/Particle)
Thermal correction to Energy= 0.236936
Thermal correction to Enthalpy= 0.237880
Thermal correction to Gibbs Free Energy= 0.173573
Sum of electronic and zero-point Energies= -1133.986103
Sum of electronic and thermal Energies= -1133.969427
Sum of electronic and thermal Enthalpies= -1133.968483
Sum of electronic and thermal Free Energies= -1134.032790

Anionic Sulfone Lithiated on One Oxygen (8b)

S	1.11170100	-1.85819500	-0.24224800
O	1.03527600	-3.22141400	0.35975900
O	1.53138000	-1.78706200	-1.64411900
C	-0.54791900	-1.31324700	-0.25554900
C	-1.60004800	-0.72413800	-0.16984000
C	2.07380700	-0.95728000	0.79102800
C	2.50524500	0.40392600	0.44566300
C	3.02783000	0.75670700	-0.81127500
C	2.45260300	1.40493800	1.43132100
C	3.45568300	2.05444800	-1.06721200
H	3.09898900	0.00338700	-1.58514500
C	2.90169600	2.69560000	1.17772800
H	2.05273500	1.15999500	2.40999600
C	3.40091500	3.03367900	-0.07757800
H	3.84919300	2.29880600	-2.04770600
H	2.85122600	3.44251700	1.96211000
H	3.74574900	4.04054100	-0.27985600
C	-2.86388500	-0.05895600	-0.09281900
C	-3.81409400	-0.45723900	0.85637700
C	-3.15214700	0.99808600	-0.96605200
C	-5.03721200	0.19645200	0.92655900
H	-3.58585500	-1.27485400	1.52900200
C	-4.37804300	1.64539700	-0.88729700
H	-2.41301000	1.30213200	-1.69701900
C	-5.32136000	1.24705600	0.05713500
H	-5.76987200	-0.11427000	1.66136100
H	-4.59721100	2.46247900	-1.56376100
H	-6.27651200	1.75485300	0.11567800
Li	2.81106000	-3.25386800	1.23295600
H	1.67250600	-1.06763600	1.79820600

Zero-point correction= 0.220398 (Hartree/Particle)
Thermal correction to Energy= 0.237097
Thermal correction to Enthalpy= 0.238041
Thermal correction to Gibbs Free Energy= 0.172578
Sum of electronic and zero-point Energies= -1133.984040
Sum of electronic and thermal Energies= -1133.967341
Sum of electronic and thermal Enthalpies= -1133.966397
Sum of electronic and thermal Free Energies= -1134.031860

Anionic Sulfone Lithiated Bridging Oxygens (8c)

S	1.43578800	1.88477600	0.02587100
O	1.75424100	2.10069900	-1.41549300
O	1.47686000	3.18536200	0.74513500
C	-0.24760000	1.41201600	-0.03073200
C	-1.31735300	0.84991800	0.01235400
C	2.34223600	0.74312900	0.78637100
C	2.35092000	-0.64545700	0.36088400
C	1.95539500	-1.08319400	-0.91898800
C	2.78425200	-1.62830000	1.27375700
C	1.98237300	-2.43305000	-1.25309000
H	1.63439900	-0.36167500	-1.66143400
C	2.81921300	-2.97139900	0.92818500
H	3.09420900	-1.32091300	2.26704800
C	2.41426200	-3.39025100	-0.33901700
H	1.66725900	-2.73672300	-2.24543100
H	3.15965300	-3.69883800	1.65688200
H	2.43748400	-4.43937200	-0.60720700
Li	1.96613800	4.11343300	-1.07974200
H	2.45546200	0.98403500	1.83589700
C	-2.59658400	0.21069200	0.03399000
C	-3.74751000	0.93746600	0.36413900
C	-2.69708500	-1.15119500	-0.27925200
C	-4.98360900	0.30482600	0.37708500
H	-3.66330700	1.98985200	0.60585700
C	-3.93856700	-1.77331100	-0.26597200
H	-1.80155400	-1.70746700	-0.52958500
C	-5.08196700	-1.04851800	0.06203500
H	-5.87198900	0.86931800	0.63285100
H	-4.01318900	-2.82602600	-0.50982200
H	-6.04851300	-1.53763300	0.07260800

Zero-point correction= 0.219506 (Hartree/Particle)
Thermal correction to Energy= 0.236503
Thermal correction to Enthalpy= 0.237448
Thermal correction to Gibbs Free Energy= 0.171357
Sum of electronic and zero-point Energies= -1133.984957
Sum of electronic and thermal Energies= -1133.967960
Sum of electronic and thermal Enthalpies= -1133.967016
Sum of electronic and thermal Free Energies= -1134.033106

Benzaldehyde

```
C                  -1.72443400    1.06258400   -0.00000300
C                  -0.35158400    1.28677200   -0.00000100
C                   0.53019300    0.20614400    0.00000100
C                   0.04140700   -1.10335500    0.00000000
C                  -1.32744800   -1.32526700   -0.00000200
C                  -2.20891200   -0.24220500   -0.00000300
H                  -2.41266100    1.89876300   -0.00000400
H                   0.03970100    2.29902600    0.00000000
H                   0.74392800   -1.92861400    0.00000100
H                  -1.71428000   -2.33702000   -0.00000200
H                  -3.27796400   -0.41946700   -0.00000500
C                   1.98690500    0.46707300    0.00000300
H                   2.27179800    1.53518000    0.00000300
O                   2.83409000   -0.39479300    0.00000500
```

```
Zero-point correction= 0.110558 (Hartree/Particle)
Thermal correction to Energy= 0.116886
Thermal correction to Enthalpy= 0.117830
Thermal correction to Gibbs Free Energy= 0.079950
Sum of electronic and zero-point Energies= -345.411667
Sum of electronic and thermal Energies= -345.405339
Sum of electronic and thermal Enthalpies= -345.404395
Sum of electronic and thermal Free Energies= -345.442275
```

Table S8. Important bond lengths and angles for lithiated sulfone **8**.

	**C5-S-C3-Ph angle**	**C3-S-O-Li angle**	**C6-Ph (Å)**	**C5-C6 (Å)**	**C5-S (Å)**	**S-O (Å)**	**O-Li (Å)**	**S-C3 (Å)**	**C3-Ph (Å)**	**C3-H (Å)**
8a	47.76	-24.38	1.430	1.209	1.745	1.495, 1.464	1.953	1.669	1.460	1.084
8b	-74.42	-30.66	1.430	1.209	1.747	1.492, 1.465	1.979	1.675	1.469	1.090
8c	55.11	-	1.430	1.209	1.749	1.492, 1.487	2.051, 2.105	1.644	1.452	1.083

Pre-Complexes of Lithiated Sulfone and Benzaldehyde:

PC_a

S	-0.46402400	-2.03829100	-0.11896000
O	-0.23259800	-2.33179800	1.32299600
O	-0.51902100	-3.21007700	-0.99914800
C	-2.09628000	-1.39908400	-0.07266000
C	-3.10541200	-0.73469000	-0.12067000
C	0.58628300	-0.90018100	-0.71994900
C	0.74350400	0.41995100	-0.13867900
C	0.52230200	0.72875100	1.21761100
C	1.20959800	1.46146600	-0.96876600
C	0.75258400	2.01214100	1.70596300
H	0.17374500	-0.04151300	1.89433500
C	1.44871400	2.73267900	-0.47185100
H	1.39605400	1.25040600	-2.01688500
C	1.22055800	3.02409200	0.87352000
H	0.56852500	2.21759400	2.75516400
H	1.81794900	3.50411600	-1.13916600
H	1.40524300	4.01826500	1.26245300
Li	1.23525500	-2.73358300	2.41901200
H	0.64795300	-0.99514400	-1.79812200
C	-4.32295200	0.01523500	-0.15938000
C	-5.33381800	-0.32537600	-1.06750300
C	-4.50624800	1.09698600	0.71208400
C	-6.51239300	0.40840100	-1.09803600
H	-5.18651200	-1.16160700	-1.73985800
C	-5.68849900	1.82434900	0.67358100
H	-3.71938800	1.35785300	1.40949400
C	-6.69251800	1.48241000	-0.22942500
H	-7.29183400	0.14182300	-1.80142100
H	-5.82606600	2.66000900	1.34894200
H	-7.61322700	2.05262300	-0.25686700
O	2.72060200	-1.75136000	1.70539800
C	3.05518800	-1.68150600	0.53110200
H	2.87732900	-2.52293600	-0.15326000
C	3.75705900	-0.52233400	-0.03609600
C	4.04586100	0.58785000	0.76045300
C	4.09610600	-0.52979400	-1.38938400
C	4.68263200	1.68581400	0.20117000
H	3.75371700	0.57941700	1.80374200
C	4.73085300	0.57271100	-1.94851600
H	3.84954400	-1.39345400	-1.99892300
C	5.02350600	1.67743900	-1.15138800
H	4.90440700	2.55380400	0.81030400
H	4.99412000	0.57487200	-2.99920100
H	5.51542300	2.53927900	-1.58745300

```
Zero-point correction=                           0.332305 (Hartree/Particle)
Thermal correction to Energy=                           0.356841
Thermal correction to Enthalpy=                         0.357785
Thermal correction to Gibbs Free Energy=                0.274196
Sum of electronic and zero-point Energies=           -1479.419768
Sum of electronic and thermal Energies=              -1479.395232
Sum of electronic and thermal Enthalpies=            -1479.394288
Sum of electronic and thermal Free Energies=         -1479.477877
```

PC_b

S	0.37086400	-0.91851200	0.17475600
O	0.62364400	-0.68449700	1.62398100
O	0.98376000	-2.12346300	-0.38839300
C	-1.35815000	-1.21492000	0.17864800
C	-2.56183800	-1.17676500	0.07239300
C	0.71842700	0.41388300	-0.74991700
C	0.17435600	1.73000800	-0.49941500
C	-0.58563500	2.08902900	0.63341300
C	0.43808000	2.74893800	-1.44148600
C	-1.04645000	3.39076100	0.80591900
H	-0.82948300	1.34547000	1.38316700
C	-0.02364900	4.04369100	-1.26053100
H	1.01632500	2.50353900	-2.32691200
C	-0.77130000	4.38145600	-0.13159000
H	-1.63182300	3.62912800	1.68744500
H	0.20088900	4.79699500	-2.00772500
H	-1.13225300	5.39270500	0.00999200
Li	1.54207500	0.49741000	2.76512800
H	1.01258000	0.12988800	-1.75220900
C	-3.98883000	-1.15670000	-0.02895900
C	-4.68644800	0.03264300	0.21989500
C	-4.69263600	-2.31724800	-0.37468600
C	-6.07181800	0.05501600	0.12613800
H	-4.13526500	0.92784300	0.48242400
C	-6.07794300	-2.28400400	-0.46692200
H	-4.14824500	-3.23367900	-0.56656400
C	-6.76937800	-1.10089500	-0.21689000
H	-6.60755800	0.97648100	0.31917500
H	-6.61903600	-3.18343400	-0.73446500
H	-7.85001600	-1.07957400	-0.29003300
O	2.77636500	1.38095600	1.58837700
C	3.14722200	1.12028800	0.45372500
H	3.02196800	1.86436200	-0.34665500

C	3.86076700	-0.11364500	0.07628500
C	4.18500400	-1.08214800	1.02843500
C	4.21737300	-0.29663300	-1.26089800
C	4.85154400	-2.23548800	0.63803900
H	3.91994800	-0.92475600	2.06714800
C	4.88247700	-1.45147000	-1.65080000
H	3.95623600	0.46241900	-1.99156500
C	5.19612900	-2.42123900	-0.70032400
H	5.10420700	-2.99102000	1.37193300
H	5.15413100	-1.59946300	-2.68870000
H	5.71336100	-3.32427500	-1.00246400

```
Zero-point correction=                           0.332978 (Hartree/Particle)
Thermal correction to Energy=                    0.357240
Thermal correction to Enthalpy=                  0.358184
Thermal correction to Gibbs Free Energy=         0.275189
Sum of electronic and zero-point Energies=          -1479.414703
Sum of electronic and thermal Energies=             -1479.390441
Sum of electronic and thermal Enthalpies=           -1479.389497
Sum of electronic and thermal Free Energies=        -1479.472492
```

PC_c

S	-0.27352200	-1.48754800	0.02540500
O	0.25245200	-1.41189900	1.39225200
O	-0.35707000	-2.85047800	-0.57112500
C	-1.96825000	-1.06105900	0.20845600
C	-3.08077800	-0.58717200	0.19487100
C	0.50524300	-0.49088100	-1.04555500
C	0.86546900	0.88689800	-0.76534900
C	1.06526400	1.41895000	0.52423400
C	1.11462800	1.74005300	-1.85997000
C	1.48448200	2.73217600	0.69590100
H	0.91573000	0.78779400	1.39020500
C	1.54589600	3.04718700	-1.67934600
H	0.97254200	1.35696400	-2.86569800
C	1.73384600	3.55934600	-0.39698600
H	1.63499500	3.10898300	1.70213100
H	1.73263500	3.67099100	-2.54685000
H	2.06983900	4.57932000	-0.25340500
Li	1.02754100	-4.15267700	-0.52606700
H	0.26463300	-0.76711700	-2.06596100
C	-4.41488700	-0.07111300	0.20431000
C	-4.78806500	0.90171800	1.14101700
C	-5.35524300	-0.53910700	-0.72307200

C	-6.08587900	1.39582400	1.14666300
H	-4.05759400	1.26081900	1.85555900
C	-6.65074400	-0.03933100	-0.70872700
H	-5.06204300	-1.29089000	-1.44558000
C	-7.01868500	0.92744500	0.22426500
H	-6.36996600	2.14785500	1.87266200
H	-7.37434400	-0.40482800	-1.42722500
H	-8.03020200	1.31521200	0.23224500
O	2.59834600	-3.06022800	-0.26155300
C	2.91452400	-2.00329600	-0.79422200
H	2.79371800	-1.87143700	-1.87910000
C	3.54737300	-0.88202800	-0.08810300
C	3.69635000	-0.89969800	1.30086500
C	3.98086600	0.21966400	-0.82849400
C	4.29230000	0.17785200	1.94046300
H	3.33282200	-1.75386600	1.85931200
C	4.57748700	1.29725600	-0.18635700
H	3.83665700	0.23269600	-1.90399000
C	4.73472900	1.27295400	1.19669200
H	4.40901000	0.17297500	3.01760800
H	4.90577500	2.15711600	-0.75735600
H	5.19415400	2.11524600	1.70122800

Zero-point correction= 0.332322 (Hartree/Particle)
Thermal correction to Energy= 0.356808
Thermal correction to Enthalpy= 0.357752
Thermal correction to Gibbs Free Energy= 0.274369
Sum of electronic and zero-point Energies= -1479.418236
Sum of electronic and thermal Energies= -1479.393751
Sum of electronic and thermal Enthalpies= -1479.392806
Sum of electronic and thermal Free Energies= -1479.476189

PC_d

S	0.43899200	-1.47303100	-0.54799600
O	0.18665500	-1.29541800	-1.98371300
O	0.39964300	-2.87558700	-0.04586900
C	2.13422700	-1.05593800	-0.38259200
C	3.22288100	-0.58243300	-0.15308400
C	-0.54021100	-0.55017400	0.41305700
C	-0.86602600	0.84299400	0.18934800
C	-0.64647400	1.54438600	-1.01294500
C	-1.51802000	1.53582400	1.23042000
C	-1.06336200	2.86353600	-1.15543700

H	-0.16216500	1.04706600	-1.84407600
C	-1.94014500	2.84724800	1.07533200
H	-1.70550500	1.01866700	2.16604300
C	-1.71786500	3.52772600	-0.12122700
H	-0.87864000	3.37519100	-2.09395600
H	-2.45034000	3.34216200	1.89478400
H	-2.04706200	4.55257600	-0.24360500
Li	-1.00925800	-4.04715300	0.41215400
H	-0.59431800	-0.95020700	1.41834000
C	4.53264600	-0.06153700	0.09001700
C	5.05181400	0.94628100	-0.73330200
C	5.30259600	-0.55873700	1.14977100
C	6.32549700	1.44612400	-0.49645300
H	4.45230300	1.32765300	-1.55076900
C	6.57591600	-0.05340900	1.37723800
H	4.89677800	-1.33759700	1.78363900
C	7.08947000	0.94822200	0.55662200
H	6.72276100	2.22529300	-1.13549200
H	7.16840700	-0.44175400	2.19668700
H	8.08313500	1.34004800	0.73733400
O	-2.67952100	-3.04571300	0.35312700
C	-2.79793200	-2.06018600	-0.36672200
H	-2.28805200	-2.01910900	-1.34087000
C	-3.65756800	-0.90924200	-0.06172400
C	-4.35651100	-0.82672000	1.14536400
C	-3.73704200	0.12676600	-0.99455100
C	-5.13907000	0.28834600	1.40945100
H	-4.27474300	-1.63628600	1.86159500
C	-4.51985600	1.24287800	-0.72723500
H	-3.17021200	0.05983400	-1.91753400
C	-5.22098700	1.32036400	0.47286700
H	-5.68409000	0.36068900	2.34297400
H	-4.57364900	2.05344800	-1.44354700
H	-5.82879600	2.19210200	0.68627100

Zero-point correction= 0.332114 (Hartree/Particle)
Thermal correction to Energy= 0.356788
Thermal correction to Enthalpy= 0.357732
Thermal correction to Gibbs Free Energy= 0.273902
Sum of electronic and zero-point Energies= -1479.418302
Sum of electronic and thermal Energies= -1479.393628
Sum of electronic and thermal Enthalpies= -1479.392684
Sum of electronic and thermal Free Energies= -1479.476513

PC_e

S	0.35147000	-2.15470400	-0.35884300
O	0.58819100	-3.31289800	-1.23398100
O	0.09507000	-2.57334600	1.04864700
C	1.85793300	-1.28757900	-0.34675200
C	2.89352400	-0.67372900	-0.25928000
C	-0.72443200	-0.96963400	-0.84660800
C	-2.15754700	-1.03620800	-0.63818100
C	-2.79775000	-1.70170700	0.42743700
C	-2.97868000	-0.28869300	-1.51142500
C	-4.17669100	-1.61748000	0.59770000
H	-2.21361200	-2.28701400	1.12417900
C	-4.34944300	-0.20496700	-1.32840700
H	-2.51516400	0.24160800	-2.33707000
C	-4.96796700	-0.87045700	-0.26960300
H	-4.63581000	-2.14514600	1.42719500
H	-4.94164300	0.38757900	-2.01773400
H	-6.03998100	-0.80781100	-0.12645300
Li	0.06077200	-1.69068300	2.72807400
H	-0.36175100	-0.50006300	-1.75599100
C	4.11755200	0.06353600	-0.17963700
C	5.32404100	-0.59921000	0.07753200
C	4.10722600	1.45287300	-0.35805700
C	6.50610400	0.12534100	0.15579600
H	5.32456900	-1.67378900	0.21276000
C	5.29524100	2.16767000	-0.28181600
H	3.16982200	1.95827200	-0.55749400
C	6.49398500	1.50655100	-0.02435800
H	7.43826500	-0.38874200	0.35550000
H	5.28573700	3.24151400	-0.42280400
H	7.41876000	2.06761900	0.03552800
O	-0.62343300	0.03621200	2.23959000
C	-0.40906400	0.76082100	1.27426100
H	0.59459200	0.82108900	0.83054300
C	-1.42029400	1.67342800	0.71721100
C	-2.70704600	1.71761000	1.25587500
C	-1.08889500	2.46565900	-0.38262200
C	-3.65789500	2.56089300	0.69699600
H	-2.94802100	1.07990700	2.09793700
C	-2.04216700	3.30654600	-0.94299900
H	-0.08925800	2.40554500	-0.80267800
C	-3.32567800	3.35309100	-0.40077400
H	-4.66059700	2.59510800	1.10597200
H	-1.79127100	3.92012300	-1.79983000
H	-4.07143000	4.00563400	-0.84006500

Zero-point correction= 0.331976 (Hartree/Particle)

Thermal correction to Energy= 0.356538
Thermal correction to Enthalpy= 0.357482
Thermal correction to Gibbs Free Energy= 0.274390
Sum of electronic and zero-point Energies= -1479.411559
Sum of electronic and thermal Energies= -1479.386997
Sum of electronic and thermal Enthalpies= -1479.386053
Sum of electronic and thermal Free Energies= -1479.469145

PC_f

S	0.19012800	1.49426400	1.95137700
O	0.15389400	0.77127100	3.25519200
O	0.56385700	2.91113400	2.01426000
C	1.58251100	0.79320800	1.13829100
C	2.46740600	0.29847300	0.47983300
C	-1.20437200	1.14643500	1.13885700
C	-1.55288800	1.64349600	-0.16907100
C	-0.66112700	2.32912500	-1.01981600
C	-2.85154700	1.39567300	-0.66092200
C	-1.05697300	2.74254000	-2.28670400
H	0.34815200	2.54507800	-0.68668800
C	-3.23930500	1.81618600	-1.92431800
H	-3.55294800	0.85159800	-0.03499000
C	-2.34633300	2.49451700	-2.75342400
H	-0.34600800	3.26633400	-2.91634900
H	-4.24674200	1.60641700	-2.26766000
H	-2.64842000	2.82217400	-3.74063000
Li	0.40767300	-1.10707200	3.53612600
H	-1.85809500	0.49236100	1.69635500
C	3.54831400	-0.28579900	-0.25469500
C	3.62823900	-1.67688400	-0.39930600
C	4.53431100	0.53076400	-0.82358600
C	4.68393500	-2.24047400	-1.10392400
H	2.86476900	-2.30460100	0.04484000
C	5.58565700	-0.04208500	-1.52698300
H	4.46819400	1.60553900	-0.70712800
C	5.66277400	-1.42581400	-1.66817400
H	4.74305300	-3.31653500	-1.21278300
H	6.34678600	0.59222900	-1.96463900
H	6.48506600	-1.86876300	-2.21700000
O	-0.26264000	-1.89410000	1.89859400
C	-0.55609400	-1.64026800	0.73968700
H	0.19890300	-1.22378200	0.05521100
C	-1.87563900	-1.90068400	0.14871500
C	-2.93714800	-2.36395800	0.93467500

C	-2.06140800	-1.65039600	-1.21087300
C	-4.17626700	-2.57900600	0.35350400
H	-2.77487700	-2.54390400	1.99115500
C	-3.30310400	-1.88062500	-1.79459600
H	-1.23782700	-1.26516300	-1.80387400
C	-4.35636400	-2.34131700	-1.01257700
H	-5.00513500	-2.93242000	0.95461300
H	-3.45150900	-1.68532500	-2.84944500
H	-5.32678500	-2.51332400	-1.46332400

Zero-point correction= 0.331597 (Hartree/Particle)
Thermal correction to Energy= 0.356553
Thermal correction to Enthalpy= 0.357497
Thermal correction to Gibbs Free Energy= 0.271994
Sum of electronic and zero-point Energies= -1479.414971
Sum of electronic and thermal Energies= -1479.390016
Sum of electronic and thermal Enthalpies= -1479.389071
Sum of electronic and thermal Free Energies= -1479.474574

PC_g

S	2.39430200	-1.82562800	-0.44613900
O	2.83060700	-2.14554000	-1.80985800
O	2.61887700	-2.90017500	0.56573100
C	0.65228100	-1.73626600	-0.63005100
C	-0.49884600	-1.42462900	-0.82727000
C	2.96762000	-0.40377800	0.17930400
C	2.62925100	0.90099800	-0.33405000
C	1.93471500	1.12948000	-1.54185500
C	2.96372900	2.03821500	0.43587500
C	1.56533800	2.41415900	-1.92426300
H	1.68287000	0.29608300	-2.18783500
C	2.60231800	3.31749500	0.03893800
H	3.51307500	1.90028800	1.36231200
C	1.88566800	3.52095200	-1.14055400
H	1.02310600	2.55008000	-2.85406100
H	2.87591700	4.16331900	0.66046900
H	1.59601600	4.51845800	-1.44723100
Li	1.81154400	-2.93245000	2.30353900
H	3.29124300	-0.52302600	1.20384300
C	-1.86268700	-1.06626900	-1.06543000
C	-2.90113400	-1.94687900	-0.73545700
C	-2.15714400	0.18195600	-1.62574600
C	-4.21890100	-1.57619100	-0.96345200
H	-2.66525100	-2.91045100	-0.29999400

C	-3.47960900	0.54183000	-1.85335300
H	-1.34658500	0.86114800	-1.86506400
C	-4.51072400	-0.33242500	-1.52109600
H	-5.02060200	-2.25764400	-0.70529000
H	-3.70515600	1.51098400	-2.28226100
H	-5.54106100	-0.04658800	-1.69643200
O	0.68810300	-1.37279100	2.54188400
C	0.58036100	-0.19393600	2.24600600
H	1.46836200	0.45291300	2.20659900
C	-0.69006500	0.46317300	1.92614800
C	-1.91131100	-0.18684700	2.13051400
C	-0.65253900	1.75676300	1.39681400
C	-3.09405400	0.46709000	1.82287400
H	-1.91515100	-1.19543700	2.52815200
C	-1.84072100	2.40227900	1.07599900
H	0.30525800	2.23971100	1.22332600
C	-3.05635600	1.75959400	1.29697600
H	-4.04554600	-0.02725400	1.97677300
H	-1.82029100	3.39974300	0.65397800
H	-3.98350600	2.26254600	1.04704400

Zero-point correction= 0.332626 (Hartree/Particle)
Thermal correction to Energy= 0.356856
Thermal correction to Enthalpy= 0.357800
Thermal correction to Gibbs Free Energy= 0.277639
Sum of electronic and zero-point Energies= -1479.419222
Sum of electronic and thermal Energies= -1479.394992
Sum of electronic and thermal Enthalpies= -1479.394048
Sum of electronic and thermal Free Energies= -1479.474209

PC_h

S	1.02069500	-1.61642400	-0.40629400
O	1.26282700	-1.20413100	-1.82242800
O	1.26519200	-3.06027500	-0.24464000
C	-0.66162400	-1.36837400	-0.09562400
C	-1.84431200	-1.23857000	0.10068500
C	1.72606500	-0.54737300	0.67908500
C	3.15556800	-0.33404200	0.70751200
C	4.05377900	-0.76707300	-0.29080100
C	3.69860900	0.41784000	1.77345600
C	5.40875400	-0.46281500	-0.21869000
H	3.69559700	-1.36356500	-1.12210400
C	5.05150100	0.71648300	1.83642000
H	3.03453700	0.76549000	2.55896600

C	5.92453500	0.28174400	0.83878300
H	6.07023000	-0.81977700	-1.00068900
H	5.42886200	1.29440700	2.67306500
H	6.98111400	0.51411300	0.88862300
Li	1.94419800	0.23050600	-2.83388100
H	1.14597400	-0.46502200	1.59010500
C	-3.24273000	-1.03443100	0.31636600
C	-3.72060800	-0.75332200	1.60137500
C	-4.12461700	-1.05461800	-0.77016800
C	-5.06965200	-0.48514000	1.79171100
H	-3.02953500	-0.73183400	2.43532900
C	-5.47253700	-0.78711800	-0.56945400
H	-3.74434700	-1.26649300	-1.76211600
C	-5.94592900	-0.49777400	0.70837700
H	-5.43767100	-0.26242900	2.78586600
H	-6.15349300	-0.79993600	-1.41174900
H	-6.99717700	-0.28454900	0.86044300
O	1.97435000	1.66854000	-1.58068300
C	1.37501800	1.84920100	-0.52799200
H	1.93577400	2.08389500	0.38749700
C	-0.09422000	1.93548400	-0.42278400
C	-0.90575000	1.82819100	-1.55380600
C	-0.66891300	2.15261200	0.83114600
C	-2.28554200	1.92497200	-1.42660900
H	-0.45125500	1.67910100	-2.52626500
C	-2.04826700	2.24896300	0.95734900
H	-0.02831600	2.23023500	1.70431200
C	-2.85609400	2.13208800	-0.17222800
H	-2.91921200	1.83421700	-2.30097100
H	-2.49694000	2.40458100	1.93117600
H	-3.93500500	2.18419300	-0.07179100

Zero-point correction= 0.332106 (Hartree/Particle)
Thermal correction to Energy= 0.356535
Thermal correction to Enthalpy= 0.357479
Thermal correction to Gibbs Free Energy= 0.276344
Sum of electronic and zero-point Energies= -1479.411701
Sum of electronic and thermal Energies= -1479.387272
Sum of electronic and thermal Enthalpies= -1479.386328
Sum of electronic and thermal Free Energies= -1479.467463

Transition States for Benzaldehyde Addition to the Lithiated Sulfone:

$PC_a^‡$

S	-0.32358500	-1.92855100	0.14443100
O	-0.16481300	-2.20032600	1.59625500
O	-0.24511300	-3.09791500	-0.73282400
C	-1.96104300	-1.34052600	0.04365500
C	-3.00331800	-0.75074800	-0.11574500
C	0.74344600	-0.73799100	-0.38218500
C	0.74258500	0.59635900	0.19782700
C	0.38979200	0.88508200	1.52892400
C	1.20199700	1.66302000	-0.59952100
C	0.48098600	2.18314900	2.02444600
H	0.03795300	0.09367500	2.17870700
C	1.29883200	2.95161000	-0.09666600
H	1.49685000	1.46077700	-1.62467300
C	0.93594500	3.22504400	1.22196200
H	0.19551700	2.37773700	3.05239800
H	1.66083000	3.74830900	-0.73713800
H	1.00723300	4.23196600	1.61523400
Li	1.37578500	-2.44015700	2.68659100
H	0.84057900	-0.80973500	-1.46092000
C	-4.25590200	-0.08210800	-0.28702100
C	-5.30144000	-0.71099400	-0.97490200
C	-4.43585300	1.20666300	0.23164400
C	-6.51341100	-0.05350400	-1.13868300
H	-5.15468100	-1.70724500	-1.37350500
C	-5.65214200	1.85493700	0.06256000
H	-3.62121900	1.68745800	0.75976800
C	-6.69091600	1.22766600	-0.62138400
H	-7.32065600	-0.54174300	-1.67080300
H	-5.78906400	2.85157000	0.46395300
H	-7.63807300	1.73708600	-0.75171200
O	2.74520800	-1.62742500	1.67753400
C	2.83950700	-1.61978100	0.44237700
H	2.59158200	-2.51450500	-0.14437800
C	3.61384000	-0.57978400	-0.27396100
C	4.12194700	0.51776100	0.42019500
C	3.83116300	-0.69806800	-1.64779400
C	4.84875700	1.49071700	-0.25684900
H	3.93540800	0.59767200	1.48496900
C	4.55365400	0.27607400	-2.32486900
H	3.42304800	-1.55088900	-2.18202900
C	5.06349900	1.37179300	-1.62777200
H	5.24186300	2.34533600	0.28108500
H	4.72182400	0.18466000	-3.39124700
H	5.62719600	2.13224900	-2.15552300

```
 Zero-point correction=                           0.332156 (Hartree/Particle)
 Thermal correction to Energy=                           0.355897
 Thermal correction to Enthalpy=                         0.356841
 Thermal correction to Gibbs Free Energy=                0.273705
 Sum of electronic and zero-point Energies=                 -1479.418691
 Sum of electronic and thermal Energies=                    -1479.394949
 Sum of electronic and thermal Enthalpies=                  -1479.394005
 Sum of electronic and thermal Free Energies=               -1479.477142
```

Imaginary Frequency -116.23 cm^{-1}

$PC_b^‡$

S	0.45861800	-0.88054800	0.44854000
O	0.68026600	-0.56173800	1.88321400
O	1.11314800	-2.08901400	-0.04741500
C	-1.25906800	-1.18226300	0.38881100
C	-2.45182600	-1.19004200	0.19611900
C	0.82315300	0.43986900	-0.53100700
C	0.11346100	1.70339600	-0.39400200
C	-0.60966500	2.10090000	0.74636600
C	0.20162900	2.62442900	-1.45746200
C	-1.21666800	3.35202900	0.80747700
H	-0.71548100	1.42733500	1.58810900
C	-0.39971300	3.87240400	-1.38645700
H	0.75049500	2.34237900	-2.35060600
C	-1.11692600	4.24899300	-0.25107800
H	-1.77354800	3.62518700	1.69701400
H	-0.31214600	4.55463000	-2.22459400
H	-1.58983300	5.22187100	-0.19584600
Li	1.77449800	0.68656900	2.81514800
H	1.04506200	0.09307300	-1.53477300
C	-3.86777300	-1.21479900	-0.00287500
C	-4.61217100	-0.04032600	0.16802400
C	-4.51113800	-2.40294700	-0.37134900
C	-5.98703700	-0.06032300	-0.02545900
H	-4.10535900	0.87535100	0.44866000
C	-5.88648000	-2.41160600	-0.56284500
H	-3.92899500	-3.30665100	-0.50312700
C	-6.62548200	-1.24343900	-0.39040400
H	-6.56070800	0.84883000	0.10697000
H	-6.38247000	-3.33146700	-0.84750100
H	-7.69818900	-1.25489300	-0.54129900
O	2.79512000	1.48703700	1.46021000
C	2.97481600	1.14708700	0.28423700

H	2.82361900	1.87815100	-0.52196400
C	3.75093400	-0.06055700	-0.09846200
C	4.24187900	-0.93715500	0.86852300
C	4.01918700	-0.29482800	-1.44836800
C	4.97807200	-2.05230500	0.48492900
H	4.05363700	-0.73927300	1.91718300
C	4.75453200	-1.40794400	-1.83216000
H	3.63813800	0.39542900	-2.19509600
C	5.23086200	-2.29131100	-0.86384600
H	5.35742700	-2.73476400	1.23611700
H	4.95775300	-1.59004400	-2.88057900
H	5.80347100	-3.16193400	-1.16109600

Zero-point correction= 0.332844 (Hartree/Particle)
Thermal correction to Energy= 0.356269
Thermal correction to Enthalpy= 0.357214
Thermal correction to Gibbs Free Energy= 0.276344
Sum of electronic and zero-point Energies= -1479.413623
Sum of electronic and thermal Energies= -1479.390197
Sum of electronic and thermal Enthalpies= -1479.389253
Sum of electronic and thermal Free Energies= -1479.470123

Imaginary Frequency -127.84 cm^{-1}

$PC_c^{‡}$

S	-0.07261200	-1.71012100	0.15229300
O	0.48048100	-1.56997400	1.49871100
O	-0.10267500	-3.08477500	-0.40472200
C	-1.74900100	-1.26053300	0.29261700
C	-2.84781900	-0.75878300	0.25990800
C	0.68937100	-0.70476000	-0.98203600
C	0.70555700	0.74369800	-0.76811000
C	0.86462400	1.34542800	0.49275300
C	0.65578900	1.58387400	-1.89333900
C	0.95560900	2.72667700	0.61291500
H	0.93958000	0.72365400	1.37594200
C	0.76359800	2.96384700	-1.76844300
H	0.53695600	1.14110000	-2.87718700
C	0.91078800	3.54719800	-0.51219600
H	1.07890100	3.16551300	1.59696100
H	0.72623900	3.58554300	-2.65599100
H	0.99122400	4.62300300	-0.41200100
Li	1.48462600	-4.13938700	-0.63616200
H	0.36580400	-1.02564800	-1.96943300

C	-4.16195500	-0.19570300	0.25053600
C	-4.44703100	0.92624500	1.03954300
C	-5.16397500	-0.76160900	-0.54814100
C	-5.72353400	1.47230600	1.02670300
H	-3.66640900	1.35893200	1.65321100
C	-6.43737700	-0.20816200	-0.55263500
H	-4.93605600	-1.62846900	-1.15609100
C	-6.71898900	0.90731800	0.23279600
H	-5.94188900	2.33997900	1.63709800
H	-7.21103500	-0.64750200	-1.17035900
H	-7.71374100	1.33627600	0.22587800
O	2.76963600	-2.78873500	-0.61016400
C	2.81810000	-1.60388600	-1.00060000
H	2.76330300	-1.38085600	-2.07473700
C	3.44891500	-0.53528300	-0.18652900
C	3.62938400	-0.69654200	1.18752800
C	3.86713200	0.64156500	-0.80810800
C	4.22267300	0.31538200	1.93202700
H	3.28239600	-1.60868800	1.65757300
C	4.46640700	1.65219400	-0.06395500
H	3.71544200	0.76587300	-1.87576000
C	4.64272100	1.49003900	1.30756700
H	4.35758700	0.19326700	3.00049200
H	4.78929700	2.56512900	-0.55046600
H	5.10485100	2.27780400	1.89125700

Zero-point correction= 0.332788 (Hartree/Particle)
Thermal correction to Energy= 0.356146
Thermal correction to Enthalpy= 0.357090
Thermal correction to Gibbs Free Energy= 0.276726
Sum of electronic and zero-point Energies= -1479.415598
Sum of electronic and thermal Energies= -1479.392240
Sum of electronic and thermal Enthalpies= -1479.391296
Sum of electronic and thermal Free Energies= -1479.471660

Imaginary Frequency -136.72 cm^{-1}

$PC_d^‡$

S	0.31750100	-1.75508600	-0.61184400
O	0.13933700	-1.71521800	-2.06584700
O	0.20634100	-3.09366800	0.02068100

C	1.97783300	-1.28823900	-0.35926900
C	3.02488100	-0.74215400	-0.10108300
C	-0.71944900	-0.70508300	0.19758500
C	-0.75581200	0.71954200	-0.11124200
C	-0.52391600	1.26181700	-1.38837500
C	-1.14107000	1.60375900	0.91331600
C	-0.66457300	2.62693600	-1.61665900
H	-0.24634300	0.60896500	-2.20672300
C	-1.29519200	2.96183300	0.67450100
H	-1.33448000	1.20624000	1.90439600
C	-1.05458800	3.48762100	-0.59384600
H	-0.47637800	3.01871500	-2.61020900
H	-1.60289900	3.61460900	1.48396300
H	-1.17047600	4.54833500	-0.78134300
Li	-1.37567900	-4.14890200	0.29199300
H	-0.75631400	-0.97905400	1.24802900
C	4.28483300	-0.12771300	0.18040900
C	4.77062800	0.88570700	-0.65623100
C	5.03322800	-0.53328800	1.29287600
C	5.99268500	1.48348500	-0.37874500
H	4.18577600	1.19520400	-1.51372700
C	6.25493900	0.07000100	1.56028100
H	4.65154000	-1.31656100	1.93609200
C	6.73598900	1.07749500	0.72722500
H	6.36544700	2.26728100	-1.02672000
H	6.83230800	-0.24565700	2.42059900
H	7.68912100	1.54643300	0.93986000
O	-2.75593200	-2.85365400	0.32062600
C	-2.78888300	-1.79136400	-0.32116800
H	-2.47496200	-1.76603100	-1.37405600
C	-3.57913400	-0.62005200	0.12152600
C	-4.05281100	-0.53319300	1.43163900
C	-3.84136500	0.40885900	-0.78367900
C	-4.78300900	0.57877300	1.83173900
H	-3.83808800	-1.33941200	2.12391700
C	-4.57711700	1.51898600	-0.38411200
H	-3.45903900	0.34002000	-1.79694800
C	-5.04693600	1.60467100	0.92377600
H	-5.14849900	0.64970000	2.84948000
H	-4.77800400	2.31792400	-1.08797200
H	-5.61663400	2.47131100	1.23842800

Zero-point correction= 0.332366 (Hartree/Particle)
Thermal correction to Energy= 0.356005
Thermal correction to Enthalpy= 0.356949
Thermal correction to Gibbs Free Energy= 0.275441
Sum of electronic and zero-point Energies= -1479.416936

Sum of electronic and thermal Energies= -1479.393297
Sum of electronic and thermal Enthalpies= -1479.392353
Sum of electronic and thermal Free Energies= -1479.473860

Imaginary Frequency -115.53 cm^{-1}

$PC_e^{‡}$

S	0.33693100	-2.09554500	-0.33189500
O	0.50986600	-3.22645600	-1.25453400
O	0.10796900	-2.55376700	1.06666900
C	1.85540500	-1.25456500	-0.33301200
C	2.90504700	-0.66456500	-0.25423800
C	-0.72886300	-0.85988200	-0.74640200
C	-2.16907600	-1.00411500	-0.60954700
C	-2.81012300	-1.70688500	0.42958800
C	-2.98947500	-0.28757800	-1.50571300
C	-4.19638500	-1.68845000	0.55372500
H	-2.22113200	-2.26627500	1.14374200
C	-4.36855900	-0.26612800	-1.36767800
H	-2.52176000	0.26936500	-2.31113800
C	-4.98933100	-0.96873300	-0.33499500
H	-4.65961500	-2.24270700	1.36310100
H	-4.96469300	0.30417000	-2.07196000
H	-6.06731700	-0.95577100	-0.22775500
Li	0.05631500	-1.66761600	2.74924700
H	-0.36086200	-0.36197000	-1.63972100
C	4.14561500	0.04500900	-0.18365100
C	5.34543700	-0.65138300	0.00691500
C	4.15731200	1.44049700	-0.30362500
C	6.54371200	0.04680000	0.07741500
H	5.32827600	-1.73048000	0.09742900
C	5.36138000	2.12860600	-0.23538500
H	3.22462000	1.97168900	-0.45116500
C	6.55381900	1.43437700	-0.04429400
H	7.47103800	-0.49282600	0.22545400
H	5.36926500	3.20744300	-0.33072200
H	7.49118400	1.97476100	0.00962700
O	-0.63358700	0.03446200	2.23275400
C	-0.41928400	0.73922000	1.24527900
H	0.59444900	0.83570400	0.83361500
C	-1.42940400	1.66772700	0.70075700
C	-2.71554700	1.70505200	1.23976900
C	-1.09705900	2.48886200	-0.37749300
C	-3.66467300	2.56616500	0.70271000
H	-2.95786300	1.04881600	2.06711800

C	-2.04680400	3.34828500	-0.91515500
H	-0.09752600	2.43772000	-0.79941400
C	-3.33132500	3.38610800	-0.37330400
H	-4.66660300	2.59360400	1.11451100
H	-1.79205200	3.98444600	-1.75436400
H	-4.07415900	4.05342400	-0.79489300

Zero-point correction= 0.331680 (Hartree/Particle)
Thermal correction to Energy= 0.355468
Thermal correction to Enthalpy= 0.356412
Thermal correction to Gibbs Free Energy= 0.274627
Sum of electronic and zero-point Energies= -1479.411791
Sum of electronic and thermal Energies= -1479.388003
Sum of electronic and thermal Enthalpies= -1479.387059
Sum of electronic and thermal Free Energies= -1479.468844

Imaginary Frequency -89.00 cm^{-1}

$PC_f^{‡}$

S	0.18415300	1.41540600	1.96013900
O	0.15579200	0.69515000	3.26053600
O	0.43021000	2.85699100	2.04625600
C	1.59118900	0.80077200	1.13895300
C	2.49842800	0.32217500	0.50227000
C	-1.15359600	0.90485000	1.07448800
C	-1.53241400	1.52867000	-0.17899000
C	-0.62533600	2.17740200	-1.03940200
C	-2.86717800	1.41213000	-0.61194700
C	-1.04162800	2.68455700	-2.26471800
H	0.41300300	2.29437600	-0.74946800
C	-3.27578800	1.92025800	-1.83714900
H	-3.58197500	0.90038100	0.02550800
C	-2.36699100	2.56146300	-2.67683700
H	-0.32046400	3.18206800	-2.90363700
H	-4.31144900	1.81081300	-2.13979800
H	-2.68527800	2.95947000	-3.63268100
Li	0.41099100	-1.20649700	3.49351600
H	-1.92116000	0.55517300	1.75438900
C	3.58030500	-0.25332900	-0.23597200
C	3.64128100	-1.64209600	-0.40939400
C	4.57364600	0.56527800	-0.78746400
C	4.68896200	-2.20250500	-1.12773700
H	2.86830400	-2.26813400	0.02043100
C	5.61657400	-0.00518700	-1.50506100

H	4.51934300	1.63800900	-0.64873900
C	5.67607400	-1.38644400	-1.67564700
H	4.73510900	-3.27637500	-1.26091700
H	6.38439700	0.62873200	-1.93122600
H	6.49191500	-1.82697100	-2.23594100
O	-0.25975300	-1.85228200	1.86082900
C	-0.58689200	-1.45361000	0.73540500
H	0.17103600	-1.07110300	0.03771600
C	-1.89689700	-1.77242300	0.12714100
C	-2.93935900	-2.28882600	0.90247000
C	-2.08280700	-1.56175700	-1.23870600
C	-4.15791900	-2.58850000	0.31091400
H	-2.77943600	-2.44694600	1.96296000
C	-3.30097600	-1.87677100	-1.83392800
H	-1.27508800	-1.14274100	-1.83095600
C	-4.33791200	-2.38617400	-1.05973900
H	-4.96959300	-2.98370700	0.91014300
H	-3.44324600	-1.71290100	-2.89536800
H	-5.28943400	-2.62561700	-1.51985500

Zero-point correction= 0.331816 (Hartree/Particle)
Thermal correction to Energy= 0.355615
Thermal correction to Enthalpy= 0.356560
Thermal correction to Gibbs Free Energy= 0.274724
Sum of electronic and zero-point Energies= -1479.413223
Sum of electronic and thermal Energies= -1479.389423
Sum of electronic and thermal Enthalpies= -1479.388479
Sum of electronic and thermal Free Energies= -1479.470314

Imaginary Frequency -157.31 cm^{-1}

$PC_g^‡$

S	1.47505300	-1.44630800	-1.54030700
O	1.74182300	-1.14788900	-2.95125300
O	1.53652000	-2.89421800	-1.20458200
C	-0.16790000	-0.96000400	-1.30162800
C	-1.28345600	-0.51723100	-1.18281000
C	2.38760900	-0.62428000	-0.37942000
C	2.52497300	0.82056700	-0.35920000
C	1.65246700	1.72131600	-0.99912100
C	3.54200000	1.37063400	0.44873800
C	1.77251100	3.09330900	-0.80514700
H	0.87517800	1.35553200	-1.65844000
C	3.65628000	2.74057600	0.63656800
H	4.24410300	0.70252900	0.93786400

C	2.76437900	3.61729100	0.01926700
H	1.08175800	3.75946500	-1.31043100
H	4.44772100	3.12704500	1.26902400
H	2.85055400	4.68666800	0.16781100
Li	1.45988500	-3.86779400	0.43083700
H	3.25729800	-1.23187300	-0.15240500
C	-2.59568600	0.02109000	-1.00909800
C	-3.69019000	-0.82905500	-0.81858100
C	-2.77150700	1.40953700	-0.98042200
C	-4.95002800	-0.28954800	-0.59353300
H	-3.54276600	-1.90211300	-0.83559900
C	-4.03485000	1.93934200	-0.75559800
H	-1.91455300	2.05880100	-1.11729100
C	-5.12360300	1.09229800	-0.55868100
H	-5.79688200	-0.94774500	-0.44207000
H	-4.16983900	3.01376300	-0.72892900
H	-6.10743200	1.50910600	-0.37942600
O	1.87648000	-2.60267400	1.75345200
C	1.70180800	-1.37473600	1.79401200
H	2.53557600	-0.70840900	2.05146900
C	0.35726600	-0.76533500	1.91931900
C	-0.77519200	-1.57797800	2.01157000
C	0.22359300	0.62313300	2.00389400
C	-2.03012400	-1.00843000	2.18235600
H	-0.65956600	-2.65453200	1.96131600
C	-1.03638700	1.19192400	2.15462100
H	1.10585000	1.25271700	1.94290100
C	-2.16265000	0.37727000	2.24732500
H	-2.90787400	-1.63999000	2.25606800
H	-1.14058600	2.26963500	2.20384400
H	-3.14551500	0.82181500	2.35917700

Zero-point correction=	0.332249 (Hartree/Particle)
Thermal correction to Energy=	0.355777
Thermal correction to Enthalpy=	0.356721
Thermal correction to Gibbs Free Energy=	0.277567
Sum of electronic and zero-point Energies=	-1479.414145
Sum of electronic and thermal Energies=	-1479.390617
Sum of electronic and thermal Enthalpies=	-1479.389673
Sum of electronic and thermal Free Energies=	-1479.468827

Imaginary Frequency -149.58 cm^{-1}

$PC_h^‡$

S	1.01958000	-1.57212600	-0.43722400
O	1.25307500	-1.18434900	-1.85972400
O	1.29669600	-3.00338100	-0.23426500
C	-0.66490400	-1.34589900	-0.12788500
C	-1.84702500	-1.22834200	0.07816700
C	1.71237000	-0.45958100	0.62502900
C	3.15229800	-0.30566100	0.68860200
C	4.05027900	-0.74638100	-0.30473900
C	3.69909500	0.40392200	1.77961700
C	5.41388600	-0.49273100	-0.20194100
H	3.68605900	-1.30901900	-1.15671200
C	5.06015400	0.65484500	1.87257200
H	3.03280800	0.75643500	2.56102000
C	5.93463400	0.21009200	0.88098600
H	6.07695400	-0.85460300	-0.98017000
H	5.44240600	1.20143700	2.72771000
H	6.99760200	0.40406100	0.95438500
Li	1.93111800	0.26206800	-2.86894600
H	1.13447000	-0.39199200	1.53998900
C	-3.24556500	-1.03919300	0.30587100
C	-3.71658600	-0.78371900	1.59868200
C	-4.13434100	-1.04957200	-0.77506800
C	-5.06673500	-0.53183100	1.80265800
H	-3.01981300	-0.76956900	2.42801600
C	-5.48330500	-0.79876900	-0.56059200
H	-3.75886600	-1.24131200	-1.77290200
C	-5.95028100	-0.53545300	0.72521100
H	-5.42998900	-0.32910100	2.80280300
H	-6.17013500	-0.80425300	-1.39816300
H	-7.00247100	-0.33521500	0.88807300
O	1.96359700	1.66548900	-1.59621100
C	1.37823100	1.81299700	-0.52506800
H	1.94654000	2.07651900	0.37719000
C	-0.09308900	1.91260200	-0.40741200
C	-0.91224100	1.82918800	-1.53412600
C	-0.66028100	2.12716900	0.85050900
C	-2.29089500	1.94380100	-1.39954500
H	-0.46502800	1.68470200	-2.51061300
C	-2.03733200	2.24135400	0.98458900
H	-0.01495900	2.18863000	1.72166200
C	-2.85351000	2.14613600	-0.14158000
H	-2.92902900	1.87111500	-2.27248400
H	-2.47830800	2.39523800	1.96232300
H	-3.93096700	2.21282800	-0.03532200

Zero-point correction= 0.332298 (Hartree/Particle)
Thermal correction to Energy= 0.355738
Thermal correction to Enthalpy= 0.356682

Thermal correction to Gibbs Free Energy=	0.278080
Sum of electronic and zero-point Energies=	-1479.411498
Sum of electronic and thermal Energies=	-1479.388059
Sum of electronic and thermal Enthalpies=	-1479.387114
Sum of electronic and thermal Free Energies=	-1479.465717

Imaginary Frequency -83.73 cm^{-1}

Products of Benzaldehyde Addition to the Lithiated Sulfone:

9a

S	-0.05549000	-2.22541400	0.14840300
O	0.11989400	-2.58577200	1.56620300
O	0.13259500	-3.28824700	-0.83080700
C	-1.64671800	-1.59588000	-0.00045900
C	-2.64936600	-0.94655200	-0.16952900
C	0.97768000	-0.80370400	-0.30927800
C	0.62008900	0.40355700	0.51101400
C	0.98398500	0.52704600	1.85468400
C	-0.08771700	1.44223800	-0.09957100
C	0.64056600	1.67057700	2.56900000
H	1.55074800	-0.26617500	2.32288400
C	-0.42870600	2.58570900	0.61515800
H	-0.37348700	1.35358300	-1.14288300
C	-0.06756300	2.70025600	1.95454600
H	0.92924800	1.75842300	3.60984800
H	-0.97659000	3.38300700	0.12667200
H	-0.33341500	3.58836900	2.51571100
Li	2.01727000	-2.90211900	2.09307200
H	0.67915800	-0.64369000	-1.34973200
C	-3.83453700	-0.16998900	-0.34205400
C	-4.83026700	-0.58376000	-1.23503600
C	-3.98158600	1.02055000	0.38196600
C	-5.96905500	0.19306500	-1.39733600
H	-4.70359800	-1.50494700	-1.79002600
C	-5.12388600	1.78989000	0.20835300
H	-3.19925300	1.32950300	1.06542400
C	-6.11620500	1.37731700	-0.67828400
H	-6.74198700	-0.12462300	-2.08606900
H	-5.24063300	2.71139900	0.76513500
H	-7.00642600	1.98049000	-0.80985700
O	2.83316800	-1.82406500	0.92763200
C	2.48226800	-1.29927800	-0.26671700
H	2.53996600	-2.03113400	-1.09587900
C	3.31112200	-0.09030400	-0.70666900

C	4.04580900	0.62957600	0.23173300
C	3.32201700	0.32329500	-2.03958500
C	4.77491300	1.75260400	-0.15333600
H	4.03724500	0.28834500	1.26014700
C	4.04837300	1.44496900	-2.42869500
H	2.76417600	-0.24156100	-2.78194100
C	4.77601300	2.16539700	-1.48333300
H	5.34456900	2.30616200	0.58478900
H	4.05299700	1.75322600	-3.46797700
H	5.34467500	3.03778900	-1.78357000

Zero-point correction= 0.335610 (Hartree/Particle)
Thermal correction to Energy= 0.358938
Thermal correction to Enthalpy= 0.359883
Thermal correction to Gibbs Free Energy= 0.279118
Sum of electronic and zero-point Energies= -1479.434499
Sum of electronic and thermal Energies= -1479.411171
Sum of electronic and thermal Enthalpies= -1479.410227
Sum of electronic and thermal Free Energies= -1479.490991

9b

S	0.56386500	-0.26672200	1.38636100
O	0.52862900	0.71488600	2.48454600
O	1.34183300	-1.47924900	1.59261100
C	-1.07407300	-0.71414300	1.10530500
C	-2.19369100	-0.96762100	0.73568900
C	1.02752000	0.52160700	-0.19217800
C	0.09219400	1.66488800	-0.48477600
C	0.31505200	2.93675900	0.04881700
C	-1.00749400	1.46026400	-1.32175600
C	-0.55535100	3.98102300	-0.24455800
H	1.18162800	3.09729800	0.67914900
C	-1.87847000	2.50509200	-1.61358400
H	-1.18047900	0.47778700	-1.74905300
C	-1.65624700	3.76732200	-1.07068500
H	-0.37299200	4.96493100	0.17125700
H	-2.72602400	2.33297400	-2.26668200
H	-2.33297400	4.58296100	-1.29643200
Li	2.24815600	1.68527800	2.71807700
H	0.85602000	-0.28890100	-0.90568200
C	-3.52676200	-1.23497900	0.29965100
C	-4.37445900	-0.16234200	-0.00652700
C	-3.97664800	-2.55372200	0.16226200
C	-5.66644900	-0.41445700	-0.44717300
H	-4.01114100	0.85267800	0.10220400

C	-5.27154500	-2.79277400	-0.27695500
H	-3.31131700	-3.37448900	0.39968900
C	-6.11500200	-1.72655000	-0.58165700
H	-6.32403600	0.41252200	-0.68484500
H	-5.62297400	-3.81159200	-0.38289100
H	-7.12426600	-1.91878600	-0.92521900
O	2.90378400	1.42487600	1.09266200
C	2.56162600	0.90587000	-0.10760100
H	2.65179900	1.64147300	-0.93304600
C	3.41402100	-0.29328900	-0.54437200
C	4.33291500	-0.86287900	0.33076300
C	3.30933100	-0.80518000	-1.83947200
C	5.12293400	-1.93762200	-0.07237900
H	4.41327500	-0.45005000	1.32870100
C	4.09482500	-1.87824100	-2.24774600
H	2.61244700	-0.35417300	-2.54168200
C	5.00496700	-2.45100800	-1.36084200
H	5.83280200	-2.37537900	0.62065000
H	4.00270100	-2.26364600	-3.25679900
H	5.62009500	-3.28610400	-1.67521500

Zero-point correction=	0.335568 (Hartree/Particle)
Thermal correction to Energy=	0.358864
Thermal correction to Enthalpy=	0.359808
Thermal correction to Gibbs Free Energy=	0.279074
Sum of electronic and zero-point Energies=	-1479.431282
Sum of electronic and thermal Energies=	-1479.407986
Sum of electronic and thermal Enthalpies=	-1479.407042
Sum of electronic and thermal Free Energies=	-1479.487776

9c

S	-0.39284800	-1.86900900	-0.19983300
O	-1.04118700	-1.62279000	-1.48016700
O	-0.46266700	-3.24085900	0.33461100
C	1.28293900	-1.50595200	-0.35330300
C	2.40646300	-1.06800600	-0.35792100
C	-0.91412500	-0.67026900	1.07046100
C	-0.33218600	0.69087300	0.79976500
C	-0.52163900	1.35737300	-0.41542700
C	0.40609800	1.31357900	1.80759600
C	0.02382000	2.62080000	-0.61266600
H	-1.09798300	0.88671600	-1.20397400
C	0.93545900	2.58685900	1.61621600
H	0.56583700	0.79882700	2.74923400
C	0.74780900	3.24187800	0.40307400
H	-0.12442500	3.12538600	-1.56006300

H	1.50008400	3.06025800	2.41066500
H	1.16482700	4.22996500	0.24770500
Li	-2.20308300	-3.71059000	1.16044600
H	-0.44552100	-1.10184000	1.95961700
C	3.71779000	-0.50419400	-0.37219400
C	3.84898300	0.89085100	-0.36164300
C	4.85256000	-1.32326300	-0.38612400
C	5.11596300	1.45829900	-0.36502400
H	2.95841700	1.50980200	-0.34726200
C	6.11382900	-0.74312400	-0.39229600
H	4.73726900	-2.39996500	-0.39243600
C	6.24630500	0.64376200	-0.38147200
H	5.22200700	2.53609900	-0.35582100
H	6.99451100	-1.37318800	-0.40447500
H	7.23313200	1.09072500	-0.38534300
O	-2.85888400	-2.08025100	1.38151600
C	-2.48093200	-0.78562900	1.31313300
H	-2.56789900	-0.25421500	2.28312300
C	-3.28890800	0.05383100	0.32166200
C	-3.84491400	-0.52697800	-0.81643500
C	-3.51068900	1.41049200	0.56024200
C	-4.57910700	0.24020000	-1.71633300
H	-3.69099600	-1.58507100	-0.98741500
C	-4.24660600	2.18326700	-0.33406000
H	-3.09526500	1.86924800	1.45325500
C	-4.77805300	1.59945200	-1.48097800
H	-5.00139200	-0.22240100	-2.60142300
H	-4.40825400	3.23647100	-0.13437500
H	-5.35277700	2.19567800	-2.18035200

Zero-point correction= 0.335297 (Hartree/Particle)
Thermal correction to Energy= 0.358753
Thermal correction to Enthalpy= 0.359697
Thermal correction to Gibbs Free Energy= 0.279526
Sum of electronic and zero-point Energies= -1479.431991
Sum of electronic and thermal Energies= -1479.408536
Sum of electronic and thermal Enthalpies= -1479.407592
Sum of electronic and thermal Free Energies= -1479.487763

9d

S	-0.03553200	-2.19905100	-0.45680100
O	-0.18081300	-2.40646400	-1.89215700
O	-0.25817600	-3.36238700	0.42005300
C	1.56092200	-1.64836300	-0.14097400
C	2.57905500	-1.04422800	0.08928900

C	-1.05933000	-0.79097900	0.08463700
C	-0.46398600	0.51163200	-0.36766700
C	-0.30809800	0.81054200	-1.72370000
C	-0.10080100	1.46265200	0.58726800
C	0.20143500	2.04532900	-2.11323800
H	-0.57929200	0.07576600	-2.47340500
C	0.39812800	2.70091100	0.19656900
H	-0.21931900	1.23382700	1.64111100
C	0.55096600	2.99456900	-1.15590900
H	0.32117500	2.26765000	-3.16693000
H	0.67013500	3.43336500	0.94732100
H	0.94191800	3.95750900	-1.46271400
Li	-2.18351600	-3.80758900	0.66325700
H	-1.03542400	-0.87865600	1.17488000
C	3.76877700	-0.29276300	0.32741600
C	3.82951500	1.02890600	-0.13432500
C	4.85079100	-0.85483300	1.01444700
C	4.97353400	1.78097200	0.09491600
H	2.97979300	1.44896100	-0.66129000
C	5.99032200	-0.09291700	1.23413400
H	4.79049200	-1.87694400	1.36694100
C	6.05225500	1.22159800	0.77655600
H	5.02439200	2.80327600	-0.25903800
H	6.83069500	-0.52430800	1.76366000
H	6.94370400	1.81164000	0.95208800
O	-2.91369400	-2.33150900	-0.00483800
C	-2.52526300	-1.09835100	-0.40352000
H	-2.49851300	-0.98271300	-1.50388500
C	-3.38946200	0.04012200	0.14264400
C	-3.90270200	-0.04895700	1.43590300
C	-3.66118600	1.17929100	-0.61493300
C	-4.66481100	0.98666400	1.96906000
H	-3.70744300	-0.94703200	2.01149600
C	-4.42567400	2.21720500	-0.08711000
H	-3.27175200	1.25426300	-1.62565100
C	-4.92668900	2.12481500	1.20881600
H	-5.05899000	0.90580500	2.97585400
H	-4.63401800	3.09479000	-0.68848200
H	-5.52321500	2.93037000	1.62094800

Zero-point correction= 0.335295 (Hartree/Particle)
Thermal correction to Energy= 0.358806
Thermal correction to Enthalpy= 0.359750
Thermal correction to Gibbs Free Energy= 0.279059
Sum of electronic and zero-point Energies= -1479.437028
Sum of electronic and thermal Energies= -1479.413517
Sum of electronic and thermal Enthalpies= -1479.412573
Sum of electronic and thermal Free Energies= -1479.493264

9e

S	0.19188900	-1.93244100	0.09546100
O	0.08031700	-2.96040300	-0.93070200
O	-0.08591500	-2.36081300	1.47671700
C	1.79269100	-1.31121900	0.06129800
C	2.87924600	-0.79505900	-0.02423400
C	-0.83446400	-0.50072400	-0.35667800
C	-2.27033400	-0.95883900	-0.30091500
C	-3.01239300	-0.91377700	0.88280500
C	-2.87721600	-1.42615100	-1.46888000
C	-4.34010900	-1.32701400	0.88992000
H	-2.54556400	-0.53177200	1.78110800
C	-4.20459800	-1.84214300	-1.45885800
H	-2.30583800	-1.46250200	-2.39023500
C	-4.93844400	-1.79435300	-0.27762900
H	-4.91050200	-1.28202800	1.81025000
H	-4.66379600	-2.20011000	-2.37251900
H	-5.97290500	-2.11683400	-0.26699400
Li	-0.13059100	-0.95914900	2.86124700
H	-0.54294000	-0.34354200	-1.39946800
C	4.16475400	-0.17690500	-0.09081800
C	5.16333000	-0.71159400	-0.91387200
C	4.41465200	0.97263400	0.66961600
C	6.40615000	-0.09590200	-0.97003400
H	4.95754100	-1.59969800	-1.49835100
C	5.66090200	1.58009600	0.60360000
H	3.63232200	1.37672600	1.30075600
C	6.65529800	1.04722100	-0.21371800
H	7.18093900	-0.50760500	-1.60476900
H	5.85682400	2.46930900	1.18979700
H	7.62670800	1.52430000	-0.26186300
O	-0.38196300	0.48179200	1.84215600
C	-0.42320500	0.74662700	0.51616500
H	0.56625300	1.03823600	0.11050000
C	-1.37414300	1.88012300	0.11553800
C	-2.27797200	2.39559900	1.03925900
C	-1.35039100	2.41106200	-1.17628700
C	-3.15463200	3.41732500	0.67784600
H	-2.27528700	1.98529700	2.04187800
C	-2.22504300	3.42881500	-1.54254100
H	-0.63541400	2.03235800	-1.90187800
C	-3.13432100	3.93385600	-0.61434900
H	-3.85417800	3.81150000	1.40655600
H	-2.19491200	3.83222900	-2.54828400
H	-3.81576700	4.72796300	-0.89637600

```
Zero-point correction=                           0.335538 (Hartree/Particle)
Thermal correction to Energy=                          0.358984
Thermal correction to Enthalpy=                        0.359929
Thermal correction to Gibbs Free Energy=               0.278713
Sum of electronic and zero-point Energies=             -1479.433071
Sum of electronic and thermal Energies=                -1479.409625
Sum of electronic and thermal Enthalpies=              -1479.408681
Sum of electronic and thermal Free Energies=           -1479.489896
```

9f

S	-0.18828900	-1.36217900	1.85220400
O	-0.14058000	-0.89743900	3.24964700
O	-0.16301400	-2.80569500	1.66067700
C	-1.63472600	-0.75413000	1.15207600
C	-2.58443600	-0.29343500	0.56890400
C	1.15167100	-0.56347500	0.90897400
C	1.45265700	-1.33118400	-0.34603900
C	0.55050900	-1.36543000	-1.41278900
C	2.68508500	-1.97150800	-0.47703700
C	0.87978400	-2.03082800	-2.58868700
H	-0.41039500	-0.86963600	-1.33108900
C	3.01842000	-2.62857300	-1.65710100
H	3.39100200	-1.94517300	0.34610100
C	2.11546900	-2.66018700	-2.71552500
H	0.17224800	-2.05272100	-3.40897000
H	3.98183500	-3.11578200	-1.74840400
H	2.37266300	-3.17272900	-3.63479300
Li	0.07749500	1.04598100	3.54506200
H	1.97360300	-0.65309100	1.62895400
C	-3.70497000	0.27711400	-0.10730900
C	-3.76503800	1.66558900	-0.28353700
C	-4.72952700	-0.54334800	-0.59502500
C	-4.84920600	2.22568300	-0.94474800
H	-2.96416200	2.28759000	0.09791900
C	-5.80959700	0.02911500	-1.25269300
H	-4.67103400	-1.61542400	-0.45374400
C	-5.87006900	1.40991800	-1.42789600
H	-4.89825300	3.29860500	-1.08302900
H	-6.60460400	-0.60216500	-1.62971500
H	-6.71495400	1.85141900	-1.94251000
O	0.47511600	1.54815300	1.89020400
C	0.84443400	0.97215600	0.72496700
H	0.05909000	1.02612400	-0.05455000
C	2.11324200	1.56855700	0.10758000
C	3.16194600	1.95706800	0.94065200

C	2.25678100	1.71740700	-1.27157200
C	4.33837500	2.47404300	0.40623100
H	3.03712500	1.86103100	2.01377300
C	3.43126600	2.23695000	-1.81167100
H	1.44343600	1.42405100	-1.92822700
C	4.47708900	2.61366600	-0.97347100
H	5.14652100	2.77274700	1.06440800
H	3.52777200	2.35146500	-2.88533300
H	5.39110700	3.01943800	-1.39117500

Zero-point correction= 0.335284 (Hartree/Particle)
Thermal correction to Energy= 0.358834
Thermal correction to Enthalpy= 0.359779
Thermal correction to Gibbs Free Energy= 0.277957
Sum of electronic and zero-point Energies= -1479.434666
Sum of electronic and thermal Energies= -1479.411116
Sum of electronic and thermal Enthalpies= -1479.410172
Sum of electronic and thermal Free Energies= -1479.491993

9g

S	1.49660800	-1.02095900	-1.59131900
O	2.22768400	-0.33608500	-2.64961100
O	1.36199600	-2.48080500	-1.72785100
C	-0.09743700	-0.39104900	-1.54603000
C	-1.22526000	-0.00128100	-1.37501400
C	2.27148300	-0.62447300	0.00136100
C	2.40936500	0.86984700	0.15694000
C	1.32515300	1.71963500	0.39622500
C	3.69073900	1.42361300	0.07873400
C	1.52321600	3.08805300	0.55229800
H	0.32414200	1.31802800	0.46728100
C	3.88980200	2.78972700	0.24323400
H	4.53983100	0.77648700	-0.11257100
C	2.80368600	3.62747900	0.47818300
H	0.67157500	3.73271100	0.73582800
H	4.89130000	3.19846100	0.18246500
H	2.95410100	4.69326700	0.60229300
Li	1.92359900	-3.81876800	-0.40476100
H	3.26441800	-1.04181000	-0.20517200
C	-2.55044600	0.46248500	-1.12448300
C	-3.63100700	-0.42077500	-1.23669300
C	-2.75659100	1.78534200	-0.71482700
C	-4.91105300	0.02171500	-0.93492400
H	-3.45583400	-1.44259500	-1.55014500
C	-4.04104700	2.21522700	-0.41038900
H	-1.91280700	2.46037500	-0.63479800

C	-5.11598200	1.33570500	-0.51841800
H	-5.74911900	-0.65892100	-1.02003500
H	-4.20389300	3.23658600	-0.08941600
H	-6.11655800	1.67563700	-0.27970200
O	2.08061900	-2.79966200	1.04620100
C	1.77693100	-1.49653000	1.23777600
H	2.37212500	-1.02942800	2.04816400
C	0.31111600	-1.25282500	1.61451200
C	-0.69336300	-2.08465500	1.11771700
C	-0.04709700	-0.23316000	2.49679900
C	-2.02611400	-1.87430700	1.45494300
H	-0.42150300	-2.90076100	0.45831300
C	-1.38125700	-0.01282100	2.83516600
H	0.72640100	0.40024900	2.92058500
C	-2.37691700	-0.82970600	2.30784200
H	-2.79470700	-2.52432400	1.05150200
H	-1.63971600	0.78877500	3.51780700
H	-3.41619000	-0.66213300	2.56735700

Zero-point correction= 0.336051 (Hartree/Particle)
Thermal correction to Energy= 0.359062
Thermal correction to Enthalpy= 0.360006
Thermal correction to Gibbs Free Energy= 0.282037
Sum of electronic and zero-point Energies= -1479.429848
Sum of electronic and thermal Energies= -1479.406838
Sum of electronic and thermal Enthalpies= -1479.405894
Sum of electronic and thermal Free Energies= -1479.483862

9h

S	-1.23556200	-1.54830700	0.04262300
O	-1.72510900	-1.98264300	1.36126100
O	-1.67772700	-2.34670600	-1.09447100
C	0.47473400	-1.57748600	0.07120600
C	1.66297200	-1.39989500	-0.03720500
C	-1.65305400	0.19863300	-0.27900700
C	-3.16258000	0.25725400	-0.34036000
C	-3.91745200	0.62078100	0.77788000
C	-3.81571500	-0.04191200	-1.53848500
C	-5.30455700	0.68423600	0.69302800
H	-3.40685100	0.86180200	1.70309400
C	-5.20264500	0.01925200	-1.61998700
H	-3.23284700	-0.32427000	-2.40818000
C	-5.95011400	0.38115000	-0.50285300
H	-5.88255400	0.97254600	1.56312700
H	-5.69769400	-0.21142500	-2.55568800
H	-7.03077000	0.43095800	-0.56516100

Li	-1.53404200	-0.75999700	2.88562800
H	-1.24388600	0.33181900	-1.28372600
C	3.06532600	-1.16901800	-0.14939700
C	3.61721000	-0.86990700	-1.40021200
C	3.87173900	-1.18622100	0.99494600
C	4.97166400	-0.58323600	-1.50006800
H	2.97969300	-0.85149900	-2.27562300
C	5.22500500	-0.90190700	0.88290700
H	3.42880100	-1.40911200	1.95798100
C	5.77391600	-0.59672000	-0.36135900
H	5.40109200	-0.34519100	-2.46541300
H	5.85171100	-0.91136400	1.76612900
H	6.83019400	-0.36992600	-0.44336600
O	-1.05492200	0.71870000	2.03516900
C	-0.99796100	1.18120900	0.76483400
H	-1.61827000	2.09118800	0.61971400
C	0.41085100	1.61346100	0.32297800
C	1.45386600	1.59401600	1.24488700
C	0.66358600	2.10456300	-0.96120300
C	2.72657000	2.04008200	0.89299000
H	1.24631900	1.21591200	2.23857600
C	1.93521400	2.54155600	-1.32048100
H	-0.13893200	2.16438400	-1.69104600
C	2.97458800	2.51016000	-0.39223900
H	3.52895200	2.00955000	1.62218900
H	2.11329000	2.91357700	-2.32325800
H	3.96596000	2.84882400	-0.67067200

```
Zero-point correction=                           0.335565 (Hartree/Particle)
Thermal correction to Energy=                           0.358754
Thermal correction to Enthalpy=                         0.359698
Thermal correction to Gibbs Free Energy=                0.281090
Sum of electronic and zero-point Energies=           -1479.433718
Sum of electronic and thermal Energies=              -1479.410529
Sum of electronic and thermal Enthalpies=            -1479.409585
Sum of electronic and thermal Free Energies=         -1479.488193
```

Cyclization to Form Oxathiin Ring:

9_{cis}

S	-0.19222300	-1.93205000	-0.09581800
O	0.08543400	-2.36051500	-1.47707400

O	-0.08090700	-2.96004000	0.93034600
C	-1.79288800	-1.31047600	-0.06163400
C	-2.87949500	-0.79445100	0.02405500
C	0.83447000	-0.50058900	0.35639100
C	0.42373700	0.74684200	-0.51656500
H	-0.56578200	1.03867000	-0.11120400
O	0.38285400	0.48203000	-1.84257300
C	2.27024000	-0.95906300	0.30093000
C	3.01260000	-0.91412700	-0.88260600
C	2.87674500	-1.42652900	1.46903300
C	4.34023200	-1.32764500	-0.88940600
H	2.54609300	-0.53197500	-1.78101400
C	4.20404000	-1.84279900	1.45932400
H	2.30513800	-1.46277100	2.39025000
C	4.93818600	-1.79513700	0.27827400
H	4.91086000	-1.28274700	-1.80959500
H	4.66294100	-2.20087700	2.37309100
H	5.97258300	-2.11783000	0.26788600
C	1.37480300	1.88014100	-0.11567300
C	1.35092200	2.41101200	1.17617800
C	2.27886000	2.39552700	-1.03922000
C	2.22566200	3.42862100	1.54262200
H	0.63576800	2.03238500	1.90163500
C	3.15561200	3.41710800	-0.67761500
H	2.27626600	1.98526500	-2.04185500
C	3.13516400	3.93357700	0.61460200
H	2.19542700	3.83199100	2.54837900
H	3.85533300	3.81121700	-1.40619100
H	3.81667700	4.72757300	0.89677900
C	-4.16511800	-0.17655200	0.09081800
C	-4.41554100	0.97266900	-0.66992100
C	-5.16331000	-0.71122400	0.91435000
C	-5.66192900	1.57983000	-0.60373100
H	-3.63351100	1.37675300	-1.30143600
C	-6.40627300	-0.09583700	0.97068200
H	-4.95711700	-1.59908400	1.49905800
C	-6.65594300	1.04697000	0.21406000
H	-5.85826000	2.46879400	-1.19016800
H	-7.18076500	-0.50753200	1.60578400
H	-7.62746400	1.52381100	0.26233300
Li	0.13163600	-0.95885200	-2.86167900
H	0.54278900	-0.34329000	1.39912000

Zero-point correction= 0.335535 (Hartree/Particle)
Thermal correction to Energy= 0.358984
Thermal correction to Enthalpy= 0.359928
Thermal correction to Gibbs Free Energy= 0.278693
Sum of electronic and zero-point Energies= -1479.433074

Sum of electronic and thermal Energies= -1479.409625
Sum of electronic and thermal Enthalpies= -1479.408681
Sum of electronic and thermal Free Energies= -1479.489916

9$_{trans}$

S	0.19127900	1.34830300	1.85728800
O	0.14918500	0.87439500	3.25175500
O	0.16682000	2.79304900	1.67509000
C	1.63478000	0.74332400	1.14828500
C	2.58663400	0.28686300	0.56535000
C	-1.15290600	0.55771200	0.91362200
C	-0.85066800	-0.97785800	0.72183500
H	-0.06504100	-1.03037500	-0.05750500
O	-0.48415400	-1.56114700	1.88426100
C	-1.45441500	1.33162200	-0.33750400
C	-0.55452900	1.36756800	-1.40610200
C	-2.68543300	1.97568300	-0.46340900
C	-0.88453300	2.03843200	-2.57868800
H	0.40517000	0.86882100	-1.32850700
C	-3.01958000	2.63811800	-1.64021200
H	-3.38977100	1.94784800	0.36103000
C	-2.11881200	2.67150700	-2.70045600
H	-0.17868400	2.06161700	-3.40038900
H	-3.98196900	3.12806700	-1.72759700
H	-2.37663000	3.18822900	-3.61720700
C	-2.12084100	-1.56705700	0.10059700
C	-2.26064900	-1.71563000	-1.27888800
C	-3.17439200	-1.94946100	0.93041100
C	-3.43597800	-2.22950300	-1.82263300
H	-1.44366500	-1.42657800	-1.93294200
C	-4.35167300	-2.46062000	0.39239500
H	-3.05286200	-1.85331500	2.00391300
C	-4.48643800	-2.60045600	-0.98771500
H	-3.52950600	-2.34409100	-2.89655100
H	-5.16361000	-2.75461100	1.04801700
H	-5.40110700	-3.00184700	-1.40821700
C	3.71029700	-0.27702700	-0.11132300
C	4.73035400	0.54990300	-0.59751700
C	3.77867600	-1.66491000	-0.28891100
C	5.81416200	-0.01547200	-1.25514800
H	4.66549000	1.62144600	-0.45506600
C	4.86655100	-2.21792100	-0.95003900
H	2.98135700	-2.29210200	0.09144500
C	5.88287100	-1.39569200	-1.43174500
H	6.60564900	0.62088500	-1.63101900
H	4.92201700	-3.29038200	-1.08935700
H	6.73063100	-1.83169600	-1.94631400

Li	-0.06350800	-1.07210000	3.53728800
H	-1.97306700	0.64641100	1.63572600

Zero-point correction= 0.335206 (Hartree/Particle)
Thermal correction to Energy= 0.358807
Thermal correction to Enthalpy= 0.359751
Thermal correction to Gibbs Free Energy= 0.277355
Sum of electronic and zero-point Energies= -1479.434745
Sum of electronic and thermal Energies= -1479.411143
Sum of electronic and thermal Enthalpies= -1479.410199
Sum of electronic and thermal Free Energies= -1479.492595

$9_{cis}^{‡}$

S	0.08250300	-2.30673300	0.22718900
O	0.21629900	-2.41247600	-1.24732300
O	0.65456900	-3.41229500	0.98991700
C	-1.58123500	-2.08007500	0.57483300
C	-2.22532700	-1.08309000	0.18177300
C	0.90395000	-0.75034700	0.76045400
C	-0.03106900	0.46643700	0.41139100
H	-0.68674600	0.56332600	1.29457600
O	-0.78949500	0.24358300	-0.71901700
C	2.29047900	-0.78406400	0.17187300
C	2.56409000	-0.24530500	-1.08813400
C	3.32434300	-1.38295400	0.89295100
C	3.84880400	-0.30403300	-1.61398600
H	1.77650100	0.24884200	-1.64357400
C	4.61187200	-1.44137100	0.36765800
H	3.11800200	-1.80420700	1.87093200
C	4.87591600	-0.90314200	-0.88757700
H	4.05065800	0.12326900	-2.58901400
H	5.40649800	-1.90440800	0.94028400
H	5.87819900	-0.94420000	-1.29719300
C	0.75886700	1.77126600	0.36900200
C	1.61168800	2.12290300	1.41865900
C	0.60409300	2.64992900	-0.69845100
C	2.30812600	3.32528900	1.39172700
H	1.73834300	1.45138700	2.26296800
C	1.30110300	3.85741100	-0.72796400
H	-0.06479700	2.37189000	-1.50405700
C	2.15710300	4.19730800	0.31381900
H	2.96975000	3.58358400	2.21046800
H	1.17407100	4.53216100	-1.56700000
H	2.70095000	5.13430000	0.29119300
C	-3.44306500	-0.32754300	0.02868000
C	-3.46847100	1.06315700	-0.11942200

C	-4.64593200	-1.04789000	0.09143100
C	-4.68930000	1.72164900	-0.19862700
H	-2.53109000	1.59732500	-0.18879500
C	-5.85859100	-0.37779700	0.00912800
H	-4.61582900	-2.12436300	0.20770700
C	-5.88302600	1.00738700	-0.13569200
H	-4.70783800	2.79870800	-0.31309000
H	-6.78427600	-0.93826300	0.05590800
H	-6.83085200	1.52800500	-0.20188500
Li	-0.51166400	-0.83602300	-2.19251400
H	0.96590400	-0.88280700	1.84333200

Zero-point correction= 0.335365 (Hartree/Particle)
Thermal correction to Energy= 0.357550
Thermal correction to Enthalpy= 0.358494
Thermal correction to Gibbs Free Energy= 0.282190
Sum of electronic and zero-point Energies= -1479.408970
Sum of electronic and thermal Energies= -1479.386784
Sum of electronic and thermal Enthalpies= -1479.385840
Sum of electronic and thermal Free Energies= -1479.462144

Imaginary Frequency -349.12 cm^{-1}

$9_{trans}^{‡}$

S	-0.00793400	-1.87535800	1.46915200
O	-0.35549800	-1.37817800	2.82593800
O	0.66437000	-3.16950900	1.43069900
C	-1.44181700	-1.87601600	0.52574800
C	-2.08674100	-0.83830300	0.26128300
C	1.06204100	-0.58554800	0.73229800
C	0.18854300	0.63111300	0.24980600
H	-0.08999600	0.40575600	-0.79410600
O	-0.93029500	0.81314400	1.02982000
C	1.97420600	-1.14607500	-0.31876000
C	1.45932500	-1.66748000	-1.50913200
C	3.35532200	-1.10500500	-0.13431900
C	2.31559600	-2.13956700	-2.49651100
H	0.38515200	-1.71155300	-1.66171700
C	4.21386300	-1.56735000	-1.12783600
H	3.76018500	-0.69913300	0.78657200
C	3.69554900	-2.08567100	-2.31011200
H	1.90661200	-2.54738400	-3.41317300
H	5.28578300	-1.52359000	-0.97633700
H	4.36221400	-2.44854700	-3.08328100
C	1.11088300	1.84331100	0.20429100

C	1.89299400	2.09934900	-0.92326700
C	1.20850500	2.69556800	1.30303400
C	2.76423000	3.18510800	-0.94912700
H	1.81873200	1.44353700	-1.78569700
C	2.07683800	3.78352200	1.27949000
H	0.58929800	2.50797100	2.17269400
C	2.85884100	4.03002700	0.15364400
H	3.36418800	3.37444800	-1.83172600
H	2.14113400	4.44166500	2.13844600
H	3.53340300	4.87789900	0.13382400
C	-3.21826500	-0.14372900	-0.29936800
C	-4.41103100	-0.87176900	-0.42565000
C	-3.15402100	1.17631400	-0.75748900
C	-5.52400600	-0.28089800	-1.00835900
H	-4.45100300	-1.89343300	-0.06819200
C	-4.27379200	1.75518700	-1.34074800
H	-2.23494600	1.72987100	-0.62819300
C	-5.45771800	1.03235100	-1.46709100
H	-6.44355300	-0.84569300	-1.10120400
H	-4.22265000	2.77759300	-1.69504500
H	-6.32810300	1.49247500	-1.91937200
Li	-1.18828200	0.41195000	2.81976900
H	1.63062800	-0.28230200	1.61815300

Zero-point correction= 0.335417 (Hartree/Particle)
Thermal correction to Energy= 0.357572
Thermal correction to Enthalpy= 0.358516
Thermal correction to Gibbs Free Energy= 0.282148
Sum of electronic and zero-point Energies= -1479.413202
Sum of electronic and thermal Energies= -1479.391047
Sum of electronic and thermal Enthalpies= -1479.390102
Sum of electronic and thermal Free Energies= -1479.466471

Imaginary Frequency -344.85 cm^{-1}

10_{cis}

S	0.02765600	-2.23717500	0.23857200
O	0.32940000	-1.96813700	1.68556700
O	0.58398700	-3.49733200	-0.26072700
C	-1.67086300	-2.07870000	-0.06170400
C	-1.99952900	-0.78512800	0.09642700
C	0.83598100	-0.88093900	-0.71526500
C	-0.05479900	0.40083800	-0.54825600
H	-0.59479800	0.52002600	-1.49319900
O	-1.05039300	0.21213100	0.46655900
C	2.30018700	-0.82979900	-0.37642300

C	2.77720800	-0.30608700	0.82913200
C	3.21550300	-1.34184500	-1.29767200
C	4.14041700	-0.28799800	1.09732300
H	2.08808500	0.09765300	1.55953000
C	4.58111300	-1.32369100	-1.03040500
H	2.85538400	-1.75379000	-2.23434400
C	5.04695700	-0.79399100	0.16799000
H	4.49682300	0.12458900	2.03370000
H	5.27732400	-1.72124000	-1.75912500
H	6.10961300	-0.77444300	0.37862700
C	0.69888200	1.68347700	-0.28608100
C	1.67145200	2.08252700	-1.20669800
C	0.43705000	2.48766900	0.81913600
C	2.39063700	3.25308500	-1.00963200
H	1.87407300	1.46645100	-2.07716300
C	1.16400600	3.66127600	1.01957100
H	-0.33863500	2.21130100	1.52199700
C	2.14450600	4.04350800	0.11256700
H	3.14456600	3.54904100	-1.72927400
H	0.95598200	4.27715400	1.88660300
H	2.70861100	4.95481700	0.27089100
C	-3.35792400	-0.21628700	-0.05098900
C	-3.57125600	1.16550600	-0.01763800
C	-4.45553500	-1.06709900	-0.23647700
C	-4.85456900	1.68498300	-0.17177400
H	-2.73115000	1.83327000	0.12772200
C	-5.73232100	-0.54630300	-0.38627200
H	-4.28732100	-2.13691400	-0.26091600
C	-5.93812200	0.83364300	-0.35472500
H	-5.00469500	2.75796100	-0.14664800
H	-6.57250600	-1.21618200	-0.52690600
H	-6.93649400	1.23819200	-0.47143100
Li	-0.57845500	-0.46715000	2.45331700
H	0.73018800	-1.23679300	-1.74117000

Zero-point correction= 0.338355 (Hartree/Particle)
Thermal correction to Energy= 0.360199
Thermal correction to Enthalpy= 0.361143
Thermal correction to Gibbs Free Energy= 0.286731
Sum of electronic and zero-point Energies= -1479.444323
Sum of electronic and thermal Energies= -1479.422479
Sum of electronic and thermal Enthalpies= -1479.421535
Sum of electronic and thermal Free Energies= -1479.495948

10_{trans}

S	0.17723500	-1.87567700	1.40215900

O	-0.06735400	-1.42355800	2.81689200
O	0.94680400	-3.11685200	1.29386600
C	-1.33237200	-1.94580900	0.55881800
C	-1.78342200	-0.69307400	0.37370200
C	1.18722500	-0.48712800	0.69848100
C	0.19449300	0.53089600	0.08681800
H	0.00823700	0.25000300	-0.95493100
O	-1.06040700	0.46443300	0.77794000
C	2.23882800	-0.95519300	-0.26190300
C	1.88627100	-1.61672500	-1.44153200
C	3.58437400	-0.70980700	0.00662600
C	2.86672000	-2.01963300	-2.33918500
H	0.84208500	-1.83172400	-1.65072300
C	4.56716600	-1.10777600	-0.89593400
H	3.86325000	-0.19720800	0.92095600
C	4.20993900	-1.76151800	-2.07033200
H	2.58413700	-2.53734300	-3.24791300
H	5.60963000	-0.90700700	-0.67961200
H	4.97336500	-2.07342400	-2.77298200
C	0.74686000	1.93531200	0.12245800
C	1.73572100	2.28874600	-0.79940000
C	0.31605700	2.87550700	1.05624900
C	2.29594600	3.56086000	-0.77659500
H	2.06269300	1.56725500	-1.54119700
C	0.87517200	4.15155700	1.07373100
H	-0.46838300	2.62582200	1.75918200
C	1.86826600	4.49588200	0.16293800
H	3.05994800	3.82442300	-1.49824100
H	0.52882300	4.87785700	1.79951000
H	2.30071400	5.48911500	0.17812700
C	-3.06787700	-0.33571600	-0.27058100
C	-4.00707500	-1.33503300	-0.55726800
C	-3.36164300	0.98757600	-0.61504800
C	-5.20651400	-1.01724200	-1.17731700
H	-3.77753100	-2.35842700	-0.28631800
C	-4.56557900	1.30236400	-1.24110800
H	-2.64437700	1.76862900	-0.39533800
C	-5.49106600	0.30424900	-1.52382800
H	-5.92447000	-1.80027200	-1.39151900
H	-4.77821900	2.33123900	-1.50704100
H	-6.42824300	0.55048300	-2.00877000
Li	-1.12939200	0.13617900	3.03976000
H	1.64347500	-0.05908100	1.59465400

```
Zero-point correction=                          0.337755 (Hartree/Particle)
Thermal correction to Energy=                        0.359876
Thermal correction to Enthalpy=                      0.360820
Thermal correction to Gibbs Free Energy=             0.284540
```

Sum of electronic and zero-point Energies= -1479.447633
Sum of electronic and thermal Energies= -1479.425513
Sum of electronic and thermal Enthalpies= -1479.424568
Sum of electronic and thermal Free Energies= -1479.500849

10cis'

S	0.02765600	-2.23717500	0.23857200
O	0.32940000	-1.96813700	1.68556700
O	0.58398700	-3.49733200	-0.26072700
C	-1.67086300	-2.07870000	-0.06170400
C	-1.99952900	-0.78512800	0.09642700
C	0.83598100	-0.88093900	-0.71526500
C	-0.05479900	0.40083800	-0.54825600
H	-0.59479800	0.52002600	-1.49319900
O	-1.05039300	0.21213100	0.46655900
C	2.30018700	-0.82979900	-0.37642300
C	2.77720800	-0.30608700	0.82913200
C	3.21550300	-1.34184500	-1.29767200
C	4.14041700	-0.28799800	1.09732300
H	2.08808500	0.09765300	1.55953000
C	4.58111300	-1.32369100	-1.03040500
H	2.85538400	-1.75379000	-2.23434400
C	5.04695700	-0.79399100	0.16799000
H	4.49682300	0.12458900	2.03370000
H	5.27732400	-1.72124000	-1.75912500
H	6.10961300	-0.77444300	0.37862700
C	0.69888200	1.68347700	-0.28608100
C	1.67145200	2.08252700	-1.20669800
C	0.43705000	2.48766900	0.81913600
C	2.39063700	3.25308500	-1.00963200
H	1.87407300	1.46645100	-2.07716300
C	1.16400600	3.66127600	1.01957100
H	-0.33863500	2.21130100	1.52199700
C	2.14450600	4.04350800	0.11256700
H	3.14456600	3.54904100	-1.72927400
H	0.95598200	4.27715400	1.88660300
H	2.70861100	4.95481700	0.27089100
C	-3.35792400	-0.21628700	-0.05098900
C	-3.57125600	1.16550600	-0.01763800
C	-4.45553500	-1.06709900	-0.23647700
C	-4.85456900	1.68498300	-0.17177400
H	-2.73115000	1.83327000	0.12772200
C	-5.73232100	-0.54630300	-0.38627200
H	-4.28732100	-2.13691400	-0.26091600
C	-5.93812200	0.83364300	-0.35472500
H	-5.00469500	2.75796100	-0.14664800

H	-6.57250600	-1.21618200	-0.52690600
H	-6.93649400	1.23819200	-0.47143100
Li	-1.16772488	-2.07302296	2.87477630
H	0.73018800	-1.23679300	-1.74117000

```
Zero-point correction=                           0.338057 (Hartree/Particle)
 Thermal correction to Energy=                         0.360279
 Thermal correction to Enthalpy=                       0.361223
 Thermal correction to Gibbs Free Energy=              0.284479
 Sum of electronic and zero-point Energies=           -1479.462155
 Sum of electronic and thermal Energies=              -1479.439934
 Sum of electronic and thermal Enthalpies=            -1479.438990
 Sum of electronic and thermal Free Energies=         -1479.515733
```

10_{trans}′

S	-0.03881500	-2.19964800	-0.10576200
O	0.51107800	-2.68842500	-1.38048300
O	0.08193500	-3.16486300	1.03310300
C	0.85201700	-0.68390600	0.35905800
H	0.54871000	-0.49733500	1.39275200
C	0.23692500	0.40813800	-0.54045300
H	0.33559600	0.10579300	-1.59059400
C	-1.69575400	-1.80382800	-0.16866000
C	-1.99560000	-0.49131000	-0.18551000
O	-1.14302900	0.58939900	-0.24662600
C	2.34312200	-0.82815700	0.26183000
C	3.10855600	-0.77851200	1.42742200
C	2.98553700	-0.98302000	-0.97043200
C	4.49539200	-0.87850600	1.36763600
H	2.61593200	-0.65418800	2.38580800
C	4.37075800	-1.08207100	-1.02855200
H	2.40257000	-1.04139300	-1.88151100
C	5.12935100	-1.02917200	0.13867900
H	5.07788700	-0.83550500	2.28015000
H	4.85950800	-1.20157100	-1.98818600
H	6.20903300	-1.10526600	0.08909000
C	0.91705500	1.73672700	-0.31632700
C	1.81273900	2.24230100	-1.25470300
C	0.68184300	2.44430300	0.86315600
C	2.47657600	3.44282900	-1.01459300
H	1.99365100	1.69725500	-2.17491900
C	1.34080300	3.64519000	1.10090500
H	-0.02523700	2.05696000	1.58871200
C	2.24244200	4.14489600	0.16334600
H	3.17176900	3.83008900	-1.74986300
H	1.15138900	4.19187700	2.01708000

H	2.75647300	5.08038600	0.34950300
C	-3.40042200	0.00004300	-0.08952900
C	-3.68066400	1.35229700	0.13072300
C	-4.46687700	-0.89799000	-0.21151400
C	-4.99773900	1.79180900	0.23853600
H	-2.86618100	2.05888500	0.22120700
C	-5.77903200	-0.45668700	-0.10984700
H	-4.25047300	-1.94319100	-0.39433000
C	-6.05080500	0.89170000	0.11900300
H	-5.19844900	2.84209300	0.41486100
H	-6.59324700	-1.16448400	-0.21290300
H	-7.07522100	1.23568200	0.19917500
Li	-1.88330000	-3.49101900	1.27238700

```
Zero-point correction=                    0.337869 (Hartree/Particle)
 Thermal correction to Energy=                   0.360210
 Thermal correction to Enthalpy=                 0.361154
 Thermal correction to Gibbs Free Energy=        0.284093
 Sum of electronic and zero-point Energies=          -1479.461896
 Sum of electronic and thermal Energies=             -1479.439556
 Sum of electronic and thermal Enthalpies=           -1479.438612
 Sum of electronic and thermal Free Energies=        -1479.515673
```

Neutral Oxathiins:

Trans-diaryl oxathiin (5a or 5_{trans})

S	0.03414300	-2.26826000	-0.15255700
O	0.20006300	-3.27695200	0.89439800
O	0.50414600	-2.61917500	-1.49426100
C	-1.63705200	-1.76563600	-0.20550600
H	-2.34684900	-2.57755900	-0.27419400
C	-2.01751700	-0.47908600	-0.10675500
C	0.82550100	-0.71950500	0.37015100
C	0.18707900	0.41181700	-0.45413200
H	0.21658100	0.15785400	-1.51995400
O	-1.18778700	0.57997200	-0.07669100
C	2.32012700	-0.83942500	0.24883900
C	3.08932100	-0.90514200	1.41009800
C	2.95060400	-0.86981300	-0.99793600
C	4.47554700	-0.99547600	1.33046700
H	2.60194600	-0.88064400	2.37866500
C	4.33520500	-0.95979200	-1.07477600
H	2.36210000	-0.83838700	-1.90703000
C	5.09991000	-1.02171700	0.08786700
H	5.06450300	-1.04371300	2.23829500

H	4.81775800	-0.98288700	-2.04437500
H	6.17924100	-1.09073300	0.02389000
C	0.87490200	1.72986900	-0.20867100
C	0.76253500	2.34998200	1.03613200
C	1.65408600	2.31206600	-1.20421200
C	1.42834600	3.54516600	1.28045400
H	0.14611700	1.90314000	1.80866200
C	2.32541900	3.50667500	-0.95673200
H	1.73860200	1.83294900	-2.17368400
C	2.21385300	4.12317700	0.28507000
H	1.33529900	4.02597400	2.24679600
H	2.93072300	3.95515600	-1.73517700
H	2.73399400	5.05393900	0.47740900
C	-3.43931300	-0.07621100	-0.01886100
C	-3.83501500	1.16705900	-0.51941800
C	-4.38888500	-0.92588000	0.55483100
C	-5.17189700	1.54342100	-0.46698300
H	-3.09649600	1.82761700	-0.95548400
C	-5.72295200	-0.54299300	0.60828000
H	-4.08328700	-1.87369600	0.98170100
C	-6.11744500	0.68987400	0.09439200
H	-5.47498600	2.50428000	-0.86467900
H	-6.45278600	-1.20299500	1.06086100
H	-7.15842600	0.98683900	0.13811000
H	0.53415300	-0.60631100	1.41792600

Zero-point correction= 0.348663
(Hartree/Particle)
Thermal correction to Energy= 0.369766
Thermal correction to Enthalpy= 0.370710
Thermal correction to Gibbs Free Energy= 0.296036
Sum of electronic and zero-point Energies= -1472.467229
Sum of electronic and thermal Energies= -1472.446126
Sum of electronic and thermal Enthalpies= -1472.445182
Sum of electronic and thermal Free Energies= -1472.519855

Cis-diaryl oxathiin (5_{cis})

S	-0.34510100	-1.48036400	1.17452800
O	-0.59938300	-2.90817300	1.37303500
O	-0.86428700	-0.57134400	2.19743900
C	1.36312700	-1.22859000	0.94849100
C	1.87102800	-0.54254300	-0.09191900
C	-0.94869900	-1.01820800	-0.46758500
C	-0.25080200	0.25782200	-0.98697100

H	-0.54844700	0.32294200	-2.03477900
O	1.17347800	0.07714200	-1.05851700
C	-2.45596600	-0.98644500	-0.51898700
C	-3.10141600	-1.80836800	-1.44495400
C	-3.21808600	-0.15767300	0.31096900
C	-4.48795200	-1.79741400	-1.55343100
H	-2.51552900	-2.45818100	-2.08588900
C	-4.60464500	-0.15540800	0.20442900
H	-2.73075900	0.47554900	1.04085700
C	-5.24190500	-0.96985100	-0.72759600
H	-4.97578200	-2.43767000	-2.27831900
H	-5.18831200	0.48676400	0.85301800
H	-6.32243700	-0.96160700	-0.80724500
C	-0.60135900	1.57612000	-0.33034000
C	-1.62791400	2.33600500	-0.89181800
C	0.07286000	2.06101600	0.79027700
C	-1.99885200	3.55118200	-0.32615500
H	-2.14462800	1.97080800	-1.77357500
C	-0.29070800	3.28145100	1.34962400
H	0.87865600	1.49256300	1.23881300
C	-1.33075700	4.02504500	0.79864000
H	-2.80076500	4.12921200	-0.76933400
H	0.23903700	3.64990900	2.21978400
H	-1.61271800	4.97385800	1.23929200
C	3.33073200	-0.37901000	-0.28329400
C	3.81635100	0.79213300	-0.87088200
C	4.22376500	-1.37149300	0.12770400
C	5.18548000	0.97487300	-1.02521200
H	3.12023600	1.55625100	-1.19364000
C	5.59085700	-1.18610200	-0.03495200
H	3.85095700	-2.29689800	0.55049200
C	6.07400600	-0.01202300	-0.60751900
H	5.55825300	1.88842600	-1.47214400
H	6.27864300	-1.96249800	0.27689000
H	7.14066000	0.12996900	-0.73331600
H	1.98937600	-1.66369200	1.71433400
H	-0.58815600	-1.84600200	-1.08446600

Zero-point correction= (Hartree/Particle)	0.349052
Thermal correction to Energy=	0.369982
Thermal correction to Enthalpy=	0.370926
Thermal correction to Gibbs Free Energy=	0.297307
Sum of electronic and zero-point Energies=	-1472.460919
Sum of electronic and thermal Energies=	-1472.439990
Sum of electronic and thermal Enthalpies=	-1472.439046
Sum of electronic and thermal Free Energies=	-1472.512665

Anionic Oxathiin Prior to Ring Opening:

11a

S	-0.70999500	-0.86877000	1.33398300
O	-1.13159700	-2.25135200	1.76538500
O	-1.03190500	-0.00460500	2.53794600
C	1.02238300	-0.83268500	1.13068400
C	1.62795400	-0.61226600	-0.05339200
C	-1.24382900	-0.47969200	-0.19273200
C	-0.31150500	0.42848800	-0.93196600
H	-0.69634000	0.54410500	-1.94475100
O	0.99820500	-0.15754800	-1.14445200
C	-2.61302700	-0.73989800	-0.61239200
C	-2.95194200	-0.79093100	-1.98043700
C	-3.66196500	-0.94674400	0.30752800
C	-4.25366500	-1.03629500	-2.39732400
H	-2.18152400	-0.66291500	-2.73221000
C	-4.95781500	-1.21512900	-0.11606900
H	-3.46277800	-0.88011800	1.37121800
C	-5.27161900	-1.25784800	-1.47196000
H	-4.47035600	-1.06874800	-3.45944200
H	-5.73400200	-1.37216500	0.62493700
H	-6.28468200	-1.45488300	-1.80027400
C	-0.14400700	1.83547200	-0.34700400
C	-1.26754300	2.46995600	0.18574500
C	1.06707300	2.52478300	-0.38437900
C	-1.18370800	3.77177900	0.66659800
H	-2.20861500	1.93252600	0.23298800
C	1.15621000	3.82286700	0.11437600
H	1.94971600	2.05199400	-0.79747400
C	0.03164100	4.45181200	0.63760500
H	-2.06547400	4.25184400	1.07476900
H	2.10774500	4.34121200	0.09094200
H	0.10091700	5.46246700	1.02222400
C	3.07658300	-0.83239000	-0.27141100
C	3.72273700	-0.18575900	-1.32909600
C	3.80992700	-1.68674100	0.55841500
C	5.08500900	-0.37171700	-1.53692600
H	3.15518000	0.45830800	-1.98861500
C	5.17045800	-1.86779100	0.34958800
H	3.31538500	-2.22967200	1.35457200
C	5.81265600	-1.20766700	-0.69599700
H	5.57704700	0.13726200	-2.35683900
H	5.72840700	-2.53431400	0.99594700
H	6.87391400	-1.35275600	-0.85870700
H	1.56524400	-1.11813200	2.02023900

Li	-1.63984000	-1.58243000	3.58633000

```
Zero-point correction=                           0.337219 (Hartree/Particle)
Thermal correction to Energy=                          0.359400
Thermal correction to Enthalpy=                        0.360345
Thermal correction to Gibbs Free Energy=               0.284057
Sum of electronic and zero-point Energies=          -1479.460587
Sum of electronic and thermal Energies=             -1479.438405
Sum of electronic and thermal Enthalpies=           -1479.437461
Sum of electronic and thermal Free Energies=        -1479.513749
```

11b

S	0.94360500	-0.29168200	1.58313800
O	1.73087900	0.70216100	2.38309800
O	0.81189300	-1.57956100	2.28227200
C	-0.66297700	0.39956900	1.41539700
C	-1.22094500	0.61919700	0.20668500
C	1.49978400	-0.36649600	-0.01959200
C	0.38485600	-0.76959700	-0.92169400
H	0.75721900	-0.73229000	-1.94718600
O	-0.72901700	0.19221400	-0.96484900
C	2.48408700	0.61331600	-0.46980800
C	2.26740400	1.42068000	-1.60667600
C	3.73022100	0.76039600	0.18054000
C	3.23447300	2.31066900	-2.06157300
H	1.31929800	1.36427300	-2.12954300
C	4.68096700	1.67562300	-0.25990100
H	3.96236700	0.11012900	1.01724000
C	4.44481100	2.45887800	-1.38860800
H	3.02872200	2.91032400	-2.94161700
H	5.62694500	1.75281100	0.26540700
H	5.19166100	3.15955900	-1.74118600
C	-0.18141800	-2.17001400	-0.69516500
C	0.71633000	-3.23815700	-0.63373800
C	-1.54592900	-2.43114900	-0.60126400
C	0.25989100	-4.54035600	-0.47919600
H	1.77927500	-3.03304300	-0.69085000
C	-2.00511800	-3.73728400	-0.43032900
H	-2.26399700	-1.62225500	-0.65504300
C	-1.10662000	-4.79512900	-0.37011100
H	0.97040200	-5.35758100	-0.43419100
H	-3.06973500	-3.92231200	-0.34651000
H	-1.46445100	-5.80948400	-0.23888100
C	-2.49981100	1.35629200	0.04527300
C	-3.30065900	1.11360100	-1.07447000
C	-2.91784900	2.29428600	0.99419500

C	-4.51058400	1.78174900	-1.22931400
H	-2.97211000	0.40046900	-1.82009000
C	-4.12640900	2.96019800	0.83626800
H	-2.28878400	2.52148800	1.84647200
C	-4.92802000	2.70315000	-0.27391000
H	-5.12683500	1.58151700	-2.09756100
H	-4.43821200	3.68897400	1.57467300
H	-5.86965900	3.22491800	-0.39624200
H	-1.14808300	0.69343300	2.33579600
Li	2.73978700	2.23365500	2.16229400

Zero-point correction= 0.337510 (Hartree/Particle)
Thermal correction to Energy= 0.359426
Thermal correction to Enthalpy= 0.360371
Thermal correction to Gibbs Free Energy= 0.285833
Sum of electronic and zero-point Energies= -1479.458568
Sum of electronic and thermal Energies= -1479.436652
Sum of electronic and thermal Enthalpies= -1479.435707
Sum of electronic and thermal Free Energies= -1479.510245

11c

S	-0.97000000	-0.32787100	1.59669600
O	-0.84529200	-1.74705200	2.05790100
O	-1.68098600	0.51039800	2.57417100
C	0.66780400	0.29356600	1.45405200
H	1.13926300	0.61029400	2.37435700
C	1.21949000	0.54308400	0.24779000
C	-1.57923700	-0.20366300	0.01775700
C	-0.52863400	-0.60857300	-0.95908800
H	-0.92117100	-0.41696400	-1.95978600
O	0.68334400	0.23045900	-0.93959100
C	-2.41514900	0.96647900	-0.28806100
C	-3.59808100	1.22892400	0.43280700
C	-2.11010900	1.84248300	-1.34820900
C	-4.41582900	2.30501600	0.11894400
H	-3.87437700	0.56444000	1.24187000
C	-2.94970400	2.90308000	-1.68009600
H	-1.19354400	1.70895600	-1.91189000
C	-4.10572100	3.14970000	-0.94750100
H	-5.31679000	2.47430100	0.69862100
H	-2.68273100	3.55152900	-2.50756900
H	-4.75361200	3.98038100	-1.20040700
C	-0.09252000	-2.07078800	-0.92549300
C	1.24484300	-2.46115900	-0.90072300
C	-1.08353000	-3.05445000	-0.98460200
C	1.58562300	-3.81507700	-0.90789200
H	2.02944700	-1.71470000	-0.87699200
C	-0.74499200	-4.40168400	-1.00603800

H	-2.12337600	-2.74818900	-0.99649500
C	0.59505100	-4.78875900	-0.96042300
H	2.63024700	-4.10308400	-0.88067100
H	-1.52563500	-5.15199800	-1.05618500
H	0.86091100	-5.83921600	-0.97335800
C	2.52474600	1.23738500	0.10714800
C	3.50354600	1.14080200	1.09982000
C	2.78053800	1.99970000	-1.03608700
C	4.71183400	1.81318400	0.95961000
H	3.33173400	0.52257900	1.97288400
C	3.98773100	2.67633000	-1.16930500
H	2.02700100	2.06544900	-1.81075400
C	4.95566700	2.58597700	-0.17272100
H	5.46702800	1.72551000	1.73141300
H	4.17279400	3.27429700	-2.05360300
H	5.89821100	3.10922300	-0.28059000
Li	0.05929300	-3.33555500	2.16401500

Zero-point correction= 0.337169 (Hartree/Particle)
Thermal correction to Energy= 0.359249
Thermal correction to Enthalpy= 0.360193
Thermal correction to Gibbs Free Energy= 0.284599
Sum of electronic and zero-point Energies= -1479.458787
Sum of electronic and thermal Energies= -1479.436708
Sum of electronic and thermal Enthalpies= -1479.435764
Sum of electronic and thermal Free Energies= -1479.511358

11d

S	-0.95061400	0.30577500	1.58361800
O	-1.73938000	-0.69367400	2.37733100
O	-0.82372700	1.59140500	2.28726100
C	0.65749700	-0.38308500	1.41526900
C	1.21109700	-0.61126300	0.20586200
C	-1.50727200	0.38042700	-0.01902100
C	-0.39240100	0.78072500	-0.92170500
H	-0.76449400	0.74547500	-1.94734300
O	0.71771000	-0.18711200	-0.96574400
C	-2.48880300	-0.60609200	-0.46445800
C	-2.27648900	-1.40573300	-1.60766100
C	-3.72532200	-0.76870700	0.19990000
C	-3.24086800	-2.30143500	-2.05826600
H	-1.33476500	-1.33827600	-2.14065100
C	-4.67096900	-1.69042400	-0.23495200
H	-3.94897300	-0.13469600	1.05011500
C	-4.44183600	-2.46366500	-1.37249700
H	-3.03975300	-2.89346700	-2.94453700

H	-5.60874200	-1.78195600	0.30252400
H	-5.18682100	-3.16861400	-1.72073800
C	0.18177600	2.17787500	-0.69475900
C	1.54849500	2.43202000	-0.61229600
C	-0.70984200	3.25044700	-0.62272000
C	2.01597200	3.73527200	-0.44262200
H	2.26197000	1.61967600	-0.67438500
C	-0.24518000	4.54999200	-0.46949400
H	-1.77426600	3.05092300	-0.67069900
C	1.12347100	4.79759300	-0.37218900
H	3.08225300	3.91455400	-0.36788300
H	-0.95104400	5.37072800	-0.41626400
H	1.48769500	5.80980800	-0.24203000
C	2.48498100	-1.35705600	0.04448600
C	3.28275700	-1.12768200	-1.08019100
C	2.90032300	-2.29191700	0.99776000
C	4.48680900	-1.80618200	-1.23600800
H	2.95631300	-0.41692900	-1.82899900
C	4.10297800	-2.96817300	0.83892600
H	2.27364600	-2.50866400	1.85451100
C	4.90143200	-2.72463000	-0.27654100
H	5.10070300	-1.61641600	-2.10824900
H	4.41250000	-3.69441600	1.58078800
H	5.83836800	-3.25459000	-0.39971500
H	1.14495400	-0.67312700	2.33571300
Li	-2.38938800	-2.39906900	2.03608200

Zero-point correction= 0.337330 (Hartree/Particle)
Thermal correction to Energy= 0.359310
Thermal correction to Enthalpy= 0.360254
Thermal correction to Gibbs Free Energy= 0.285552
Sum of electronic and zero-point Energies= -1479.458761
Sum of electronic and thermal Energies= -1479.436781
Sum of electronic and thermal Enthalpies= -1479.435837
Sum of electronic and thermal Free Energies= -1479.510539

11e

S	0.22609800	-2.18309500	-0.09581500
O	0.30068200	-3.01105200	1.16253000
O	0.69731500	-2.93676400	-1.27412000
C	-1.46041600	-1.77984500	-0.33306000
H	-2.08355600	-2.62876100	-0.57588500
C	-1.94747900	-0.55000400	-0.09840600
C	0.96748500	-0.66328700	0.27476800
C	0.19389000	0.47761000	-0.35587300
H	0.09472600	0.39889000	-1.45183000

O	-1.17868800	0.52359200	0.13332400
C	2.43376100	-0.73425500	0.00616500
C	3.24225900	-1.67453400	0.67344900
C	3.07332600	0.10047600	-0.92615900
C	4.60700500	-1.76618100	0.43204300
H	2.79387300	-2.35362400	1.39051000
C	4.44531500	0.02456100	-1.15071400
H	2.50053800	0.82181100	-1.49493400
C	5.22447400	-0.90753900	-0.47454400
H	5.19272100	-2.50623600	0.96560600
H	4.90185600	0.69295400	-1.87218600
H	6.29126500	-0.96854800	-0.65288800
C	0.73913400	1.85031600	-0.01779800
C	0.63073300	2.87449800	-0.95651400
C	1.29181400	2.12510200	1.23200800
C	1.08101500	4.15767000	-0.65850000
H	0.19913100	2.66351700	-1.92993300
C	1.74093000	3.40655300	1.53328100
H	1.37965600	1.32712200	1.96026200
C	1.63867200	4.42517500	0.58810400
H	0.99808300	4.94488800	-1.39846500
H	2.17498300	3.61109300	2.50500500
H	1.99350600	5.42188300	0.82251100
C	-3.39567600	-0.24118500	-0.07513400
C	-3.82970400	1.04632000	-0.40235200
C	-4.33380200	-1.21776300	0.27106800
C	-5.18733500	1.34506000	-0.40427900
H	-3.09988500	1.80325400	-0.66102500
C	-5.68951900	-0.91457200	0.26952600
H	-4.00382800	-2.20791600	0.56255500
C	-6.11913400	0.36606800	-0.07064000
H	-5.51771700	2.34285700	-0.66661400
H	-6.41046900	-1.67482800	0.54446800
H	-7.17673200	0.60136800	-0.06880700
Li	0.50643500	-1.48929200	2.41406100

```
Zero-point correction=                           0.337177 (Hartree/Particle)
Thermal correction to Energy=                          0.359435
Thermal correction to Enthalpy=                        0.360379
Thermal correction to Gibbs Free Energy=               0.284406
Sum of electronic and zero-point Energies=                -1479.454916
Sum of electronic and thermal Energies=                   -1479.432658
Sum of electronic and thermal Enthalpies=                 -1479.431714
Sum of electronic and thermal Free Energies=              -1479.507687
```

11f

S	-0.09011000	-1.97541500	-1.50631000
O	-0.00697400	-3.33958900	-0.89187700
O	-0.03296500	-1.99797800	-2.97998100
C	-1.64226800	-1.30397200	-1.04284600
H	-2.52163900	-1.92352600	-1.14807000
C	-1.69428900	-0.05511900	-0.52654500
C	1.06275100	-0.92976900	-0.82443800
C	0.58932000	0.45342000	-1.14895000
H	0.33695600	0.52693700	-2.21264400
O	-0.67274700	0.80487900	-0.44621600
C	1.47968200	-1.17833300	0.58706100
C	0.64291900	-1.69236200	1.59165100
C	2.78805200	-0.82751300	0.96185300
C	1.09729200	-1.86282000	2.89742700
H	-0.37956600	-1.96203800	1.35178700
C	3.23659700	-0.97174200	2.26999500
H	3.45364100	-0.42981500	0.20291900
C	2.39426200	-1.49858100	3.24684800
H	0.42663600	-2.26666500	3.64796800
H	4.25071600	-0.68415500	2.52437800
H	2.74388500	-1.62268900	4.26510500
C	1.57412500	1.53977600	-0.79372900
C	1.50700900	2.23005800	0.41395400
C	2.61881500	1.80915900	-1.67808700
C	2.48057500	3.17294600	0.73715000
H	0.69778600	2.02256900	1.10319600
C	3.59107300	2.74903800	-1.35617000
H	2.67392100	1.26711100	-2.61685200
C	3.52576800	3.43303600	-0.14331000
H	2.42206600	3.70225400	1.68121300
H	4.39820200	2.94976200	-2.05101100
H	4.28271200	4.16603200	0.10977100
C	-2.95278800	0.51610700	0.01650600
C	-3.16539500	1.89616200	-0.03856000
C	-3.92827600	-0.30634500	0.58652700
C	-4.34731700	2.44167700	0.45002100
H	-2.40448100	2.53287900	-0.47199000
C	-5.10727000	0.24234100	1.07642000
H	-3.75595300	-1.37312200	0.66775200
C	-5.32108600	1.61687700	1.00623900
H	-4.50796800	3.51173200	0.39572100
H	-5.85472700	-0.40213900	1.52317400
H	-6.24025600	2.04336900	1.38990400
Li	1.51467400	-4.34486700	-0.48362300

Zero-point correction= 0.336360 (Hartree/Particle)
Thermal correction to Energy= 0.358892
Thermal correction to Enthalpy= 0.359836

```
Thermal correction to Gibbs Free Energy=         0.282612
Sum of electronic and zero-point Energies=           -1479.457720
Sum of electronic and thermal Energies=              -1479.435188
Sum of electronic and thermal Enthalpies=            -1479.434244
Sum of electronic and thermal Free Energies=         -1479.511468
```

11g

S	0.14304300	-1.83624700	-1.59410300
O	0.35313200	-3.20979900	-1.03776900
O	0.20239800	-1.80245000	-3.06472900
C	-1.47944300	-1.35457100	-1.10705700
H	-2.27679700	-2.05939700	-1.29940800
C	-1.69668700	-0.18741300	-0.46144500
C	1.13608400	-0.69286100	-0.82202100
C	0.48396600	0.62881500	-1.02105700
H	0.17798000	0.72427900	-2.07168100
O	-0.79004200	0.77906700	-0.27678400
C	1.73808300	-1.06311200	0.46202600
C	1.52055300	-0.31721400	1.63834100
C	2.61501600	-2.16461900	0.56090000
C	2.14011300	-0.65684800	2.83705700
H	0.85213300	0.53508400	1.61285400
C	3.20558200	-2.52032900	1.76873900
H	2.85061400	-2.72550100	-0.33633800
C	2.97700300	-1.76672700	2.91913100
H	1.95249300	-0.05464100	3.71937800
H	3.87445500	-3.37359800	1.80338700
H	3.45194900	-2.03222800	3.85590100
C	1.33355800	1.83583500	-0.67277000
C	0.76953700	2.99718500	-0.14426900
C	2.70363100	1.80802700	-0.93835100
C	1.56518500	4.10969200	0.11994800
H	-0.29076200	3.02996300	0.06994700
C	3.49554000	2.92227700	-0.68159400
H	3.14282300	0.90261900	-1.34040500
C	2.92973500	4.07763800	-0.14842600
H	1.11428200	5.00309400	0.53623900
H	4.55799600	2.88496200	-0.89245900
H	3.54754900	4.94383800	0.05702700
C	-3.02189100	0.16282800	0.10846600
C	-3.41211300	1.50302600	0.17443300
C	-3.88627300	-0.82728500	0.58282400
C	-4.65709000	1.84486400	0.69033100
H	-2.74059000	2.27013000	-0.19014200
C	-5.12910200	-0.48242000	1.10013700
H	-3.57771600	-1.86591600	0.56771700

C	-5.51849300	0.85379700	1.15259500
H	-4.95496400	2.88572400	0.73030700
H	-5.78969000	-1.25616500	1.47239100
H	-6.48698100	1.12134300	1.55801200
Li	0.43588500	-3.98389500	0.65985200

```
Zero-point correction=                           0.337058 (Hartree/Particle)
 Thermal correction to Energy=                    0.359219
 Thermal correction to Enthalpy=                  0.360164
 Thermal correction to Gibbs Free Energy=         0.284623
 Sum of electronic and zero-point Energies=          -1479.460880
 Sum of electronic and thermal Energies=             -1479.438719
 Sum of electronic and thermal Enthalpies=           -1479.437775
 Sum of electronic and thermal Free Energies=        -1479.513315
```

11h

S	-0.12352600	-2.09052600	-1.24266600
O	-0.08169800	-3.42390700	-0.56758200
O	-0.03004600	-2.33420400	-2.72105300
C	-1.66251000	-1.32860200	-0.92281600
H	-2.55486400	-1.93229600	-1.00787800
C	-1.69341000	-0.04578400	-0.48609300
C	1.04204500	-1.00901800	-0.67501000
C	0.59399000	0.34492800	-1.12839700
H	0.32916000	0.32168300	-2.19221300
O	-0.65329200	0.78846600	-0.45425700
C	1.49152200	-1.14863500	0.73987300
C	0.66706800	-1.56706100	1.79543400
C	2.81093200	-0.78717000	1.05623200
C	1.14452000	-1.63780800	3.10171300
H	-0.36205000	-1.84470300	1.59494600
C	3.28292900	-0.83119800	2.36322100
H	3.46645900	-0.46385600	0.25458500
C	2.45271200	-1.26447400	3.39480000
H	0.48521400	-1.97287700	3.89472000
H	4.30580600	-0.54011800	2.57461100
H	2.82143900	-1.31150200	4.41277100
C	1.60827800	1.43564400	-0.89307400
C	1.58656500	2.22921600	0.25102500
C	2.63481700	1.60076800	-1.82270500
C	2.58922100	3.17243200	0.46652700
H	0.79163800	2.10097700	0.97555000
C	3.63612300	2.54044600	-1.60750200

H	2.65349500	0.97800900	-2.71142200
C	3.61698400	3.32852600	-0.45804400
H	2.56759400	3.78293600	1.36179900
H	4.42962900	2.65983000	-2.33586500
H	4.39673000	4.06146700	-0.28773400
C	-2.94398900	0.58653600	0.00117700
C	-3.11867200	1.96630900	-0.13517600
C	-3.94648100	-0.17801300	0.60408100
C	-4.29070000	2.56853200	0.30790200
H	-2.33636100	2.55753900	-0.59422400
C	-5.11543100	0.42781600	1.04798500
H	-3.80251400	-1.24223600	0.74874700
C	-5.29130400	1.80136600	0.89805400
H	-4.42257300	3.63761500	0.19256100
H	-5.88427200	-0.17037600	1.52184500
H	-6.20250400	2.27258000	1.24679000
Li	0.28114300	-4.30587500	-2.40634600

Zero-point correction= 0.336562 (Hartree/Particle)
Thermal correction to Energy= 0.358922
Thermal correction to Enthalpy= 0.359866
Thermal correction to Gibbs Free Energy= 0.283174
Sum of electronic and zero-point Energies= -1479.457554
Sum of electronic and thermal Energies= -1479.435194
Sum of electronic and thermal Enthalpies= -1479.434250
Sum of electronic and thermal Free Energies= -1479.510942

Transition States for Ring Opening:

11a‡

S	-0.98072700	0.10765800	1.52246600
O	-1.79300600	-0.97987200	2.16231000
O	-1.02609900	1.26323000	2.48186800
C	0.63723400	-0.40555200	1.27999900
C	1.19591100	-0.69885200	0.04820500
C	-1.58044600	0.40559500	-0.07356000
C	-0.63043100	0.90906800	-0.96052400
H	-0.91873700	0.81020200	-2.00432600
O	0.75406900	-0.36850100	-1.10922800
C	-2.68456400	-0.46285900	-0.54093700
C	-2.52252500	-1.29213800	-1.65888700
C	-3.93171000	-0.45204200	0.09825900
C	-3.57774900	-2.06647400	-2.13320600
H	-1.55560900	-1.34107800	-2.14780100
C	-4.97866500	-1.24078500	-0.36272900

H	-4.07889900	0.19240000	0.95778700
C	-4.80948600	-2.04822400	-1.48609300
H	-3.42989000	-2.69749000	-3.00218000
H	-5.93510400	-1.21421200	0.14691700
H	-5.62902800	-2.65546500	-1.85158000
C	0.22539000	2.11475200	-0.71618800
C	-0.17515800	3.10601400	0.18045700
C	1.37530400	2.32646900	-1.48771600
C	0.58353100	4.26434600	0.33811300
H	-1.08249400	2.97118700	0.75316200
C	2.13096300	3.47965400	-1.32886800
H	1.67415700	1.57389700	-2.20647600
C	1.74082300	4.45149200	-0.40758100
H	0.26232600	5.02186500	1.04337700
H	3.02226300	3.62531100	-1.92781800
H	2.33007200	5.35257500	-0.28430700
C	2.50297600	-1.43102100	0.04105800
C	3.42318100	-1.14719300	-0.97092000
C	2.81696500	-2.39332900	1.00329100
C	4.65038300	-1.79892900	-1.00650000
H	3.16685300	-0.40975500	-1.72162400
C	4.04068000	-3.05310500	0.95988400
H	2.09476300	-2.64462300	1.77123300
C	4.96137500	-2.75336600	-0.04088700
H	5.36349200	-1.56468500	-1.78794400
H	4.27224800	-3.80698400	1.70284700
H	5.91504500	-3.26692900	-0.07212900
H	1.15427700	-0.63577400	2.20063800
Li	-2.25924100	0.25568300	3.71438600

Zero-point correction= 0.334559 (Hartree/Particle)
Thermal correction to Energy= 0.356912
Thermal correction to Enthalpy= 0.357856
Thermal correction to Gibbs Free Energy= 0.281198
Sum of electronic and zero-point Energies= -1479.449625
Sum of electronic and thermal Energies= -1479.427272
Sum of electronic and thermal Enthalpies= -1479.426328
Sum of electronic and thermal Free Energies= -1479.502986

Imaginary Frequency: -359.61 cm^{-1}

11b‡

S	0.73294300	0.79480400	1.57324900
O	0.30053300	2.01548600	2.32357600
O	1.87149500	0.11173000	2.19939700
C	-0.66586800	-0.22323600	1.46637100
C	-0.94040000	-0.95329600	0.33595800

C	1.01511200	1.18469300	-0.09288200
C	1.24864800	0.03095600	-0.86201100
H	1.04576400	0.18947600	-1.91982200
O	-0.15275300	-1.14340400	-0.67276700
C	0.13472500	2.25291300	-0.62914800
C	-0.92981500	1.96565900	-1.49618600
C	0.36674600	3.59616400	-0.29540600
C	-1.72513400	2.98394400	-2.01605700
H	-1.13914400	0.93339900	-1.75754400
C	-0.44278900	4.61113300	-0.79520000
H	1.20178000	3.83516300	0.35403300
C	-1.49133800	4.30965800	-1.66307200
H	-2.53879000	2.73741800	-2.68879000
H	-0.24076000	5.64175700	-0.52573500
H	-2.11405900	5.10075900	-2.06369500
C	2.40565400	-0.90455000	-0.65559100
C	3.60438100	-0.42145000	-0.12799300
C	2.35483500	-2.22395800	-1.11552500
C	4.72154300	-1.24594000	-0.03676700
H	3.65158300	0.60329600	0.21606300
C	3.47039900	-3.04883800	-1.02053200
H	1.43284000	-2.60366900	-1.53583900
C	4.65813800	-2.56369600	-0.47869100
H	5.64231800	-0.85619600	0.38149800
H	3.41253900	-4.07181500	-1.37391900
H	5.52791400	-3.20626300	-0.40708100
C	-2.27527400	-1.62510100	0.21669000
C	-2.36543200	-2.81374200	-0.51194200
C	-3.42910800	-1.09392600	0.79844500
C	-3.58425700	-3.47061900	-0.64034200
H	-1.47022500	-3.21712400	-0.96951700
C	-4.64947700	-1.74746600	0.66323300
H	-3.38023700	-0.15527000	1.33774100
C	-4.72930600	-2.93952000	-0.05215700
H	-3.64090100	-4.39725400	-1.19922500
H	-5.54018200	-1.32092000	1.10919200
H	-5.68024600	-3.44864700	-0.15538700
H	-1.37217000	-0.12554000	2.27738500
Li	-1.06373300	3.27179100	2.33328200

Zero-point correction= 0.335017 (Hartree/Particle)
Thermal correction to Energy= 0.357131
Thermal correction to Enthalpy= 0.358076
Thermal correction to Gibbs Free Energy= 0.282495
Sum of electronic and zero-point Energies= -1479.449317
Sum of electronic and thermal Energies= -1479.427203
Sum of electronic and thermal Enthalpies= -1479.426258
Sum of electronic and thermal Free Energies= -1479.501839

Imaginary Frequency: -317.88 cm^{-1}

11c‡

S	-0.73758600	0.78874900	1.60050000
O	-1.92811300	-0.02100100	1.99335900
O	-0.45171000	1.91924400	2.49564100
C	0.67232800	-0.22258400	1.54512400
H	1.45464700	0.03952200	2.24225500
C	0.90441600	-0.98423300	0.42695200
C	-0.89070800	1.24571100	-0.05945600
C	-1.14585600	0.11605000	-0.87092500
H	-0.83088600	0.27554800	-1.90091900
O	0.05483100	-1.18117400	-0.53544300
C	0.13593500	2.22916000	-0.49067600
C	0.08661900	3.55453300	-0.03444400
C	1.17122300	1.87709700	-1.36990300
C	1.02969300	4.48988700	-0.44341600
H	-0.70573200	3.84314100	0.64570300
C	2.10402700	2.81878700	-1.79567600
H	1.25802400	0.85182900	-1.71485000
C	2.04048800	4.12946800	-1.33270900
H	0.96809600	5.50826800	-0.07667200
H	2.89151400	2.52115200	-2.47890100
H	2.76985000	4.86155800	-1.65863700
C	-2.42491100	-0.67335500	-0.82292100
C	-2.47778800	-1.99284000	-1.27933200
C	-3.61304100	-0.04285300	-0.44903400
C	-3.68966000	-2.67519000	-1.33032500
H	-1.56424500	-2.48660900	-1.58326000
C	-4.82531300	-0.72328100	-0.50412800
H	-3.57453100	0.98412200	-0.10869000
C	-4.86838600	-2.04428800	-0.94146400
H	-3.71283100	-3.70062600	-1.68089600
H	-5.73766600	-0.21894800	-0.20687300
H	-5.81241100	-2.57474400	-0.98693100
C	2.24227400	-1.62401000	0.23549100
C	3.04383800	-1.98673100	1.32086100
C	2.69992800	-1.86975400	-1.06158600
C	4.29160400	-2.56364100	1.11109200
H	2.68456300	-1.83768900	2.33254000
C	3.95185200	-2.43745100	-1.26987800
H	2.06682100	-1.60841000	-1.90080400
C	4.75195400	-2.78536400	-0.18438200
H	4.90174100	-2.84757200	1.96036600

H	4.30229100	-2.61194500	-2.28036100
H	5.72459100	-3.23447700	-0.34657600
Li	-2.60854700	-1.04378900	3.37049300

Zero-point correction= 0.334759 (Hartree/Particle)
Thermal correction to Energy= 0.357050
Thermal correction to Enthalpy= 0.357994
Thermal correction to Gibbs Free Energy= 0.281872
Sum of electronic and zero-point Energies= -1479.450893
Sum of electronic and thermal Energies= -1479.428602
Sum of electronic and thermal Enthalpies= -1479.427658
Sum of electronic and thermal Free Energies= -1479.503781

Imaginary Frequency -320.29 cm^{-1}

11d‡

S	-0.97566600	0.19936500	1.59022200
O	-1.84068600	-0.89013800	2.15450000
O	-0.88838200	1.33632700	2.52133000
C	0.59749500	-0.45963100	1.30235200
C	1.13409400	-0.72787100	0.06221100
C	-1.57288100	0.56042100	-0.00864400
C	-0.59686000	1.02016300	-0.89116900
H	-0.89410500	0.96207300	-1.93561900
O	0.69489100	-0.34381700	-1.08650700
C	-2.69469200	-0.27094000	-0.49363000
C	-2.55641500	-1.09173900	-1.62345700
C	-3.94077900	-0.24848500	0.15370500
C	-3.62868100	-1.84152400	-2.10020400
H	-1.59369800	-1.15078600	-2.11927400
C	-5.00413100	-1.01521400	-0.31021900
H	-4.07141500	0.38983500	1.02038700
C	-4.85601500	-1.81214100	-1.44464700
H	-3.49772100	-2.46258700	-2.97912200
H	-5.95741500	-0.97560200	0.20458600
H	-5.68850300	-2.39948800	-1.81329500
C	0.32853100	2.16991800	-0.62745500
C	1.50029500	2.31929600	-1.38037700
C	-0.02870400	3.17992500	0.26706200
C	2.31673500	3.42845900	-1.20670600
H	1.76666700	1.55340000	-2.09755600
C	0.79051800	4.29379700	0.43940100
H	-0.94900200	3.09222500	0.82802600

C	1.96745000	4.41850900	-0.28879300
H	3.22398600	3.52505500	-1.79158100
H	0.50177500	5.06552200	1.14328500
H	2.60409300	5.28501600	-0.15359300
C	2.41362700	-1.50911600	0.01701600
C	3.32816200	-1.23915500	-1.00385800
C	2.71022500	-2.50224500	0.95358300
C	4.53091900	-1.93306300	-1.07304900
H	3.08676200	-0.47832400	-1.73570800
C	3.90935800	-3.20313200	0.87802000
H	1.99362900	-2.74277200	1.73023000
C	4.82443800	-2.91673100	-0.13183000
H	5.23894700	-1.70810400	-1.86191100
H	4.12603000	-3.97841500	1.60339000
H	5.75881200	-3.46255800	-0.18872700
H	1.11552300	-0.73040600	2.21158000
Li	-2.57214800	-2.50330400	1.61363000

Zero-point correction= 0.334986 (Hartree/Particle)
Thermal correction to Energy= 0.357162
Thermal correction to Enthalpy= 0.358106
Thermal correction to Gibbs Free Energy= 0.282244
Sum of electronic and zero-point Energies= -1479.449379
Sum of electronic and thermal Energies= -1479.427203
Sum of electronic and thermal Enthalpies= -1479.426259
Sum of electronic and thermal Free Energies= -1479.502121

Imaginary Frequency -353.59 cm^{-1}

11e‡

S	0.17398200	-2.19128900	-0.18443500
O	0.15857300	-2.45164800	1.30615800
O	0.59312200	-3.37182500	-0.95445500
C	-1.49967500	-1.78035000	-0.56158900
H	-2.05823400	-2.50163300	-1.13849900
C	-1.96894400	-0.66052500	0.02603400
C	1.05931900	-0.74492100	-0.46270300
C	0.19122600	0.40494800	-0.54169500
H	-0.49879200	0.44372300	-1.38638100
O	-1.09905100	0.19500600	0.59629900
C	2.51028200	-0.77190100	-0.26074100
C	3.16201400	-1.74683700	0.51989300
C	3.33039900	0.17230500	-0.91348200
C	4.54781800	-1.76400900	0.64736600
H	2.58064300	-2.49692400	1.04192200
C	4.70882000	0.17086100	-0.75562200

H	2.87269500	0.91089000	-1.56135500
C	5.33460700	-0.80027900	0.02554200
H	5.01188200	-2.53417500	1.25365600
H	5.30184900	0.92006500	-1.26855400
H	6.41217300	-0.81044600	0.13590900
C	0.72334700	1.75873700	-0.21817800
C	0.40254600	2.82981200	-1.05145500
C	1.49401400	1.98801200	0.92523700
C	0.86845400	4.11079400	-0.76646900
H	-0.20822100	2.65772100	-1.93139200
C	1.94358500	3.26765700	1.22032700
H	1.75951200	1.15973800	1.57316500
C	1.63795900	4.33098700	0.37062000
H	0.62451200	4.93385200	-1.42734000
H	2.54005100	3.43813300	2.10865600
H	1.99692900	5.32756000	0.59901800
C	-3.39121500	-0.26063400	0.03986600
C	-3.71863900	1.09285200	0.15779200
C	-4.41066900	-1.21141800	-0.07295200
C	-5.05058600	1.49160000	0.14214600
H	-2.92270300	1.82155500	0.25255200
C	-5.73971800	-0.80934700	-0.08574800
H	-4.16310600	-2.26502100	-0.13122800
C	-6.06190900	0.54272000	0.01923600
H	-5.29952200	2.54265200	0.22586700
H	-6.52568500	-1.55024400	-0.16810200
H	-7.09974400	0.85382700	0.01141900
Li	-0.25577500	-0.80976700	2.25236100

Zero-point correction= 0.335715 (Hartree/Particle)
Thermal correction to Energy= 0.357596
Thermal correction to Enthalpy= 0.358540
Thermal correction to Gibbs Free Energy= 0.284142
Sum of electronic and zero-point Energies= -1479.438068
Sum of electronic and thermal Energies= -1479.416187
Sum of electronic and thermal Enthalpies= -1479.415243
Sum of electronic and thermal Free Energies= -1479.489642

Imaginary Frequency -469.03 cm^{-1}

11f‡

S	-0.11391600	-1.88634400	-1.59081200
O	0.00732400	-3.27644700	-1.04740100
O	-0.02414000	-1.82578600	-3.06139600
C	-1.62560000	-1.25260000	-1.03629700
H	-2.44803300	-1.95069500	-0.99199900

C	-1.72771700	0.03972500	-0.58612700
C	1.05830100	-0.85497300	-0.85748700
C	0.78390900	0.46925500	-1.26104700
H	0.51066900	0.58647800	-2.30754600
O	-0.82406400	0.97002500	-0.65446000
C	1.57458600	-1.19757700	0.49259500
C	0.77343100	-1.71959200	1.51902200
C	2.92741400	-0.95948600	0.77667300
C	1.30646800	-1.99406300	2.77568600
H	-0.27929300	-1.90285700	1.33316800
C	3.45607800	-1.21130500	2.03787300
H	3.56085100	-0.55987400	-0.00815500
C	2.64784600	-1.73529600	3.04432600
H	0.66577000	-2.39617700	3.55251100
H	4.50304600	-1.00770100	2.23216100
H	3.05871000	-1.93965700	4.02604200
C	1.61683200	1.59779500	-0.74776500
C	1.57039900	1.99520000	0.58936800
C	2.48983900	2.24207600	-1.62299200
C	2.39771000	3.01453000	1.04495900
H	0.88089600	1.50240700	1.26450200
C	3.32646900	3.25703300	-1.16477200
H	2.52048000	1.94219700	-2.66528600
C	3.28235900	3.64442400	0.17058600
H	2.35341800	3.31938700	2.08402000
H	4.00511200	3.74754600	-1.85257400
H	3.92788100	4.43788100	0.52840500
C	-3.00929700	0.47648800	0.05344000
C	-3.40065400	1.81252000	-0.06237100
C	-3.81495200	-0.41107300	0.77108300
C	-4.58991200	2.24910800	0.51061500
H	-2.76665200	2.49907900	-0.60954100
C	-5.00068100	0.02847000	1.34999300
H	-3.50460200	-1.44162600	0.89831800
C	-5.39335500	1.35788200	1.21720100
H	-4.88974900	3.28515400	0.40629600
H	-5.61358300	-0.66534800	1.91306400
H	-6.31810400	1.69859200	1.66771100
Li	1.49535300	-4.33561900	-0.66646900

Zero-point correction= 0.334669 (Hartree/Particle)
Thermal correction to Energy= 0.357107
Thermal correction to Enthalpy= 0.358052
Thermal correction to Gibbs Free Energy= 0.280933
Sum of electronic and zero-point Energies= -1479.453266
Sum of electronic and thermal Energies= -1479.430828
Sum of electronic and thermal Enthalpies= -1479.429884
Sum of electronic and thermal Free Energies= -1479.507002

Imaginary Frequency -344.27 cm^{-1}

11g‡

S	0.07014200	-1.75484500	-1.73712400
O	0.23403400	-3.17162700	-1.28264300
O	0.17183800	-1.60198400	-3.19691500
C	-1.46915700	-1.21267900	-1.14710100
H	-2.26521900	-1.94112100	-1.19359000
C	-1.64511700	0.02399400	-0.57531900
C	1.18409500	-0.72504900	-0.90160900
C	0.84942400	0.60508500	-1.20482400
H	0.51419500	0.76779900	-2.22749100
O	-0.79403600	1.00011100	-0.53055300
C	1.59395900	-1.17550300	0.44641600
C	1.15801900	-0.51297500	1.60459800
C	2.44783000	-2.27893100	0.60066600
C	1.56974900	-0.93266900	2.86582000
H	0.49329000	0.33813900	1.50462100
C	2.84117500	-2.71168100	1.86415200
H	2.81625100	-2.78318200	-0.28599900
C	2.40708500	-2.03698400	3.00340300
H	1.22591900	-0.40099800	3.74585400
H	3.50509500	-3.56376800	1.95744600
H	2.72348700	-2.36482700	3.98651200
C	1.64840500	1.75277400	-0.66820700
C	1.09322100	3.03329800	-0.60002800
C	2.98574300	1.57520300	-0.30686000
C	1.85380100	4.10883700	-0.15465900
H	0.05889300	3.17679600	-0.88545400
C	3.74772900	2.65324600	0.13369700
H	3.43211000	0.59079500	-0.37960000
C	3.18303900	3.92209900	0.21701800
H	1.40844900	5.09535800	-0.10072400
H	4.78428900	2.49962200	0.40992900
H	3.77558900	4.76103000	0.56252600
C	-2.96163600	0.33180100	0.07230800
C	-3.40878600	1.65530100	0.08914000
C	-3.74508100	-0.65941700	0.66849000
C	-4.62879400	1.97897200	0.67239300
H	-2.79263900	2.42259400	-0.36332800
C	-4.96177700	-0.33365100	1.25822900
H	-3.39462100	-1.68440500	0.69387100
C	-5.40885400	0.98510900	1.25769600
H	-4.97070000	3.00720300	0.67106800
H	-5.55675000	-1.10874800	1.72656500
H	-6.35740000	1.23739900	1.71677900

Li	0.19279500	-4.02041900	0.38779300

Zero-point correction= 0.335051 (Hartree/Particle)
Thermal correction to Energy= 0.357264
Thermal correction to Enthalpy= 0.358208
Thermal correction to Gibbs Free Energy= 0.282225
Sum of electronic and zero-point Energies= -1479.455450
Sum of electronic and thermal Energies= -1479.433237
Sum of electronic and thermal Enthalpies= -1479.432293
Sum of electronic and thermal Free Energies= -1479.508276

Imaginary Frequency -335.74 cm^{-1}

11h‡

S	0.15326100	-1.98560300	1.35379200
O	0.05401900	-3.35106600	0.75448400
O	0.04357000	-2.14115700	2.84326100
C	1.65094600	-1.26045700	0.91865800
H	2.50654800	-1.91925400	0.91267500
C	1.73075400	0.05004900	0.50167500
C	-1.03956900	-0.91651300	0.73651800
C	-0.76495900	0.38007000	1.21627800
H	-0.43601000	0.43301000	2.25340800
O	0.80311200	0.94834000	0.56480600
C	-1.61754300	-1.19185800	-0.60399600
C	-0.84656100	-1.60713200	-1.69752200
C	-2.98896400	-0.98753100	-0.80395800
C	-1.42830300	-1.81416200	-2.94477700
H	0.21953100	-1.76399900	-1.57091800
C	-3.56821200	-1.17201800	-2.05459800
H	-3.59683700	-0.66950600	0.03626300
C	-2.79005200	-1.59117300	-3.13105900
H	-0.81279400	-2.13814800	-3.77630900
H	-4.63023500	-0.99893400	-2.18617700
H	-3.24061300	-1.74457900	-4.10460300
C	-1.63774200	1.52563000	0.82304500
C	-1.68289800	1.99212500	-0.49183600
C	-2.45031000	2.11734100	1.78855200
C	-2.54095600	3.02955500	-0.83539500
H	-1.04067000	1.53901900	-1.23776200
C	-3.31784600	3.15049700	1.44219600
H	-2.40966500	1.76308400	2.81324000
C	-3.36500200	3.60756300	0.12928900
H	-2.56843600	3.38875100	-1.85747400
H	-3.94945500	3.60041200	2.19900300
H	-4.03506700	4.41495500	-0.14166400
C	3.02212700	0.53461700	-0.07871700

C	3.37262000	1.87730300	0.08158700
C	3.87267000	-0.31551600	-0.78961200
C	4.56732800	2.35774800	-0.44233600
H	2.70324500	2.53400200	0.62325500
C	5.06352900	0.16872000	-1.32002300
H	3.59291300	-1.34987800	-0.95156500
C	5.41552800	1.50433500	-1.14347300
H	4.83623100	3.39833900	-0.30503500
H	5.71195000	-0.49480700	-1.87963300
H	6.34422100	1.87984700	-1.55662600
Li	-0.26404900	-4.12892100	2.65528700

Zero-point correction= 0.334604 (Hartree/Particle)
Thermal correction to Energy= 0.356994
Thermal correction to Enthalpy= 0.357938
Thermal correction to Gibbs Free Energy= 0.280991
Sum of electronic and zero-point Energies= -1479.453380
Sum of electronic and thermal Energies= -1479.430990
Sum of electronic and thermal Enthalpies= -1479.430046
Sum of electronic and thermal Free Energies= -1479.506993

Imaginary Frequency -351.33 cm^{-1}

Ring Opened Products – E and Z Keto Sulfone:

12Za

S	-1.10258000	0.07007400	1.44408600
O	-1.98759900	-1.03602400	1.91021500
O	-1.35845800	1.27317100	2.29923200
C	0.54211600	-0.28618000	1.46156600
C	1.03076300	-1.22318700	0.52488000
C	-1.68018000	0.43824500	-0.23577300
C	-0.90250000	1.01449600	-1.15582000
H	-1.34404500	1.10043200	-2.14811600
O	0.30337800	-1.93932400	-0.18501300
C	-3.06160400	-0.01549800	-0.53270500
C	-3.28421500	-0.97662700	-1.51897700
C	-4.14976900	0.52642400	0.15694200
C	-4.58102400	-1.38461700	-1.81953200
H	-2.43662500	-1.40808600	-2.03941500
C	-5.44307900	0.11588100	-0.14218800
H	-3.97777700	1.28009000	0.91853700
C	-5.66125200	-0.84089300	-1.13191900
H	-4.74497300	-2.13158700	-2.58712200
H	-6.28220900	0.54638700	0.39135400

H	-6.67005600	-1.16043400	-1.36460900
C	0.46822900	1.55176700	-1.02949400
C	0.79652900	2.49871100	-0.05491500
C	1.44769600	1.13317700	-1.93320800
C	2.09178800	2.99473600	0.03045500
H	0.03464300	2.83316200	0.64089100
C	2.74793600	1.61771300	-1.83555600
H	1.19409500	0.40503800	-2.69649100
C	3.07270100	2.54908500	-0.85396300
H	2.33767100	3.72761500	0.78996900
H	3.50653700	1.26552800	-2.52470000
H	4.08435800	2.93054100	-0.78042800
C	2.52905900	-1.30663800	0.35412000
C	3.02125200	-1.89735600	-0.81099500
C	3.43111700	-0.79062800	1.28674700
C	4.38854800	-1.95032000	-1.05443500
H	2.31254300	-2.29890500	-1.52521400
C	4.80105000	-0.84890000	1.04849000
H	3.07171700	-0.35172100	2.21006100
C	5.28268400	-1.42147700	-0.12583200
H	4.75856900	-2.40037700	-1.96841600
H	5.49249200	-0.44851400	1.78066100
H	6.34945000	-1.46116100	-0.31310800
H	1.12975000	0.50215100	1.90222000
Li	-2.72016600	0.19286700	3.41525500

Zero-point correction= 0.334112 (Hartree/Particle)
Thermal correction to Energy= 0.357679
Thermal correction to Enthalpy= 0.358623
Thermal correction to Gibbs Free Energy= 0.278975
Sum of electronic and zero-point Energies= -1479.484502
Sum of electronic and thermal Energies= -1479.460935
Sum of electronic and thermal Enthalpies= -1479.459991
Sum of electronic and thermal Free Energies= -1479.539639

12Zb

S	0.51639300	0.19221000	1.47857000
O	0.49241100	1.34880100	2.43215000
O	1.42710800	-0.87787400	1.88691300
C	-1.10485400	-0.25748300	1.25084400
C	-1.44963000	-1.14521500	0.22249700
C	1.14563700	0.92676800	-0.04849500
C	2.22241800	0.50792300	-0.72206700
H	2.51897200	1.17664200	-1.53087900
O	-0.62765900	-1.72008100	-0.52041900
C	0.43763800	2.18109700	-0.42753300

C	-0.83487500	2.14198800	-1.00520300
C	1.05799000	3.41643200	-0.22236400
C	-1.47451500	3.32320100	-1.36907900
H	-1.31054000	1.18490800	-1.18407400
C	0.41582400	4.59663000	-0.58622100
H	2.04548100	3.44401700	0.22510700
C	-0.85361500	4.55154600	-1.15631200
H	-2.45585400	3.28265600	-1.82698900
H	0.90751900	5.54893600	-0.42605600
H	-1.35385600	5.46946800	-1.44158400
C	3.09898000	-0.67538100	-0.59045700
C	4.48003300	-0.47884600	-0.69488100
C	2.59984400	-1.97510000	-0.46708300
C	5.35550200	-1.55727800	-0.62502300
H	4.86903200	0.52606500	-0.82253100
C	3.47732000	-3.05245400	-0.42090100
H	1.52800800	-2.12310600	-0.41355700
C	4.85413600	-2.84862900	-0.48818900
H	6.42415900	-1.39018000	-0.68952200
H	3.08404500	-4.05846700	-0.33075400
H	5.53219100	-3.69300900	-0.44538600
C	-2.92156000	-1.39963900	-0.00507900
C	-3.29844900	-2.61385200	-0.58261000
C	-3.90780200	-0.46083700	0.30613600
C	-4.63808000	-2.89643400	-0.82350700
H	-2.52541300	-3.32916000	-0.83663700
C	-5.24857600	-0.73691300	0.05315100
H	-3.63059400	0.50003900	0.72441200
C	-5.61715500	-1.95733300	-0.50607900
H	-4.91995000	-3.84668100	-1.26184200
H	-6.00474500	0.00308700	0.28786900
H	-6.66136000	-2.17321800	-0.69958000
H	-1.79753500	0.26343600	1.89138800
Li	-0.54133400	2.86376400	2.72836000

Zero-point correction= 0.334611 (Hartree/Particle)
Thermal correction to Energy= 0.358126
Thermal correction to Enthalpy= 0.359070
Thermal correction to Gibbs Free Energy= 0.279179
Sum of electronic and zero-point Energies= -1479.480726
Sum of electronic and thermal Energies= -1479.457211
Sum of electronic and thermal Enthalpies= -1479.456267
Sum of electronic and thermal Free Energies= -1479.536158

12Zc

S	-0.77216900	-0.59844600	1.21239400
O	-1.52781500	-1.71258100	0.58039600

O	-1.15514900	-0.26063800	2.58996200
C	0.90588500	-0.90117700	1.14174300
H	1.45996300	-0.53858700	1.99256700
C	1.45992200	-1.22391800	-0.09088900
C	-1.06317400	0.86298500	0.16854400
C	-2.20003400	1.07886100	-0.50188700
H	-2.19045900	1.95666500	-1.14742900
O	0.78147300	-1.45177500	-1.13875300
C	0.10088800	1.78068600	0.07853100
C	0.61282100	2.39165900	1.22715800
C	0.70697400	2.02075000	-1.15525100
C	1.71213900	3.23666100	1.13752600
H	0.14716200	2.19921500	2.18801700
C	1.80762500	2.86892600	-1.24216600
H	0.32361200	1.52540100	-2.04006900
C	2.31325500	3.47589900	-0.09699700
H	2.10042900	3.70990600	2.03154900
H	2.27449100	3.04659800	-2.20380700
H	3.17311200	4.13171000	-0.16391200
C	-3.47719200	0.34202200	-0.49218200
C	-4.17278600	0.19380900	-1.69699200
C	-4.05158600	-0.14179900	0.68760100
C	-5.39429200	-0.46960700	-1.73149600
H	-3.74797000	0.59556600	-2.61087600
C	-5.27982400	-0.79012800	0.65427400
H	-3.53841000	-0.00070600	1.63184300
C	-5.94987700	-0.96445900	-0.55479700
H	-5.91649700	-0.58940800	-2.67322600
H	-5.71788000	-1.15632400	1.57513700
H	-6.90725700	-1.47140000	-0.57716600
C	2.95598200	-1.26203100	-0.20876300
C	3.79016800	-1.49787100	0.88681600
C	3.52407600	-1.04394100	-1.46538100
C	5.17153300	-1.50904800	0.72680300
H	3.36226200	-1.69528300	1.86278800
C	4.90577700	-1.04196000	-1.62281800
H	2.86717300	-0.87005200	-2.30898400
C	5.73249100	-1.27566400	-0.52674100
H	5.81058600	-1.70420000	1.57979200
H	5.33815400	-0.86067400	-2.59983900
H	6.80921800	-1.28089100	-0.64887400
Li	-0.91522600	-2.26879300	-1.15816000

Zero-point correction= 0.335523 (Hartree/Particle)
Thermal correction to Energy= 0.358598
Thermal correction to Enthalpy= 0.359542
Thermal correction to Gibbs Free Energy= 0.280109
Sum of electronic and zero-point Energies= -1479.501249

Sum of electronic and thermal Energies= -1479.478174
Sum of electronic and thermal Enthalpies= -1479.477230
Sum of electronic and thermal Free Energies= -1479.556664

12Zd

S	-1.07170200	0.10081900	1.48288000
O	-1.79757300	-1.16400500	1.78663300
O	-1.33200800	1.21829100	2.39902100
C	0.60010400	-0.20430900	1.38236900
C	1.04091600	-1.24555000	0.57364600
C	-1.71424500	0.55389500	-0.16030000
C	-0.97615100	1.19759600	-1.06842100
H	-1.44209500	1.34547600	-2.04200900
O	0.27831900	-2.08685600	0.00821600
C	-3.09357600	0.08276200	-0.43787200
C	-3.35546400	-0.66678800	-1.58654200
C	-4.14326800	0.39191300	0.43279700
C	-4.65181400	-1.08719700	-1.87119200
H	-2.53820300	-0.92661400	-2.25054600
C	-5.43555500	-0.02967500	0.14704300
H	-3.94262100	0.96685000	1.33039600
C	-5.69361300	-0.76895700	-1.00607900
H	-4.84448800	-1.66807200	-2.76538200
H	-6.24382100	0.22045900	0.82381700
H	-6.70231600	-1.09784900	-1.22568300
C	0.39260600	1.73925700	-0.92050100
C	1.41143200	1.27368800	-1.75527600
C	0.67962600	2.71698300	0.03463200
C	2.71192900	1.73944700	-1.59936500
H	1.18923700	0.51919100	-2.50270100
C	1.97814400	3.19504000	0.17639500
H	-0.11344100	3.08346800	0.67709300
C	2.99855000	2.69881200	-0.63147600
H	3.50301600	1.34784000	-2.22855800
H	2.19425600	3.95022100	0.92294500
H	4.01235100	3.06204600	-0.51082700
C	2.51467400	-1.34433800	0.30257200
C	2.92829200	-2.00064700	-0.85792400
C	3.47420900	-0.77230900	1.14106600
C	4.27702900	-2.06078200	-1.19157400
H	2.17748600	-2.44758100	-1.49801900
C	4.82387700	-0.84239700	0.81438300
H	3.17269500	-0.28175700	2.05919300
C	5.22814000	-1.47890900	-0.35710100
H	4.58669500	-2.56037800	-2.10211700
H	5.56102200	-0.40114300	1.47483800

H	6.27988800	-1.52685100	-0.61399300
H	1.21777400	0.61421700	1.71248700
Li	-1.36171900	-2.69242400	0.69812000

Zero-point correction= 0.335551 (Hartree/Particle)
Thermal correction to Energy= 0.358500
Thermal correction to Enthalpy= 0.359444
Thermal correction to Gibbs Free Energy= 0.281435
Sum of electronic and zero-point Energies= -1479.503552
Sum of electronic and thermal Energies= -1479.480603
Sum of electronic and thermal Enthalpies= -1479.479659
Sum of electronic and thermal Free Energies= -1479.557668

12Ee

S	-0.13944800	-1.82992300	-0.31444200
O	-0.07123600	-2.54476500	0.99194000
O	0.25918300	-2.62394600	-1.48209500
C	-1.69619200	-1.18273200	-0.56412000
H	-1.96056900	-1.09493300	-1.60585700
C	-2.47549800	-0.71171700	0.48835000
C	1.07691200	-0.50217300	-0.16414000
C	0.64798700	0.76257500	-0.17646900
H	-0.41795300	0.92654700	-0.31631800
O	-2.12714300	-0.69044900	1.70449600
C	2.47859100	-0.98358800	-0.12607300
C	2.97261400	-1.71580900	0.95608400
C	3.32266900	-0.68686400	-1.20000200
C	4.30185100	-2.12740000	0.96971000
H	2.31664500	-1.95172500	1.78471000
C	4.64899400	-1.10092900	-1.18332900
H	2.93277000	-0.12401900	-2.04070300
C	5.14190400	-1.81967000	-0.09660400
H	4.68144300	-2.68772200	1.81582500
H	5.29707900	-0.86476200	-2.01886600
H	6.17641300	-2.14145500	-0.08301400
C	1.45808500	1.98812300	-0.05124900
C	0.95724700	3.16081100	-0.62954000
C	2.67550600	2.04539700	0.64029900
C	1.66895200	4.35258200	-0.55561700
H	0.00414700	3.13176600	-1.14672300
C	3.37863100	3.24057500	0.72539800
H	3.06665400	1.16014800	1.12564300
C	2.88416500	4.39465700	0.12107900
H	1.27106100	5.24765700	-1.01848900
H	4.31500400	3.27263400	1.26962900
H	3.43868400	5.32311200	0.18736900

C	-3.83270600	-0.15650200	0.15182000
C	-4.39227400	0.78625700	1.01674400
C	-4.55051600	-0.55589800	-0.97815700
C	-5.63847500	1.33992800	0.74510300
H	-3.83554300	1.08009300	1.89805600
C	-5.80312700	-0.01207900	-1.24253600
H	-4.14542500	-1.31088600	-1.64150500
C	-6.34707400	0.94135500	-0.38554600
H	-6.05860700	2.07963600	1.41637900
H	-6.35715200	-0.33701500	-2.11515400
H	-7.32133000	1.36699400	-0.59503800
Li	-0.97601800	-1.88847500	2.54439600

Zero-point correction= 0.335736 (Hartree/Particle)
Thermal correction to Energy= 0.358874
Thermal correction to Enthalpy= 0.359819
Thermal correction to Gibbs Free Energy= 0.279875
Sum of electronic and zero-point Energies= -1479.506874
Sum of electronic and thermal Energies= -1479.483736
Sum of electronic and thermal Enthalpies= -1479.482791
Sum of electronic and thermal Free Energies= -1479.562735

12Ef

S	-0.30902400	-0.78595700	-1.98701200
O	-0.13137800	-2.24943500	-2.24418000
O	-0.12722600	0.05971500	-3.17277300
C	-1.80271300	-0.62698600	-1.20179700
H	-2.32131100	-1.56044200	-1.05950600
C	-2.18790800	0.62590400	-0.69186600
C	0.99131500	-0.33574300	-0.82016200
C	1.58363600	0.84769800	-1.00516700
H	1.28443800	1.41466700	-1.88185000
O	-1.55402800	1.68464500	-0.86116000
C	1.29530400	-1.34032900	0.22573800
C	0.36019100	-1.70522700	1.19883800
C	2.56605600	-1.92553500	0.24533200
C	0.69490700	-2.64121100	2.17306100
H	-0.61966800	-1.24240200	1.19720600
C	2.89743300	-2.85896700	1.22113500
H	3.29445600	-1.63234100	-0.50312400
C	1.96115100	-3.21970000	2.18736800
H	-0.03372800	-2.91223100	2.92795400
H	3.88639400	-3.30191100	1.22910000
H	2.21889900	-3.94489400	2.95002900
C	2.62199200	1.46825900	-0.16292100
C	2.71627700	1.26077000	1.21956100
C	3.53606700	2.33298700	-0.77703000

C	3.71601100	1.88263800	1.95697400
H	1.99568500	0.62747300	1.72193900
C	4.54515000	2.94280100	-0.04044100
H	3.45437700	2.52080700	-1.84224800
C	4.63956300	2.71650700	1.32953400
H	3.77134900	1.72081700	3.02696900
H	5.25058800	3.60062100	-0.53390300
H	5.41984800	3.19635300	1.90823200
C	-3.46315700	0.67495100	0.12171200
C	-4.12662500	1.89936300	0.22508900
C	-3.98492500	-0.43581900	0.78895000
C	-5.29903800	2.01003400	0.96395700
H	-3.70688000	2.75864500	-0.28375800
C	-5.15253800	-0.32445500	1.53870200
H	-3.47206900	-1.38958200	0.74487600
C	-5.81509600	0.89699300	1.62394800
H	-5.80957400	2.96390100	1.02858900
H	-5.54207500	-1.19079400	2.06043200
H	-6.72568800	0.98211700	2.20535300
Li	1.11402800	-3.62149500	-2.26743100

Zero-point correction= 0.334986 (Hartree/Particle)
Thermal correction to Energy= 0.358437
Thermal correction to Enthalpy= 0.359381
Thermal correction to Gibbs Free Energy= 0.278987
Sum of electronic and zero-point Energies= -1479.488647
Sum of electronic and thermal Energies= -1479.465197
Sum of electronic and thermal Enthalpies= -1479.464252
Sum of electronic and thermal Free Energies= -1479.544646

12Eg

S	-0.20594300	-0.68992800	-2.15710300
O	-0.15277300	-2.14617000	-2.50507700
O	0.08613100	0.19880300	-3.28712900
C	-1.69248100	-0.43166000	-1.37912000
H	-2.31796900	-1.30683000	-1.31377600
C	-1.93959100	0.79057700	-0.73076100
C	1.08459000	-0.43761900	-0.92581100
C	1.88576000	0.61678700	-1.09523500
H	1.71118500	1.21576100	-1.98478200
O	-1.20130600	1.79243000	-0.78901300
C	0.99418500	-1.34663500	0.24006400
C	0.33160000	-0.91245600	1.39090900
C	1.55637800	-2.62434300	0.20697400
C	0.24936000	-1.74224400	2.50407800
H	-0.10878400	0.07934600	1.40196400

C	1.47372600	-3.45265700	1.32276400
H	2.07256200	-2.95453600	-0.68768600
C	0.82209600	-3.01180400	2.47208800
H	-0.26073500	-1.39837700	3.39614300
H	1.92317200	-4.43840100	1.29733600
H	0.76160400	-3.65590500	3.34138100
C	2.95171700	1.11216100	-0.20851800
C	3.36164900	2.44061900	-0.38519200
C	3.58046500	0.34429100	0.78211600
C	4.34735300	2.99922000	0.41947300
H	2.89284000	3.03936700	-1.15862100
C	4.57364800	0.90129400	1.57833200
H	3.30820800	-0.69288100	0.92479200
C	4.95604600	2.22993000	1.40636900
H	4.64290100	4.03105900	0.27216400
H	5.05468800	0.29329200	2.33535400
H	5.72913900	2.65888600	2.03270800
C	-3.20028900	0.87621900	0.10016000
C	-3.80782500	2.12441700	0.24982300
C	-3.75663100	-0.23097000	0.74444500
C	-4.96518000	2.26120400	1.00794200
H	-3.35957300	2.98120200	-0.23900500
C	-4.90760100	-0.09329100	1.51550600
H	-3.27766200	-1.20031200	0.66591600
C	-5.51800600	1.15132600	1.64355300
H	-5.43548800	3.23264300	1.10671600
H	-5.32454600	-0.95670200	2.02075700
H	-6.41648000	1.25722800	2.24018900
Li	-0.81406300	-3.76423100	-1.88263100

Zero-point correction= 0.334527 (Hartree/Particle)
Thermal correction to Energy= 0.358286
Thermal correction to Enthalpy= 0.359230
Thermal correction to Gibbs Free Energy= 0.276738
Sum of electronic and zero-point Energies= -1479.489392
Sum of electronic and thermal Energies= -1479.465632
Sum of electronic and thermal Enthalpies= -1479.464688
Sum of electronic and thermal Free Energies= -1479.547180

12Eh

S	-0.12584900	2.10852100	-0.78620200
O	-0.02830700	2.50050300	-2.22710700
O	0.27614000	3.28529500	0.04172800
C	-1.66114200	1.47375100	-0.50186800
H	-2.30008000	1.47515400	-1.36967900
C	-1.90247200	0.81573500	0.71937700

C	1.07799000	0.80700600	-0.47939400
C	2.06961900	1.07423000	0.37357800
H	2.05604100	2.04980900	0.85169700
O	-1.12261800	0.83671800	1.68957600
C	0.74528100	-0.47732000	-1.13744700
C	0.32820200	-1.55017800	-0.34466000
C	0.81933900	-0.62677100	-2.52323700
C	0.00654200	-2.76718400	-0.93365400
H	0.25432200	-1.41317100	0.72850400
C	0.49823700	-1.84793800	-3.10877000
H	1.13213700	0.21217200	-3.13396100
C	0.09377400	-2.91857100	-2.31611600
H	-0.31617800	-3.59620600	-0.31499700
H	0.56477300	-1.96300200	-4.18410300
H	-0.15635300	-3.86812000	-2.77438200
C	3.16871000	0.18301700	0.78013900
C	3.64590600	-0.87352900	-0.00774400
C	3.77796100	0.42798900	2.01706900
C	4.68846800	-1.67156000	0.44609200
H	3.21306500	-1.06439400	-0.98149600
C	4.81262200	-0.37934000	2.47508200
H	3.42713200	1.25457900	2.62544400
C	5.26922900	-1.43403200	1.69043200
H	5.05230200	-2.48076200	-0.17582600
H	5.26441400	-0.18167800	3.43969000
H	6.07923200	-2.06253600	2.04085700
C	-3.19062400	0.03953000	0.84201600
C	-3.73204800	-0.14485100	2.11578000
C	-3.83426500	-0.52596500	-0.26051000
C	-4.91084300	-0.86217400	2.28424000
H	-3.21459000	0.28345800	2.96600500
C	-5.00785800	-1.25495500	-0.09170600
H	-3.40650200	-0.42098100	-1.25100300
C	-5.55143500	-1.41944900	1.17954900
H	-5.32923300	-0.99042600	3.27574100
H	-5.49440600	-1.69977000	-0.95180300
H	-6.46736900	-1.98396100	1.30951700
Li	0.46199900	4.44291200	-1.62134300

Zero-point correction= 0.334159 (Hartree/Particle)
Thermal correction to Energy= 0.358019
Thermal correction to Enthalpy= 0.358963
Thermal correction to Gibbs Free Energy= 0.276301
Sum of electronic and zero-point Energies= -1479.490512
Sum of electronic and thermal Energies= -1479.466652
Sum of electronic and thermal Enthalpies= -1479.465708
Sum of electronic and thermal Free Energies= -1479.548370

Ring opened products 7:

***trans*-Diphenylethenyl sulfone 7E**

C	2.74167800	4.05812900	-0.18865600
C	1.75370000	3.13828400	-0.51887400
C	2.05531100	1.77607300	-0.63808600
C	3.38078800	1.35700000	-0.45986500
C	4.36913300	2.28074300	-0.14682500
C	4.05216900	3.63011500	-0.00019800
H	2.49032300	5.10699000	-0.08820600
H	0.73449900	3.47280700	-0.67932700
H	3.64351000	0.31393600	-0.58281900
H	5.39220300	1.94758500	-0.02030900
H	4.82695600	4.34524300	0.24918200
C	0.95264900	0.86440500	-0.97821500
H	0.11169400	1.32194400	-1.49338800
C	0.86490200	-0.44408000	-0.71273700
C	1.77746100	-1.28964000	0.09159000
C	2.47205200	-2.35826000	-0.48037700
C	1.95197100	-0.99248000	1.44689400
C	3.34986700	-3.10879900	0.29493400
H	2.32735000	-2.59064700	-1.52821000
C	2.82950300	-1.74676100	2.21668200
H	1.39336100	-0.17228300	1.88294700
C	3.53151200	-2.80322900	1.64105600
H	3.89324600	-3.93158700	-0.15381800
H	2.96350200	-1.51184900	3.26577400
H	4.21620500	-3.38998200	2.24184200
C	-1.50783200	-1.67137600	0.21702200
H	-2.30597400	-2.34129900	-0.10034300
H	-0.79709200	-2.20662600	0.84747400
C	-1.98091500	-0.41834500	0.94155000
O	-1.22623700	0.11496100	1.72483800
C	-3.34510800	0.11450300	0.67531600
C	-4.13936100	-0.34733300	-0.37757100
C	-3.82654900	1.12436100	1.51592600
C	-5.40104700	0.19855100	-0.58642500
H	-3.77701200	-1.10826400	-1.05654800
C	-5.08947600	1.65902600	1.31160200
H	-3.19980500	1.47381100	2.32720800
C	-5.87756600	1.19625600	0.25818500
H	-6.01044500	-0.15508300	-1.40877600
H	-5.46194000	2.43509600	1.96889500
H	-6.86372300	1.61517600	0.09652200

S	-0.61533200	-1.25960100	-1.30004600
O	-0.24683100	-2.52587200	-1.92958000
O	-1.42950600	-0.31086300	-2.05837700

Zero-point correction= 0.346472 (Hartree/Particle)
Thermal correction to Energy= 0.368651
Thermal correction to Enthalpy= 0.369595
Thermal correction to Gibbs Free Energy= 0.291314
Sum of electronic and zero-point Energies= -1472.470476
Sum of electronic and thermal Energies= -1472.448297
Sum of electronic and thermal Enthalpies= -1472.447353
Sum of electronic and thermal Free Energies= -1472.525635

***cis*-Diphenylethenyl sulfone 7Z**

C	4.51180500	-1.08752700	-1.63105700
C	3.30484000	-0.39884100	-1.63039600
C	2.98763900	0.46871400	-0.57874900
C	3.91085300	0.66763800	0.44947700
C	5.12606900	-0.00992100	0.43478500
C	5.42532500	-0.89369800	-0.59744700
H	4.74097500	-1.77022800	-2.44046400
H	2.59007700	-0.54676400	-2.43211500
H	3.67493400	1.34954700	1.25535600
H	5.83853600	0.15288900	1.23458700
H	6.37006400	-1.42424300	-0.60139300
C	1.70034500	1.19915700	-0.65161600
H	1.57811900	1.82001700	-1.53889000
C	0.63791000	1.16766400	0.15921400
C	-0.64095800	1.85059800	-0.16030900
C	-1.25532500	2.71430000	0.75137200
C	-1.23435800	1.62862700	-1.40570700
C	-2.43869500	3.35696300	0.41145300
H	-0.80337700	2.88379800	1.72105100
C	-2.42153300	2.27204300	-1.74166500
H	-0.77021800	0.93532800	-2.09949300
C	-3.02476400	3.13613900	-0.83386300
H	-2.90539600	4.03139000	1.11920000
H	-2.87676300	2.09019200	-2.70792300
H	-3.95089100	3.63540300	-1.09265100
C	0.56893000	-1.58726800	0.74432400
H	1.58550500	-1.74329000	0.38403500
H	0.33061400	-2.29416300	1.53996700
C	-0.38347700	-1.63842400	-0.44653600
O	0.09163000	-1.55020700	-1.55792600
C	-1.84980000	-1.76469700	-0.22958700
C	-2.40751300	-2.00382300	1.02878400
C	-2.68610600	-1.63087800	-1.34472900

C	-3.78701900	-2.11287300	1.16722300
H	-1.78239200	-2.09487800	1.90676600
C	-4.06086000	-1.73192700	-1.20236100
H	-2.24059400	-1.44031800	-2.31358700
C	-4.61222000	-1.97531900	0.05591100
H	-4.21594500	-2.30076400	2.14362900
H	-4.70494100	-1.62155100	-2.06612500
H	-5.68697500	-2.05600200	0.16834900
S	0.58468200	0.03234700	1.56106900
O	-0.69164400	0.21445100	2.24588300
O	1.80592000	0.10028200	2.35597500

Zero-point correction= 0.346180 (Hartree/Particle)
Thermal correction to Energy= 0.368287
Thermal correction to Enthalpy= 0.369231
Thermal correction to Gibbs Free Energy= 0.292120
Sum of electronic and zero-point Energies= -1472.461311
Sum of electronic and thermal Energies= -1472.439205
Sum of electronic and thermal Enthalpies= -1472.438260
Sum of electronic and thermal Free Energies= -1472.515372
